# Identifying modifiable factors and their joint associations on late-onset schizophrenia risk in the UK Biobank: a prospective exposure-wide association study

**DOI:** 10.1136/bmjment-2025-301954

**Published:** 2025-10-29

**Authors:** Fan Jiang, Qiuyue Dong, Emilio Fernandez-Egea, Rudolf N. Cardinal, Xinyu Li, Huizhi Liang, Wenbo Song, Alimu Dayimu, Haibo Wang, Lei Xu, Shanquan Chen

**Affiliations:** 1 Shandong ENT Hospital, School of Public Health, Shandong University, Jinan, Shandong, China; 2 Department of Psychiatry, University of Cambridge, Cambridge, UK; 3 Cambridgeshire and Peterborough NHS Foundation Trust, Cambridge, England, UK; 4 Behavioural and Clinical Neuroscience Institute, University of Cambridge, Cambridge, UK; 5 School of Computing, Newcastle University, Newcastle upon Tyne, England, UK; 6 Department of Non-communicable Disease Epidemiology, London School of Hygiene & Tropical Medicine, London, England, UK; 7 Cambridge Clinical Trials Unit Cancer Theme, University of Cambridge, Cambridge, England, UK; 8 School of Public Health, LKS Faculty of Medicine, University of Hong Kong, Hong Kong, China

**Keywords:** Schizophrenia

## Abstract

**Background:**

One in four cases of schizophrenia begins in late life, resulting in high unemployment and reduced life expectancy. However, knowledge of the modifiable risk factors for late-onset schizophrenia and their combined effects is limited.

**Aims:**

To identify modifiable risk factors for late-onset schizophrenia and estimate their joint disease risk effects.

**Methods:**

This prospective cohort study using UK Biobank data included 482 708 participants without late-onset schizophrenia at baseline, followed up for a mean of 14.36 years. We conducted an exposure-wide association study of 232 potentially modifiable factors linked to late-onset schizophrenia risk. Late-onset schizophrenia is diagnosed using ICD-10 (International Classification of Diseases, 10th Revision) criteria. Cox proportional hazard models identified significant factors across six domains: lifestyle, environment, medical history, physical measures, mental health and socioeconomic status (SES). Domain-specific weighted scores were calculated from Cox model coefficients and stratified into tertiles (favourable, intermediate, unfavourable) for risk assessment. Population attributable fractions (PAFs) quantified prevention potential.

**Results:**

During follow-up, 1276 participants developed late-onset schizophrenia. We identified 109 significant potentially modifiable factors, with intellectual disability (HR 35.15, 95% CI 11.23 to 110.09), manic episode (HR 33.14, 95% CI 21.16 to 51.90) and bipolar affective disorder (HR 32.91, 95% CI 27.07 to 40.01) showing the strongest risks, while higher household income (>£100 000: HR 0.14, 95% CI 0.09 to 0.22), regular friends/family visits (HR 0.23, 95% CI 0.18 to 0.28) and higher hand grip strength (HR 0.35, 95% CI 0.29 to 0.44) showed the strongest protection. PAF estimations indicated that shifting individuals from unfavourable to intermediate/favourable risk profiles could prevent 71.3% (95% CI 71.2% to 71.4%) of late-onset schizophrenia cases, mainly from mental health (25.1%, 95% CI 25.0% to 25.2%), medical history (13.6%, 95% CI 13.5% to 13.7%) and SES domain (11.2%, 95% CI 11.1% to 11.3%); shifting individuals from intermediate/unfavourable risk profiles to favourable could prevent 89.2% of cases.

**Conclusions:**

A substantial proportion of late-onset schizophrenia risk appears modifiable, with mental health and medical history as key contributors. Physical health and natural environment exposure provided protective benefits. Findings supported integrating clinical interventions and structural changes addressing socioeconomic and environmental factors to reduce late-onset schizophrenia burden.

**WHAT IS ALREADY KNOWN ON THIS TOPIC**

- Compared with early-onset schizophrenia, late-onset schizophrenia is more likely to be influenced by modifiable factors.

**WHAT THIS STUDY ADDS**

- In a prospective exposure-wide association study of 482 708 UK Biobank participants, we identified 109 potentially modifiable risk/protective factors across six domains: lifestyle, environment, medical history, physical measures, mental health and socioeconomic status.
- Shifting risk profiles towards favourable levels suggests up to 89% of late-onset schizophrenia cases might be preventable.

**HOW THIS STUDY MIGHT AFFECT RESEARCH, PRACTICE OR POLICY**

- Regular assessment and management of mental health conditions, medical comorbidities and socioeconomic disadvantage may be integrated into mid-life and late-life care to reduce the risk of late-onset schizophrenia and enable timely preventive interventions.

## Introduction

 Schizophrenia is a severe psychiatric disorder that poses substantial individual and societal burdens, with over 50% of diagnosed individuals experiencing long-term intermittent problems and approximately 20% developing chronic symptoms.^1^ Schizophrenia typically emerges in late adolescence and early adulthood, but one in four begins after age 40.^2^ Late-onset schizophrenia also causes major loss of function and social costs, such as high unemployment rates and shorter life expectancy.^1^ However, knowledge of actionable prevention strategies for late-onset schizophrenia remains relatively limited.^3^

Two of the most well-established risk factors for late-onset schizophrenia—genetic susceptibility and early neurodevelopmental abnormalities during gestation—are largely unmodifiable in adulthood.^4^ Some research also focused on the modifiable risk factors, including environmental exposures such as head injury,^5^ but there has been a lack of systematic research into the broad spectrum of modifiable factors for late-onset schizophrenia. Given that schizophrenia is a multifactorial disorder where single-target interventions are insufficient, there is a clear need for a more systematic approach to analyse the joint effects of modifiable exposures and guide effective prevention strategies.

Given these challenges, hypothesis-driven approaches to modifiable factors have several limitations. First, single-exposure analyses are likely to produce overestimated effect sizes and type I errors due to the interconnected nature of risk factors.^6^ Second, selective reporting constrains reproducibility.^7^ Third, investigating one or a handful of risk factors at a time cannot reflect the synergistic effects of exposures.^8^ Lastly, varying analytic choices and definitions hinder cross-study comparison.^7^

An exposure-wide association study (EWAS) is a hypothesis-free strategy that systematically investigates the relationship between multiple variables and a single outcome.^9^ By investigating a wide range of exposures simultaneously, EWAS validates established factors from previous studies with reduced bias while enabling the discovery of novel risk factors. Moreover, by constructing composite scores and calculating population attributable fraction (PAF), the joint effects of multiple risk factors can be assessed and their contributions to late-onset schizophrenia prevalence can be determined.

In this study, using data from nearly 500 000 UK Biobank (UKB) participants without schizophrenia at baseline, we aimed to (1) conduct an EWAS to comprehensively identify potentially modifiable risk factors; (2) combine risk factors to create composite scores for different domains to investigate their joint effects on late-onset schizophrenia; and (3) quantify the PAF for each domain and in total for late-onset schizophrenia to uncover the potential impact of preventative approaches. This systematic approach could help prioritise targets for multimodal prevention of late-onset schizophrenia.

## Methods

### Study design and participants

We conducted a prospective cohort study using the UKB, a large-scale population-based cohort comprising over 500 000 participants aged 40–69 years, enrolled between 2006 and 2010.^10^ Participants were recruited through 22 assessment centres distributed across England, Scotland and Wales. All participants provided written informed consent for the collection of questionnaire data and biological specimens. The study protocol was approved by the North West Multi-Center Research Ethics Committee (reference: 11/NW/0382) and conducted under UKB application number 107 217.

The study population excluded individuals with: (1) pre-existing schizophrenia diagnosis at baseline, (2) missing diagnostic or follow-up data, or (3) more than 20% missing data on potentially modifiable factor. The participant selection process was illustrated in online supplemental figure 1. All potentially modifiable exposures and covariates were assessed at baseline. Participant follow-up started at recruitment and continued until the first occurrence of one of the following events: schizophrenia diagnosis (primary outcome), death or loss to follow-up. This timeline ensured that exposures and covariates preceded the outcome and helped to minimise the risk of reverse causality.

### Outcome

The primary outcome was incident late-onset schizophrenia, defined as a new diagnosis identified through International Classification of Diseases, 10th Revision (ICD-10) codes F20–F29.^11^ These diagnoses were extracted from UKB health outcome datasets for first occurrences of heart outcomes (category 2405, including cases from hospital record, death registration and primary care). Detailed information was provided in online supplemental methods.

### Potentially modifiable factors or exposures

Briefly, modifiable factors were defined as risk factors that could be altered through individual behaviour changes, medical interventions or public health policies, in contrast to non-modifiable factors such as age, sex or genetic predisposition. We first reviewed all variables available in the UKB dataset with reference to prior literature and supporting evidence to identify potentially modifiable factors.^12^,^15^ Variables with more than 20% missing data were excluded. This systematic approach yielded 232 potentially modifiable factors, consisting of genuinely modifiable behaviours (eg, lifestyle, physical health), treatable but less directly modifiable diagnoses (eg, depression, diabetes) and potential prodromal indicators (eg, bipolar disorder, intellectual disability). With reference to the framework of determinants of mental health outlined in the WHO World Mental Health Report,^16^ these factors were subsequently categorised into six domains: (1) medical history, (2) local environment, (3) lifestyle factors, (4) mental health, (5) physical measurements and (6) socioeconomic status (SES). The whole procedure of variable selection and classification was supervised and validated by three specialists in psychiatry (SC, RC and EF-E). Detailed variable specifications were provided in online supplemental methods and tables 1–4.

Biomarkers were excluded from this analysis for two main reasons. First, including them would have substantially reduced the sample size, as they were only available for a subset of participants. Second, they were considered intermediate factors on the causal pathway rather than direct modifiable exposures.

To maintain comprehensive coverage of potential risk factors, we retained variables that exhibited potential collinearity within categories (eg, green space percentage within 1000 m buffer and green space percentage within 300 m buffer) and potential prodromal symptoms. Data preprocessing and cleaning procedures were reported in online supplemental methods and tables 1–4.

Online supplemental table 5 presented the missing values for exposure factors. The median proportion of missing data was 3.1%, with a mean of 6.0%. Twenty-one variables exhibited more than 10% missingness. Missing data were addressed using multiple imputation by chained equations, generating five imputed datasets with ten iterations. Detailed information on imputation was provided in online supplemental methods.

### Covariates

The analysis incorporated key covariates: age, sex, genetic risk and assessment centre location. Age at baseline was operationalised as a continuous variable, calculated from the difference between date of birth and initial assessment centre attendance. Genetic risk was stratified into low, moderate and high groups based on tertiles of the schizophrenia polygenic risk score (PRS). Further details on covariates could be found in online supplemental methods.

### Statistical analysis

#### Exposure-wide association analysis

We conducted an EWAS using Cox proportional hazard regression models to examine associations between baseline exposures and incident late-onset schizophrenia. Given the absence of a clearly defined latent or prodromal period for late-onset schizophrenia, we did not impose a specific lag time between exposure and outcome in our primary analysis to avoid misattributing causality or overlooking reverse causation. A conservative Bonferroni-corrected significance threshold (α=0.05/299=1.67×10^−4^) was applied. Models were adjusted for baseline age, sex, genetic risk and assessment centre. We did not employ a single multivariable model including all 232 exposures at this initial screening stage to avoid the risk of overfitting, which could compromise the model’s ability to generalise to new data. For variables violating the proportional hazards assumption (Schoenfeld’s residuals test, p<0.0001), we incorporated time-dependent interaction terms. Among significant potentially modifiable factors, we assessed their collinearity and eliminated one variable from each highly correlated pair (r^2^>0.9), which had been applied in previous study (detailed in online supplemental methods).^13^

#### Domain-specific risk score construction

Significant variables from the EWAS were categorised into six predefined domains: medical history, local environment, lifestyle factors, mental health, physical measurements and SES. Protective factors (hazard ratio [HR] <1) were reverse-coded to represent risk. Domain-specific weighted standardised scores were computed using β coefficients from the adjusted Cox models, with risk factors mutually adjusted (belonging to one domain) and with adjustment for age, sex, genetic risk and assessment centre. These scores were calculated by multiplying exposures (binary variables) by their respective β coefficients, summing the products and normalising by the total β coefficients of all the same domain.^17^ A higher score indicated greater exposure to risk factors within each domain. The resulting domain scores were stratified into tertiles, representing favourable, intermediate and unfavourable risk levels.

#### Domain-level association analysis

We implemented two Cox regression models to examine domain-level associations with incident late-onset schizophrenia. Model 1 assessed individual domain associations, adjusting for age, sex, genetic risk and assessment centre. Model 2 incorporated mutual adjustment across all six domains. The proportional hazards assumption was verified using Schoenfeld residuals.

#### Population attributable fraction estimation

PAF analyses were conducted to quantify the potential disease reduction achievable through risk factor modification. We employed two estimation approaches: (1) a conservative estimate combining intermediate and favourable profiles (eliminating the worst tertile of risk factors), and (2) a more comprehensive estimate combining intermediate and unfavourable profiles (eliminating the worst two tertiles).

PAFs were calculated using the formula $\frac{p\left(HR-1\right)}{p\left(HR-1\right)+1}$, where *p* was the prevalence of the risk factor in the population and *HR* was the risk of incident late-onset schizophrenia in worse tertiles compared with better tertiles adjusted for age, sex, genetic risk and assessment centre. To account for domain interdependence, we implemented principal component analysis to determine domain-specific weights, enabling the computation of both combined and individual weighted PAFs. This methodology addressed potential overestimation due to risk factor interactions. The 95% confidence intervals (CIs) were calculated using the bootstrap method with 1000 resamples. Detailed information on the calculation of weighted and overall weighted PAF was provided in online supplemental methods.

#### Subgroup and sensitivity analyses

We conducted stratified analyses across age groups (<65 and ≥65 years), sex and genetic susceptibility levels, applying Bonferroni correction to account for multiple comparisons. Given the potential for delayed schizophrenia diagnosis and concerns about reverse causation and the absence of a clearly defined latent or prodromal period for late-onset schizophrenia, we repeated our analysis separately for individuals with follow-up durations of at least 5 and 10 years. We conducted a sensitivity analysis excluding the mental health domain to address concerns that disorders like bipolar disorder and anxiety may represent prodromal schizophrenia and share genetic and neurobiological mechanisms with late-onset schizophrenia.

Finally, we conducted additional analyses incorporating genetic susceptibility as a seventh domain in PAF calculations to assess its contribution to late-onset schizophrenia risk within the broader framework of modifiable and non-modifiable factors.

All analyses were performed using R version 4.3.2, adhering to STROBE (Strengthening the Reporting of Observational Studies in Epidemiology) reporting guidelines (online supplemental methods).

## Results

### Study sample

The final analytical cohort comprised 482 708 participants from the initial UKB population of 502 165 (online supplemental figure 1). Included participants had a mean age of 56.56 years (SD 8.09) at baseline, with females constituting 54.27% of the cohort. During a mean follow-up period of 14.36 years (SD 2.24), 1276 cases of late-onset schizophrenia were identified. The detailed baseline characteristics of the participants were presented in online supplemental table 6.

### Exposure-wide association analysis

**Figure 1:**
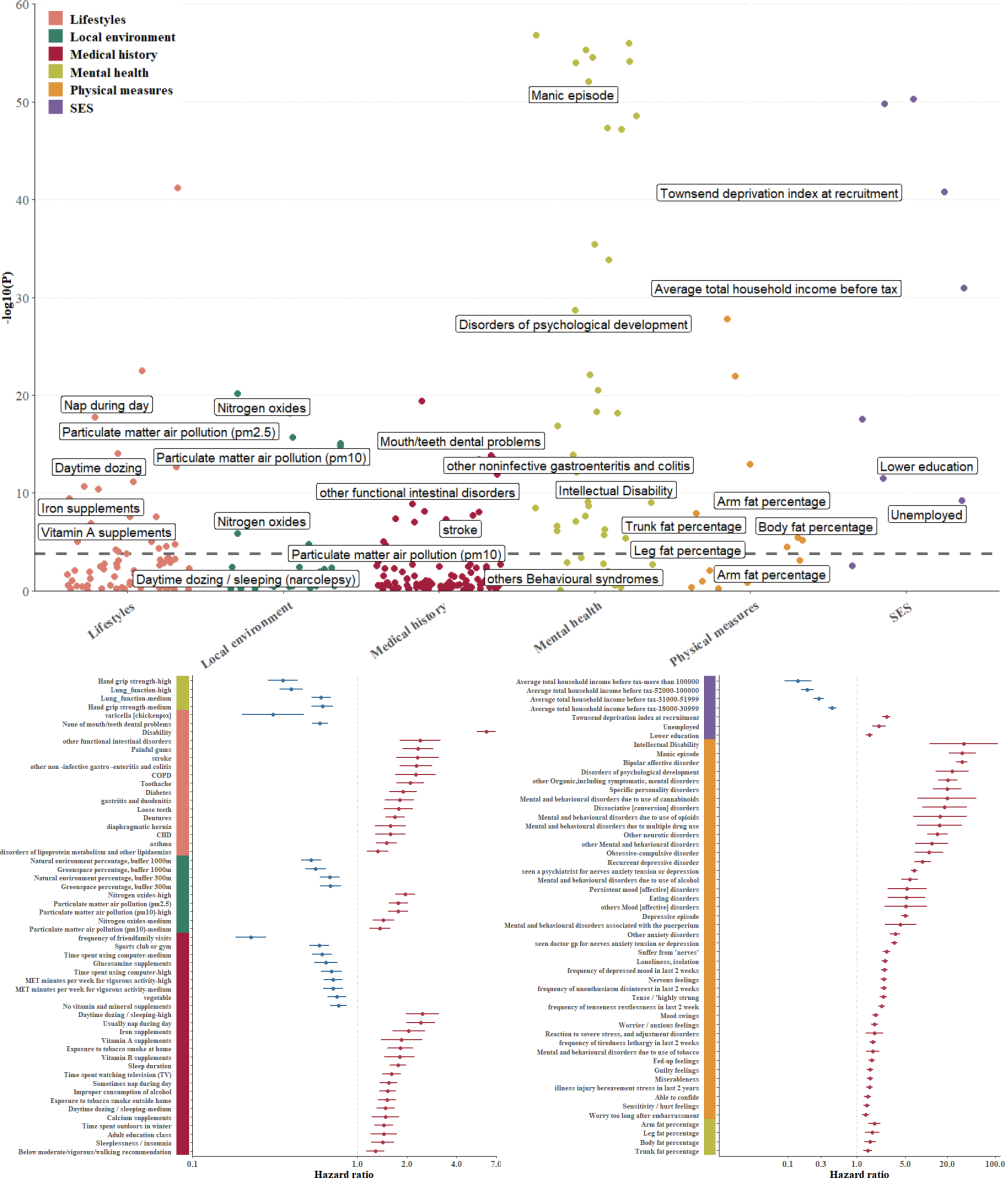
Associations between modifiable risk factors and incident late-onset schizophrenia. Panel A: The x axis shows the category domains and the y axis represents statistical significance (i.e., −log_10_ of the p value). The horizontal dotted line indicates threshold after correcting for multiple testing (Bonferroni corrected, p<1.67×10^–4^). A set of top risk factors was annotated. The full results are available in online supplemental table 7. Panel B: Significant factors in EWAS analysis were shown in panel B. Dots represent HRs, horizontal lines indicate corresponding 95% CIs. HRs were calculated using Cox proportional hazards regression analysis after adjustment for age, sex, genetic risk and assessment centre. Unit, hand grip strength (kg); blood pressure (mm Hg); time spent using computer (hours/day); health diet score was created from the cumulative sum of the level of consumption of components, including fruits, nuts, vegetables, whole grains, fish and dairy products, and a lower consumption of refined grains, processed meats, unprocessed red meats and sugar-sweetened beverages; vegetable intake (tablespoons/day); fruit intake (pieces/day); water intake (glasses/day); Townsend Deprivation Index was constructed from four census variables (households without a car, overcrowded households, households not owner-occupied and persons unemployed). EWAS, exposure-wide association study; SES, socioeconomic status; CHD, chronic heart disease; COPD, chronic obstructive pulmonary disease.

The exposure-wide analysis identified 109 significant risk factors (p<1.67×10^-4^) from the evaluated 232 exposures (figure 1, online supplemental table 7). Of these, 86 demonstrated detrimental associations and 23 showed protective effects. The strongest seven detrimental associations were observed for intellectual disability (HR 35.15, 95% CI 11.23 to 110.09), manic episode (HR 33.14, 95% CI 21.16 to 51.90), bipolar affective disorder (HR 32.91, 95% CI 27.07 to 40.01), disorders of psychological development (HR 23.73, 95% CI 13.67 to 41.20), other organic, including symptomatic, mental disorders (HR 20.63, 95% CI 14.93 to 28.51), specific personality disorders (HR 20.10, 95% CI 12.44 to 32.47), and mental and behavioural disorders due to use of cannabinoids (HR 20.05, 95% CI 7.50 to 53.59).

The seven most pronounced protective associations were observed for increasing household income brackets, friends/family visits, higher hand grip strength, enhanced lung function, increased natural environment percentage within 1000 m buffer, greater green space percentage within 1000 m buffer, and participating in sports club or gym.

**Figure 2:**
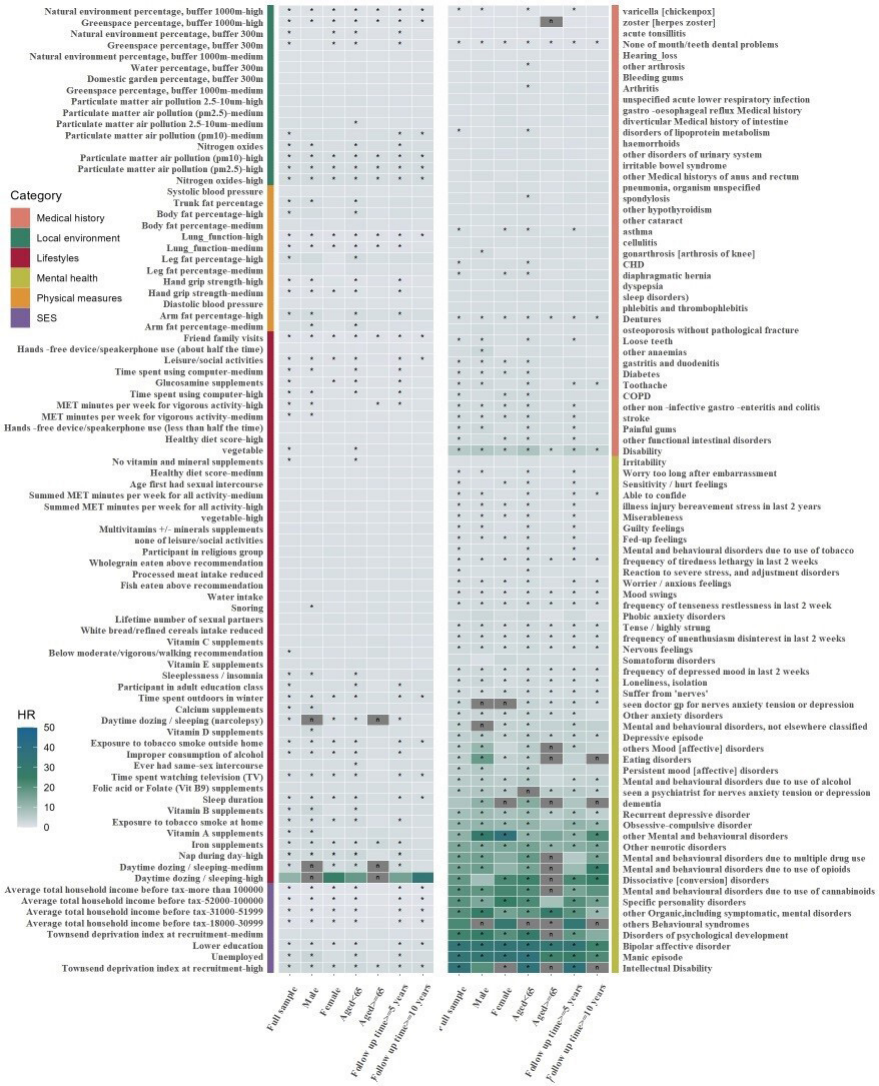
Summary heatmap for significant factors in EWAS analysis across the full sample and subgroups. Models were adjusted for baseline age, sex, genetic risk and assessment centre. The colour of cells indicates the effect sizes (HR) between each risk factor and incident schizophrenia. Asterisks in cells represent significant associations after correction for multiple testing (Bonferroni corrected, p<1.67×10^–4^); n in cells represents the HR for variable was not of reference significance due to the small sample size. Unit, hand grid strength (kg); blood pressure (mm Hg); time spent using computer (hours/day); health diet score was created from the cumulative sum of the level of consumption of components, including fruits, nuts, vegetables, whole grains, fish and dairy products, and a lower consumption of refined grains, processed meats, unprocessed red meats and sugar-sweetened beverages; vegetable intake (tablespoons/day); fruit intake (pieces/day); water intake (glasses/day); Townsend Deprivation Index was constructed from four census variables (households without a car, overcrowded households, households not owner-occupied and persons unemployed). EWAS, exposure-wide association study; SES; socioeconomic status; CHD, chronic heart disease; COPD, chronic obstructive pulmonary disease.

Collinearity analysis revealed no significant multicollinearity among the identified variables. The 109 significant factors were categorised into six domains: medical history (n=17), local environment (n=7), lifestyle (n=22), mental health (n=42), physical measures (n=6) and SES (n=4) (online supplemental table 7). Domain-specific EWAS results were presented in figure 1. Stratified analyses by age, sex, follow-up duration and genetic risk demonstrated consistent association patterns (figure 2, online supplemental tables 8 and 9).

### Joint associations of six domain-specific factors

**Figure 3:**
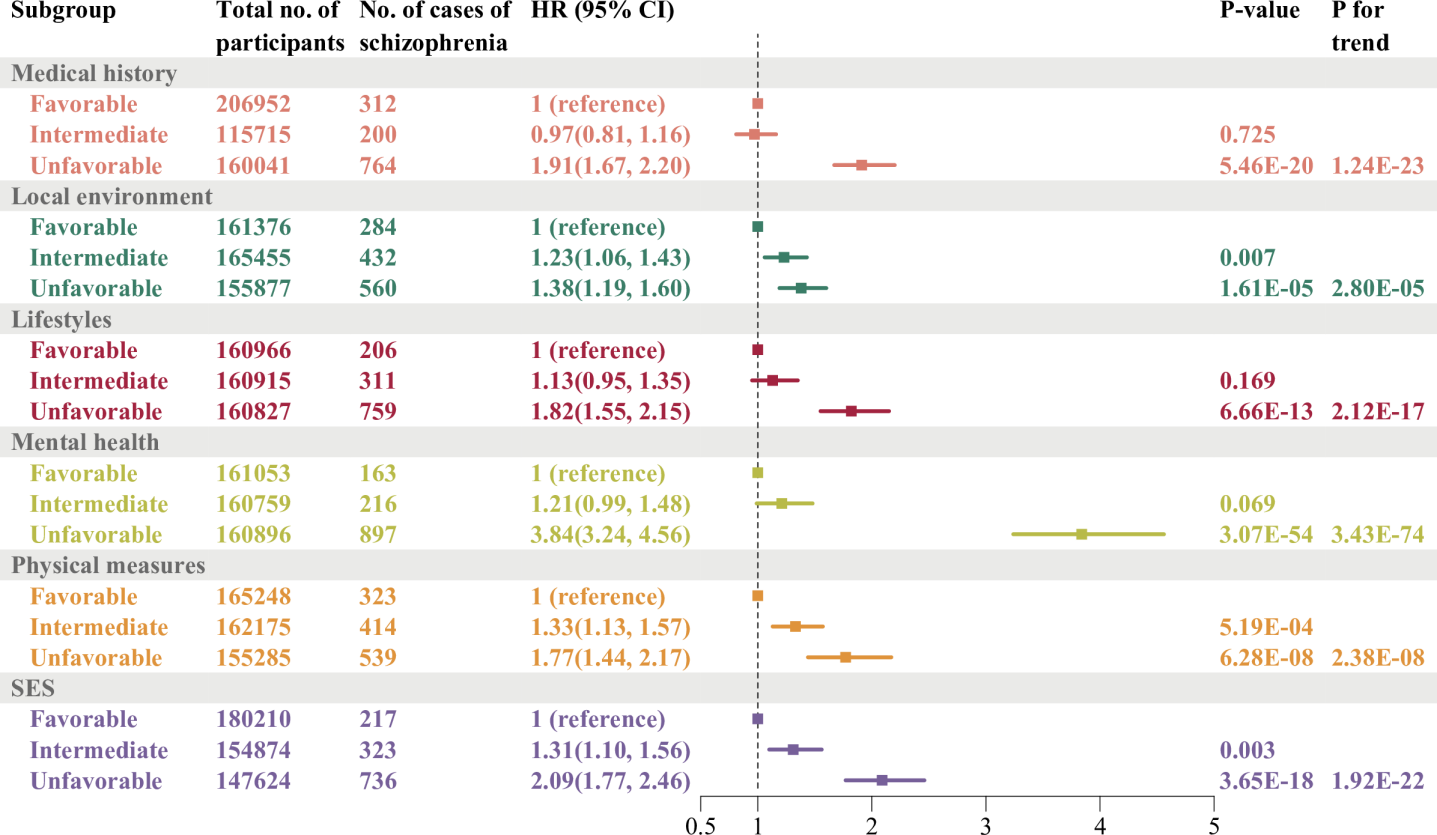
Associations between six domains and late-onset schizophrenia. Detailed in the Methods section, domain-specific risk scores were constructed, wherein higher scores denoted greater exposure to risk factors within each respective domain. These scores were subsequently stratified into tertiles to facilitate risk stratification, yielding favourable, intermediate and unfavourable risk categories. In this figure, the favourable profile was set as reference in each domain. The associations were estimated by applying a Cox model including all six domains after mutual adjustment for age, sex, genetic risk and assessment centre. Dots represent HRs; horizontal lines indicate corresponding 95% CIs. SES, socioeconomic status.

Compared with favourable profiles (figure 3, online supplemental table 10), intermediate risk profiles of three domains demonstrated significant associations with increased late-onset schizophrenia risk: local environment (HR 1.23, 95% CI 1.06 to 1.43), physical measures (HR 1.33, 95% CI 1.13 to 1.57) and SES (HR 1.31, 95% CI 1.10 to 1.56). Unfavourable profiles of all six domains demonstrated stronger significant associations in medical history (HR 1.91, 95% CI 1.67 to 2.20), local environment (HR 1.38, 95% CI 1.19 to 1.60), lifestyle factors (HR 1.82, 95% CI 1.55 to 2.15), mental health (HR 3.84, 95% CI 3.24 to 4.56), physical measures (HR 1.77, 95% CI 1.44 to 2.17) and SES (HR 2.09, 95% CI 1.77 to 2.46). The p values for trend of all domains were statistically significant.

The observed associations remained robust in both model 1 (adjusted for age, sex, genetic risk and assessment centre) (online supplemental table 10) and model 2 (mutually adjusted for all six domains) (figure 3).

Sensitivity and stratification analyses across age, sex, follow-up duration and genetic risk demonstrated consistent associations across all six domains (online supplemental tables 11–15). The sensitivity analysis excluding the mental health domain yielded similar results with main findings (online supplemental table 15).

### Population attributable fraction estimations

Two primary estimation approaches were implemented to quantify potential late-onset schizophrenia prevention through risk factor modification. Model 1 (shifting unfavourable to intermediate/favourable profiles) suggested a potential 71.3% (95%CI 71.2% to 71.4%) reduction in late-onset schizophrenia cases, while model 2 (shifting both unfavourable and intermediate to favourable profiles) indicated a potential 89.2% (95%CI 89.1% to 89.2%) reduction (table 1).

**Table 1:** Weighted and unweighted PAF for the six domains

Domains	Model 1			Model 2		
	Unweighted PAF	Communality	Weighted PAF	Unweighted PAF	Communality	Weighted PAF
Medical history	24.8 (24.7 to 25.0)	19.8	13.6 (13.5 to 13.7)	32.6 (32.4 to 32.9)	15.6	13.5 (13.4 to 13.5)
Local environment	7.7 (7.5 to 7.8)	4.3	4.2 (4.1 to 4.3)	21.3 (21.0 to 21.6)	9.1	8.8 (8.7 to 8.9)
Lifestyles	20.0 (19.8 to 20.1)	23.9	10.9 (10.9 to 11.0)	36.6 (36.3 to 36.9)	24.2	15.1 (15.0 to 15.2)
Mental health	45.8 (45.6 to 45.9)	11.1	25.1 (25.0 to 25.2)	55.4 (55.2 to 55.6)	6.2	22.8 (22.8 to 22.9)
Physical measures	11.5 (11.3 to 11.7)	12.7	6.3 (6.2 to 6.4)	29.4 (29.1 to 29.7)	14.3	12.1 (12.0 to 12.2)
SES	20.4 (20.2 to 20.6)	28.2	11.2 (11.1 to 11.3)	41.0 (40.7 to 41.2)	30.6	16.9 (16.8 to 17.0)
Overall weighted PAF			71.3 (71.2 to 71.4)			89.2 (89.1 to 89.2)

In model 1, we shifted the unfavourable profile to intermediate and favourable ones. In model 2, we shifted all factors to the favourable profile. All model adjusted for age, sex, assessment centre and genetic risk. Weighted PAF was calculated after considering overlap between risk factors.

PAF, population attributable fraction; SES, socioeconomic status.

In model 1, mental health demonstrated the highest preventive potential (PAF=25.1%, 95% CI 25.0% to 25.2%), followed by medical history (13.6%, 95% CI 13.5% to 13.7%), SES (11.2%, 95% CI 11.1% to 11.3%), lifestyle factors (10.9%, 95% CI 10.9% to 11.0%), physical measures (6.3%, 95% CI 6.2% to 6.4%) and local environment (4.2%, 95% CI 4.1% to 4.3%). In model 2, mental health remained the highest (22.8%, 95% CI 22.8% to 22.9%), followed by SES (16.9%, 95% CI 16.8% to 17.0%), lifestyle factors (15.1%, 95% CI 15.0% to 15.2%), medical history (13.5%, 95% CI 13.4% to 13.5%), physical measures (12.1%, 95% CI 12.0% to 12.2%) and local environment (8.8%, 95% CI 8.7% to 8.9%) (table 1).

When incorporating PRS (online supplemental table 16), model 1 indicated an increased preventive potential of 71.9%, with mental health showing the highest contribution (21.6%). Model 2 demonstrated an 86.9% preventive potential, with mental health maintaining the highest contribution (19.3%), consistent with the primary analysis.

Stratification analyses across age, sex, follow-up duration and genetic risk yielded similar PAF estimates for late-onset schizophrenia, with medical history and SES consistently emerging as the most influential domains (online supplemental tables 17–20). Furthermore, the sensitivity analysis excluding the mental health domain produced overall PAF estimates and domain-specific PAFs comparable to the primary findings (online supplemental table 21).

## Discussion

Our study systematically identified 86 detrimental factors and 23 protective factors associated with late-onset schizophrenia risk. The strongest risk associations were observed for intellectual disability, manic episode and bipolar affective disorder, while protective factors included higher income, friends/family visits, better grip strength and lung function, and greater exposure to natural environments. PAF analyses indicated that addressing these potentially modifiable factors had the potential to prevent between 71.3% and 89.2% of late-onset schizophrenia cases, with mental health showing the highest preventive potential (25.1%), followed by medical history (13.6%) and SES (11.2%).

Our findings consistently identify mental health as the most significant contributor to potentially preventable late-onset schizophrenia cases, with PAFs ranging from 21.6% to 25.1% across multiple estimations. Prior research suggested that mental health disorders increased late-onset schizophrenia risk through shared genetic vulnerabilities, neurodevelopmental abnormalities and dysregulation of common neural circuits.^18 19^ Severe mental disorders (e.g. intellectual disability and organic mental disorders) demonstrated the strongest associations, potentially linked to abnormalities in brain maturation, synaptic pruning and neural circuit formation during critical developmental periods.^4^ Mood/affective disorders emerged as the second most significant category, especially manic episodes and bipolar disorders. Genetic studies supported this link, showing overlapping genetic variations such as CACNA1C that regulate calcium signalling and synaptic plasticity affecting neurotransmitter systems.^20^ Substance use disorders, especially those related to cannabinoids and opioids, constituted the third major category. Previous research had established that cannabis use significantly increases schizophrenia risk.^21^ The mechanism likely involved disruption of the endocannabinoid system and subsequent alterations in dopaminergic signalling. Anxiety-related disorders, including conversion disorders and neurotic disorders, often serve as prodromal symptoms for schizophrenia. Meta-analyses showed that anxiety disorders tend to emerge before the onset of schizophrenia.^3^ This connection was believed to be mediated by the dysregulation of the hypothalamic-pituitary-adrenal (HPA) axis, which leads to chronically elevated levels of the stress hormone cortisol and increased inflammatory markers.^12^

Our findings suggested a strong association between SES and late-onset schizophrenia risk. A progressive association was found between higher income and the protective effect (HR ranging from 0.44 to 0.14). This aligned with previous research linking socioeconomic disadvantage to increased schizophrenia risk through chronic stress, limited access to resources and exposure to adverse environments.^7^ Chronic stress could lead to allostatic load, impacting neurodevelopmental trajectories and increasing neuroinflammation, while limited access to nutritious food could impair brain development and function.^22^

The association between physical health indicators like grip strength and lung function and a reduced risk of late-onset schizophrenia provided important insights into potential intervention targets. Grip strength, as a proxy for overall muscle strength, had been linked to brain health through mechanisms such as improved vascular integrity, reduced systemic inflammation and enhanced neural plasticity.^15^ Similarly, better lung function, indicative of cardiovascular and respiratory health, was associated with improved oxygenation of the brain, which directly supported neuronal metabolism, neurotransmitter synthesis and overall cognitive processes, potentially reducing vulnerability to psychosis.^23^

Our findings regarding natural environment exposure also provided novel insights. While prior research had primarily focused on urbanicity as a risk factor for schizophrenia,^24 25^ our results highlighted the protective effects of access to green spaces. Increased exposure to natural environments might reduce late-onset schizophrenia risk through decreased cortisol levels, lower inflammation levels and enhanced opportunities for physical activity and social interaction.^26^ Reduced cortisol levels indicated a healthier HPA axis regulation, while lower inflammation contributes to a less neurotoxic environment.^12^ Together, these mechanisms helped protect against the neurostructural and functional alterations associated with schizophrenia.^26^ Furthermore, natural environments might offer restorative benefits for cognitive functions by promoting parasympathetic nervous system activity and reducing cognitive load, which were often impaired in individuals at risk of schizophrenia.^12^

Lifestyle factors were important contributors to late-onset schizophrenia risk. Social engagement, particularly through friends and family visits, showed notable protective benefits through emotional support networks and reduced isolation.^27^ Similarly, physical activity, especially participation in organised sports or gym activities, appeared to help safeguard mental well-being through direct physical benefits and the social aspects of group exercise.^14^ Physical activity also promoted neurogenesis, enhanced synaptic plasticity and optimised neurotransmitter systems like dopamine and serotonin, all crucial for cognitive and emotional regulation.^28^ Furthermore, disrupted sleep schedules could heighten stress levels and impair overall mental health, while improper alcohol consumption might increase risk through effects on brain function and social stability.^14^

Our estimation of the PAF for late-onset schizophrenia risk factors exceeds prior assessments, which reflected our comprehensive, hypothesis-free approach. By evaluating a wide spectrum of potentially modifiable factors simultaneously, including some that have been underexplored in earlier studies, we were able to capture a broader picture of prevention potential. One consideration was that our high PAF could be partly due to the inclusion of potential prodromal symptoms within the mental health domain. However, our sensitivity analysis, which excluded the entire mental health domain, demonstrated that the PAF remained robust. Furthermore, the magnitude of our PAF was consistent with findings from recent EWAS studies that utilise similarly broad methods.^13^ Our model shows reliability in its components. The HR for the PRS (intermediate, 1.20; high, 2.03) aligned well with findings from previous research (odds ratio [OR] 1.69).^29^

Our results emphasised the need for a multidimensional approach to prevention, combining individual-focused interventions with broader structural changes. Together, these integrated strategies could create a supportive ecosystem that lowers the incidence of late-onset schizophrenia and enhances the overall well-being of communities. However, achieving an 89.2% reduction in cases presented considerable practical challenges, including limited resources, difficulties in implementing large-scale environmental modifications and the complex interplay of social and economic factors. Therefore, efforts should prioritise areas with strong preventive potential and practical intervention options before moving on to more complicated, large-scale changes.

Among the identified risk factors, feasible intervention strategies could be discussed in three categories. First, genuinely modifiable risk factors (e.g. physical activity, SES) could be intervened against at the individual, community and policy levels. For example, at the individual level, promoting regular physical activity, reducing excessive alcohol and cannabis use and ensuring sufficient sleep might improve mental health and lower late-onset schizophrenia risk. At community and policy level, interventions to improve social determinants such as education and poverty reduction, along with environmental improvements such as increasing access to green spaces and reducing urban stressors like noise and air pollution, might be equally critical for fostering mental health. Second, treatable but not necessarily modifiable diagnoses (eg, depression, anxiety) warrant early detection and effective clinical management, which could help reduce the subsequent risk of developing late-onset schizophrenia. Third, potential prodromal symptoms (e.g. bipolar disorder, intellectual disability), although less amenable to direct modification, might benefit from preventive strategies including genetic screening, prenatal counselling and early diagnostic interventions to reduce or delay the incidence of late-onset schizophrenia.

Our study had several important limitations. First, the UKB cohort might not fully represent the general population due to the self-selection of participants, who were often healthier and had higher SES.^30^ Previous studies comparing the UKB to other cohorts showed consistent risk factor patterns, validating our results. Second, the PAF estimates derived in this study were influenced by the prevalence of risk factors and late-onset schizophrenia incidence specific to the UKB. In particular, the findings might not be directly generalisable to non-European populations. Furthermore, healthcare systems differ widely across countries, and the reliance on the UK National Health Service for diagnostic and registry data means that case ascertainment and treatment patterns may not reflect those in other contexts. Third, the accuracy and scope of the data used in this study pose limitations. Many of the risk factors were self-reported, which could introduce recall bias or measurement errors. Future research should incorporate more precise diagnostic tools and clinically validated assessments to enhance the reliability of these findings. Fourth, reverse causality should be considered, given schizophrenia’s neurodevelopmental nature. Additionally, genetic predisposition may influence social outcomes, with those at higher genetic risk more likely to experience lower SES prior to disease onset. However, our sensitivity analyses stratified by genetic susceptibility and follow-up duration demonstrated consistent results, supporting the robustness of our findings. Fifth, our classification of modifiable factors warranted careful interpretation. While we defined modifiable factors as those potentially alterable through behavioural changes, medical interventions or public health policies, the degree of modifiability may vary substantially. Some identified factors, particularly certain mental health conditions, might not be strictly preventable but rather manageable through early intervention and treatment. A consideration here is that some of these conditions could represent prodromal symptoms of schizophrenia rather than causal risk factors, which could lead to an overestimation of the PAF. Nevertheless, our sensitivity analysis excluding the mental health domain, which would contain such symptoms, also demonstrated consistent findings, supporting the robustness of our conclusions beyond this potential bias. Lastly, while our analysis adjusted for collinearity among risk factors and accounted for interdependencies across domains, the relationships between these factors are inherently complex. Interaction effects and residual confounding may persist. To establish causality and clarify these relationships, future studies should include long-term longitudinal cohorts and hypothesis-driven clinical trials with comprehensive data on a wide range of covariates.

## Conclusions

This study highlighted the significant potential for late-onset schizophrenia prevention through addressing potentially modifiable risk factors at both individual and societal levels. By identifying and quantifying the contributions of a wide range of factors, our findings underscored the importance of integrated prevention strategies. While further validation in diverse populations is necessary, these results provided a foundation for targeted interventions that could substantially reduce late-onset schizophrenia incidence and improve overall mental health outcomes.

## Supplementary material

10.1136/bmjment-2025-301954 online supplemental file 1

## Data Availability

Data are available in a public, open access repository.
