# Supplementary material for: Identifying modifiable factors and their joint associations on late-onset schizophrenia risk in the UK Biobank: a prospective exposure-wide association study
Source: BMJ Ment Health. 2025 Oct 29;28(1):e301954. doi: 10.1136/bmjment-2025-301954 (PMC12574375; doi:10.1136/bmjment-2025-301954)
Supplement: online supplemental file 1 [file bmjment-28-1-s001.docx]

**Supplementary Methods**

**Source of outcome**

Clinical outcome and death status were ascertained through linkage to national hospital inpatient recorders (Hospital Episode Statistics for England, Scottish Morbidity Record for Scotland, and Patient Episode Database for Wales) and death register database (NHS Digital, NHS Central Register, and National Records). Data censoring occurred on October 30, 2022, for England, August 31, 2022, for Scotland, and May 31, 2022, for Wales. The late onset schizophrenia diagnoses were ascertained according to the ICD10 codes, extracted from UKB health outcome datasets first occurrences of hearth outcomes (Category 2405, including cases from hospital record, death registration and primary care).

**Data cleaning and processing**

Briefly, modifiable factors were defined as risk factors that could be altered through individual behavior changes, medical interventions, or public health policies, in contrast to non-modifiable factors such as age, sex, or genetic predisposition^[1, 2]^. From the initial 7214 available exposure variables, we excluded those with missing data >20% of the full sample and retained data collected at baseline. And then, based on the definition of modifiable factors, we then manually removed obviously unmodifiable exposure variable, which mainly came from the “Genomics”, “Cognitive function” and “Biological samples” categories. All exposure variables surviving data processing were summarized in Supplementary Table 1-4. Multiple-choice categorical variables (e.g., types of physical activity attended) were dummy coded into dichotomous variables (i.e., whether participated in one type of activity, assigning NA to those missing on the top-level array variable). Variables with only one level were removed. For diseases, we adopted data from “First occurrences” category, which was coded with 3-character ICD-10 codes and recorded the first date of diagnosis. For single-choice categorical variables, we reset the order of levels (following logical ordinal progression) and removed variables with only one level. For continuous variables, we divided them into tertiles after imputation.

Some variables were rearranged. For some dietary habits, we determined the recommendation intake level for fruit, fish, whole grains, etc. based on previous research^[3]^. A general healthy diet level score was also generated^[3]^. A set of diseases were generated from a combination of several 3-character ICD-10 codes^[4-8]^. Lung function was evaluated as the mean value of z-scores of FVC, FEV1 and PEF5^[9]^. The accurate evaluation of whether the participant had ever smoke^[10]^ and grams of alcohol intake^[11]^ was based on previous research. Moderate alcohol intake is considered to be 5-15 grams per day for women and 5-30 grams per day for men^[3]^. For some mental health conditions deprived from “First occurrences” category, we defined several mental health disorders together because the number of cases with some disorders was too small. Organic, including symptomatic, mental disorders was derived from codes F04-F09; Others Mental and behavioral disorders due to psychoactive substance use was derived from F14-F16 and F18; Others Mood [affective] disorders was derived from F38-F39; Others behavioral syndromes associated with physiological disturbances and physical factors was derived from F54-F59; Others disorders of adult personality and behavior was derived from F61-F65, F68-F69; Intellectual disability was derived from F70-F79; Disorders of psychological development was derived from F80-F89; Other mental disorder was derived from F90-F99.

The 232 potentially modifiable factors were categorized into six domains: (1) medical history (clinically confirmed chronic conditions such as diabetes, hypertension, and cardiovascular diseases), (2) local environment (characteristics of participants' surroundings, like air quality indices, noise levels, and access to green spaces), (3) lifestyle factors (health-related behaviors such as smoking status, alcohol consumption, and dietary patterns), (4) mental health (both diagnosed conditions like depression and anxiety, as well as measures of psychological well-being like stress levels), (5) physical measurements (objective health indicators obtained during assessment center visits, like blood pressure and grip strength), and (6) socioeconomic status (SES, economic and social factors such as household income, educational attainment, and occupational status).

**Covariates**

The polygenic risk scores (PRS) was computed using a Bayesian methodological framework, utilizing meta-analyzed data from three genome-wide association studies (GWAS) with a combined sample size of 97,456 cases and 334,331 controls^[12, 13]^. We did not include other potential covariates, such as early-life exposures, family history or neurocognitive function, for specific reasons. Early-life exposures would have led to a substantial reduction in sample size, as this data was only available for a subset of participants. We did not further adjust for family history, as genetic liability was already accounted for by PRS, which capture a large component of heritable risk. Similarly, neurocognitive function was not adjusted for because variables related to mental health were already part of potential modifiable factors, and including neurocognitive function could have resulted in over-adjustment for potential intermediate factors.

**Procedure used to select predictors for imputation**

We used the *quickpred* function from the *mice* package in R to select predictors for each variable. The function creates a predictor matrix as described by Van Buuren et al^[14]^. We modified the original function to ensure a maximum of 11 predictors (sex was forced to be included). This precaution allowed operation of this function with large sample sizes^[15]^.

**Test and treatment of collinearity**

For factors survived the EWAS analysis, collinearity was checked and we excluded one of two factors from a highly-associated pair (r^2^>0.9) from the *caret* package in R. There was no collinearity among all variables survived the EWAS analysis.

**Standard method for calculation of weighted and overall weighted PAF^[16]^**

To calculate the Population Attributable Fraction (PAF) for each domain, we first estimated individual PAFs. To account for overlap between multiple domains, we further calculated communality. Specifically, we input data on all domains into a correlation analysis to obtain correlation coefficients, followed by principal component analysis of the correlation matrix. Components with eigenvalues ≥1 were retained, and communality for each domain was calculated as the sum of squared factor loadings, representing the extent to which unobserved common factors explained each measured variable. Each domain’s PAF was then weighted by its communality using the formula: Weight (w)=1−communality. The overall PAF was derived by combining the weighted PAFs according to the multiplicative model: PAR _overall weighted_ = 1 – Π 1 – (w × PAR). Finally, each domain weighted PAFs were obtained as: domain weighted PAF= domain PAF/Σ(domain PAF)×Overall weighted PAF.

STROBE Statement—checklist of items that should be included in reports of observational studies

|  | | **Item No** | **Recommendation** | **Page  No** |
| --- | --- | --- | --- | --- |
| **Title and abstract** | 1 | (*a*) Indicate the study’s design with a commonly used term in the title or the abstract | | 1 |
|  |  | (*b*) Provide in the abstract an informative and balanced summary of what was done and what was found | | 2 |
| **Introduction** | | | | |
| Background/rationale | 2 | Explain the scientific background and rationale for the investigation being reported | | 4 |
| Objectives | 3 | State specific objectives, including any prespecified hypotheses | | 4 |
| **Methods** | | | | |
| Study design | 4 | Present key elements of study design early in the paper | | 4-5 |
| Setting | 5 | Describe the setting, locations, and relevant dates, including periods of recruitment, exposure, follow-up, and data collection | | 4-5 |
| Participants | 6 | (*a*) *Cohort study*—Give the eligibility criteria, and the sources and methods of selection of participants. Describe methods of follow-up  *Case-control study*—Give the eligibility criteria, and the sources and methods of case ascertainment and control selection. Give the rationale for the choice of cases and controls  *Cross-sectional study*—Give the eligibility criteria, and the sources and methods of selection of participants | | 4-5 |
|  |  | (*b*) *Cohort study*—For matched studies, give matching criteria and number of exposed and unexposed  *Case-control study*—For matched studies, give matching criteria and the number of controls per case | | NP |
| Variables | 7 | Clearly define all outcomes, exposures, predictors, potential confounders, and effect modifiers. Give diagnostic criteria, if applicable | | 5-6, sup |
| Data sources/ measurement | 8* | For each variable of interest, give sources of data and details of methods of assessment (measurement). Describe comparability of assessment methods if there is more than one group | | 5-6, sup |
| Bias | 9 | Describe any efforts to address potential sources of bias | | 5 |
| Study size | 10 | Explain how the study size was arrived at | | 4-5 |
| Quantitative variables | 11 | Explain how quantitative variables were handled in the analyses. If applicable, describe which groupings were chosen and why | | 5-6, sup |
| Statistical methods | 12 | (*a*) Describe all statistical methods, including those used to control for confounding | | 6-7 |
|  |  | (*b*) Describe any methods used to examine subgroups and interactions | | 7 |
|  |  | (*c*) Explain how missing data were addressed | | 6 |
|  |  | (*d*) *Cohort study*—If applicable, explain how loss to follow-up was addressed  *Case-control study*—If applicable, explain how matching of cases and controls was addressed  *Cross-sectional study*—If applicable, describe analytical methods taking account of sampling strategy | | 5 |
|  |  | (*e*) Describe any sensitivity analyses | | 7 |
| **Results** | | | | |
| Participants | 13* | (a) Report numbers of individuals at each stage of study—eg numbers potentially eligible, examined for eligibility, confirmed eligible, included in the study, completing follow-up, and analysed | | 8 |
|  |  | (b) Give reasons for non-participation at each stage | | Supplementary figure 1 |
|  |  | (c) Consider use of a flow diagram | | Supplementary figure 1 |
| Descriptive data | 14* | (a) Give characteristics of study participants (eg demographic, clinical, social) and information on exposures and potential confounders | | 8 |
|  |  | (b) Indicate number of participants with missing data for each variable of interest | | sup |
|  |  | (c) *Cohort study*—Summarise follow-up time (eg, average and total amount) | | 8 |
| Outcome data | 15* | *Cohort study*—Report numbers of outcome events or summary measures over time | | *8* |
|  |  | *Case-control study—*Report numbers in each exposure category, or summary measures of exposure | | *NP* |
|  |  | *Cross-sectional study—*Report numbers of outcome events or summary measures | | *NP* |
| Main results | 16 | (*a*) Give unadjusted estimates and, if applicable, confounder-adjusted estimates and their precision (eg, 95% confidence interval). Make clear which confounders were adjusted for and why they were included | | 8-9 |
|  |  | (*b*) Report category boundaries when continuous variables were categorized | | sup |
|  |  | (*c*) If relevant, consider translating estimates of relative risk into absolute risk for a meaningful time period | | *NP* |
| Other analyses | 17 | Report other analyses done—eg analyses of subgroups and interactions, and sensitivity analyses | | 9, sup |
| **Discussion** | | | | |
| Key results | 18 | Summarise key results with reference to study objectives | | 9 |
| Limitations | 19 | Discuss limitations of the study, taking into account sources of potential bias or imprecision. Discuss both direction and magnitude of any potential bias | | 11-12 |
| Interpretation | 20 | Give a cautious overall interpretation of results considering objectives, limitations, multiplicity of analyses, results from similar studies, and other relevant evidence | | 10-12 |
| Generalisability | 21 | Discuss the generalisability (external validity) of the study results | | 11-12 |
| **Other information** | | | | |
| Funding | 22 | Give the source of funding and the role of the funders for the present study and, if applicable, for the original study on which the present article is based | | 12 |

*Give information separately for cases and controls in case-control studies and, if applicable, for exposed and unexposed groups in cohort and cross-sectional studies.

**Note:** An Explanation and Elaboration article discusses each checklist item and gives methodological background and published examples of transparent reporting. The STROBE checklist is best used in conjunction with this article (freely available on the Web sites of PLoS Medicine at http://www.plosmedicine.org/, Annals of Internal Medicine at http://www.annals.org/, and Epidemiology at http://www.epidem.com/). Information on the STROBE Initiative is available at www.strobe-statement.org.

**Reference**

1. Guloksuz S., Pries L.K., Delespaul P., et al. Examining the independent and joint effects of molecular genetic liability and environmental exposures in schizophrenia: results from the EUGEI study. World Psychiatry, 2019; 18(2): 173-182. doi: 10.1002/wps.20629.

2. Hamlyn J., Duhig M., McGrath J., et al. Modifiable risk factors for schizophrenia and autism--shared risk factors impacting on brain development. Neurobiol Dis, 2013; 53: 3-9. doi: 10.1016/j.nbd.2012.10.023.

3. Bountziouka V., Musicha C., Allara E., et al. Modifiable traits, healthy behaviours, and leukocyte telomere length: a population-based study in UK Biobank. Lancet Healthy Longev, 2022; 3(5): e321-e331. doi: 10.1016/s2666-7568(22)00072-1.

4. Osler M., Christensen G.T., Mortensen E.L., et al. Hearing loss, cognitive ability, and dementia in men age 19-78 years. Eur J Epidemiol, 2019; 34(2): 125-130. doi: 10.1007/s10654-018-0452-2.

5. Dugravot A., Fayosse A., Dumurgier J., et al. Social inequalities in multimorbidity, frailty, disability, and transitions to mortality: a 24-year follow-up of the Whitehall II cohort study. Lancet Public Health, 2020; 5(1): e42-e50. doi: 10.1016/s2468-2667(19)30226-9.

6. Dandona L.,Dandona R. Revision of visual impairment definitions in the International Statistical Classification of Diseases. BMC Med, 2006; 4: 7. doi: 10.1186/1741-7015-4-7.

7. Sivco P., Plancikova D., Melichova J., et al. Traumatic brain injury related deaths in residents and non-residents of 30 European countries: a cross-sectional study. Sci Rep, 2023; 13(1): 7610. doi: 10.1038/s41598-023-34560-7.

8. Zhang Y., Liang J., Liu Q., et al. Birth Weight and Adult Obesity Index in Relation to the Risk of Hypertension: A Prospective Cohort Study in the UK Biobank. Front Cardiovasc Med, 2021; 8: 637437. doi: 10.3389/fcvm.2021.637437.

9. Wang J., Dove A., Song R., et al. Poor pulmonary function is associated with mild cognitive impairment, its progression to dementia, and brain pathologies: A community-based cohort study. Alzheimers Dement, 2022; 18(12): 2551-2559. doi: 10.1002/alz.12625.

10. Zhang Y.B., Chen C., Pan X.F., et al. Associations of healthy lifestyle and socioeconomic status with mortality and incident cardiovascular disease: two prospective cohort studies. Bmj, 2021; 373: n604. doi: 10.1136/bmj.n604.

11. Topiwala A., Ebmeier K.P., Maullin-Sapey T., et al. No safe level of alcohol consumption for brain health: observational cohort study of 25,378 UK Biobank participants. 2021.

12. Thompson D.J., Wells D., Selzam S., et al. *UK Biobank release and systematic evaluation of optimised polygenic risk scores for 53 diseases and quantitative traits*. in *medRxiv*. 2022.

13. Thompson D.J., Wells D., Selzam S., et al. A systematic evaluation of the performance and properties of the UK Biobank Polygenic Risk Score (PRS) Release. PLoS One, 2024; 19(9): e0307270. doi: 10.1371/journal.pone.0307270.

14. van Buuren S., Boshuizen H.C.,Knook D.L. Multiple imputation of missing blood pressure covariates in survival analysis. Stat Med, 1999; 18(6): 681-94. doi: 10.1002/(sici)1097-0258(19990330)18:6<681::aid-sim71>3.0.co;2-r.

15. Ganna A.,Ingelsson E. 5 year mortality predictors in 498,103 UK Biobank participants: a prospective population-based study. Lancet, 2015; 386(9993): 533-40. doi: 10.1016/s0140-6736(15)60175-1.

16. Norton S., Matthews F.E., Barnes D.E., et al. Potential for primary prevention of Alzheimer's disease: an analysis of population-based data. Lancet Neurol, 2014; 13(8): 788-94. doi: 10.1016/s1474-4422(14)70136-x.


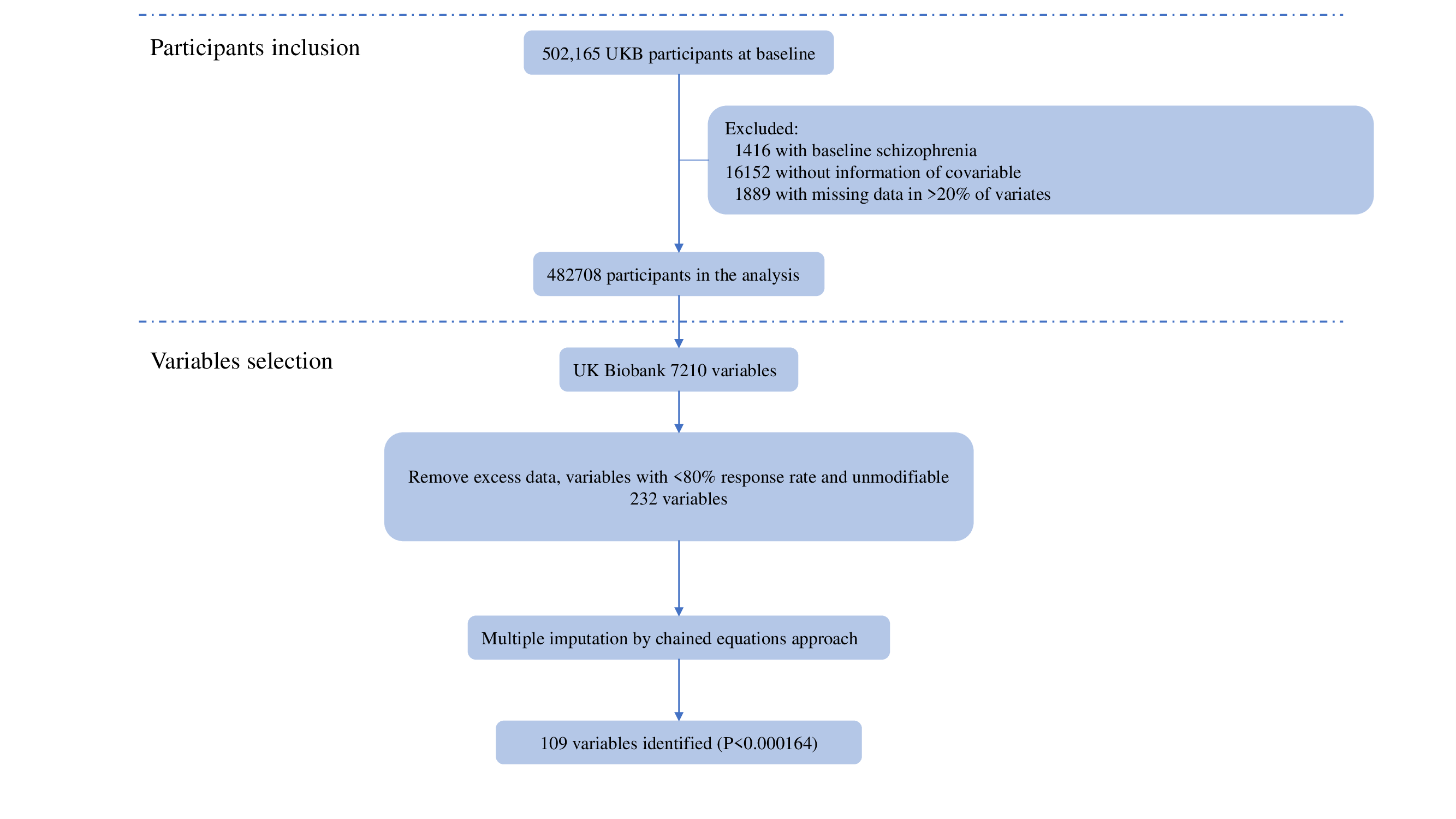


**Supplementary Figure 1. Overview of analytic design.** Analytic procedure to identify potentially modifiable risk factors associated with incident schizophrenia in the UK Biobank.

| **Supplementary Table 1. The coding and definition of the potentially modifiable factors.** FieldID, the index number of each variable; Field, the detail meaning of each variable; Data-coding, the index number of each variable’s coding; Encoding, the coding of each class of variable; Label, the meaning of each class of variable; Unit, the unit of the variable; Coding notes, the recoding of the variable. | | | | | | |  |
| --- | --- | --- | --- | --- | --- | --- | --- |
| **FieldID** | **Field** | **Data-coding** | **Encoding** | **Label** | **Unit** | **Coding notes** | |
| **Lifestyles** | | | | | | | |
| 1289 | Cooked vegetable intake | 100373 | -1 | Do not know | tablespoons/day | set as NA | |
|  |  |  | -10 | Less than one |  | set as 0.5 | |
|  |  |  | -3 | Prefer not to answer |  | set as NA | |
|  |  |  |  |  |  | Keep the original values as continuous variable | |
| 1299 | Salad / raw vegetable intake | 100373 | same as above | | tablespoons/day |  | |
| 1309 | Fresh fruit intake | 100373 | same as above | | pieces/day |  | |
| 1319 | Dried fruit intake | 100373 | same as above | | pieces/day |  | |
| 1329 | Oily fish intake | 100377 | -1 | Do not know |  | set as NA | |
|  |  |  | -3 | Prefer not to answer |  | set as NA | |
|  |  |  | 0 | Never |  | 0 | |
|  |  |  | 1 | Less than once a week |  | 0 | |
|  |  |  | 2 | Once a week |  | 1 | |
|  |  |  | 3 | 2-4 times a week |  | 1 | |
|  |  |  | 4 | 5-6 times a week |  | 1 | |
|  |  |  | 5 | Once or more daily |  | 1 | |
| 1339 | Non-oily fish intake | 100377 | same as above | |  |  | |
| 1438 | Bread intake | 100373 | same as above | | slices/week |  | |
| 1448 | Bread type | 100391 | -1 | Do not know |  | set as NA | |
|  |  |  | -3 | Prefer not to answer |  | set as NA | |
|  |  |  | 1 | White |  |  | |
|  |  |  | 2 | Brown |  |  | |
|  |  |  | 3 | Wholemeal or wholegrain |  |  | |
|  |  |  | 4 | Other type of bread |  |  | |
| 1458 | Cereal intake | 100373 | same as above | | bowls/week |  | |
| 1468 | Cereal type | 100393 | -1 | Do not know |  | set as NA | |
|  |  |  | -3 | Prefer not to answer |  | set as NA | |
|  |  |  | 1 | Bran cereal (e.g. All Bran, Branflakes) |  | 1 | |
|  |  |  | 2 | Biscuit cereal (e.g. Weetabix) |  | 0 | |
|  |  |  | 3 | Oat cereal (e.g. Ready Brek, porridge) |  | 1 | |
|  |  |  | 4 | Muesli |  | 0 | |
|  |  |  | 5 | Other (e.g. Cornflakes, Frosties) |  | 0 | |
| 1369 | Beef intake | 100377 | 0 | Never |  | 1 | |
|  |  |  | 1 | Less than once a week |  | 1 | |
|  |  |  | 2 | Once a week |  | 0 | |
|  |  |  | 3 | 2-4 times a week |  | 0 | |
|  |  |  | 4 | 5-6 times a week |  | 0 | |
|  |  |  | 5 | Once or more daily |  | 0 | |
| 1379 | Lamb/mutton intake | 100377 | same as above | |  |  | |
| 1389 | Pork intake | 100377 | same as above | |  |  | |
| 1349 | Processed meat intake | 100377 | same as above | |  |  | |
| 2644 | Light smokers, at least 100 smokes in lifetime | 100349 | -1 | Do not know |  | set as NA | |
|  |  |  | -3 | Prefer not to answer |  | set as NA | |
|  |  |  | 0 | No |  |  | |
|  |  |  | 1 | Yes |  |  | |
| 1249 | Past tobacco smoking | 100348 | -3 | Prefer not to answer |  | set as NA | |
|  |  |  | 1 | Smoked on most or all days |  |  | |
|  |  |  | 2 | Smoked occasionally |  |  | |
|  |  |  | 3 | Just tried once or twice |  |  | |
|  |  |  | 4 | I have never smoked |  |  | |
| 1239 | Current tobacco smoking | 100347 | -3 | Prefer not to answer |  | set as NA | |
|  |  |  | 0 | No |  |  | |
|  |  |  | 1 | Yes, on most or all days |  |  | |
|  |  |  | 2 | Only occasionally |  |  | |
| 1160 | Sleep duration | 100291 |  |  | hours/day | set -1 -3 as NA Improper sleep duration <7/>9 hours | |
| 1568 | Average weekly red wine intake | 100291 |  |  | glasses | set -1 -3 as NA | |
| 4407 | Average monthly red wine intake | 100291 |  |  | glasses | set -1 -3 as NA | |
| 1578 | Average weekly champagne plus white wine intake | 100291 |  |  | glasses | set -1 -3 as NA | |
| 4418 | Average monthly champagne plus white wine intake | 100291 |  |  | glasses | set -1 -3 as NA | |
| 1588 | Average weekly beer plus cider intake | 100291 |  |  | pints | set -1 -3 as NA | |
| 4429 | Average monthly beer plus cider intake | 100291 |  |  | pints | set -1 -3 as NA | |
| 1598 | Average weekly spirits intake | 100291 |  |  | measures | set -1 -3 as NA | |
| 4440 | Average monthly spirits intake | 100291 |  |  | measures | set -1 -3 as NA | |
| 1608 | Average weekly fortified wine intake | 100291 |  |  | glasses | set -1 -3 as NA | |
| 4451 | Average monthly fortified wine intake | 100291 |  |  | glasses | set -1 -3 as NA | |
| 5364 | Average weekly intake of other alcoholic drinks | 100291 |  |  | glasses | set -1 -3 as NA | |
| 4462 | Average monthly intake of other alcoholic drinks | 100291 |  |  | glasses | set -1 -3 as NA | |
| 1558 | Alcohol intake frequency. | 100402 | -3 | Prefer not to answer |  | set as NA | |
|  |  |  | 1 | Daily or almost daily |  |  | |
|  |  |  | 2 | Three or four times a week |  |  | |
|  |  |  | 3 | Once or twice a week |  |  | |
|  |  |  | 4 | One to three times a month |  |  | |
|  |  |  | 5 | Special occasions only |  |  | |
|  |  |  | 6 | Never |  |  | |
|  | | | | | | | |

| **Supplementary Table 2. Single-choice categorical variables and processing.** FieldID, the index number of each variable; Field, the detail meaning of each variable; Data-coding, the index number of each variable’s coding; Encoding, the coding of each class of variable; Label, the meaning of each class of variable; Coding notes, the recoding of the variable. | | | | | |
| --- | --- | --- | --- | --- | --- |
| **FieldID** | **Field** | **Data-coding** | **Encoding** | **Label** | **coding notes** |
| **SES** | | | | | |
| 738 | Average total household income before tax | 100294 | -1 | Do not know | set as NA |
|  |  |  | -3 | Prefer not to answer | set as NA |
|  |  |  | 1 | Less than 18,000 |  |
|  |  |  | 2 | 18,000 to 30,999 |  |
|  |  |  | 3 | 31,000 to 51,999 |  |
|  |  |  | 4 | 52,000 to 100,000 |  |
|  |  |  | 5 | Greater than 100,000 |  |
| **Lifestyles** | | | | | |
| 1210 | Snoring | 100345 | -1 | Do not know | set as NA |
|  |  |  | -3 | Prefer not to answer | set as NA |
|  |  |  | 2 | Yes |  |
|  |  |  | 1 | No |  |
| Derived | Ever smoke |  | 2 | Yes | Original data (FieldID 2644) are listed in Supplementary Table1, details in ref^[10]^ |
|  |  |  | 1 | No |  |
| Derived | Moderate drinking |  | 2 | No | Original data (FieldID 1568 4407 1578 4418 1588 4429 1598 4440 1608 4451 5364 4462) are listed in Supplementary Table1, details in ref^[11]^ |
|  |  |  | 1 | Yes |  |
| 2159 | Ever had same-sex intercourse | 100352 | -3 | Prefer not to answer | set as NA |
|  |  |  | 0 | No |  |
|  |  |  | 1 | Yes |  |
| 22036 | Above moderate/vigorous/walking recommendation | 7 | 2 | No |  |
|  |  |  | 1 | Yes |  |
| Derived | Moderate sleep |  | 2 | No | Original data (FieldID 1160) are listed in Supplementary Table1 |
|  |  |  | 1 | Yes |  |
| 1190 | Nap during day | 100343 | -3 | Prefer not to answer | set as NA |
|  |  |  | 1 | Never/rarely |  |
|  |  |  | 2 | Sometimes |  |
|  |  |  | 3 | Usually |  |
|  |  |  |  |  |  |
| 1200 | Sleeplessness / insomnia | 100343 | -3 | Prefer not to answer | set as NA |
|  |  |  | 1 | Never/rarely |  |
|  |  |  | 2 | Sometimes |  |
|  |  |  | 3 | Usually |  |
| 1220 | Daytime dozing / sleeping (narcolepsy) | 100346 | -1 | Do not know | set as NA |
|  |  |  | -3 | Prefer not to answer | set as NA |
|  |  |  | 0 | Never/rarely |  |
|  |  |  | 1 | Sometimes |  |
|  |  |  | 2 | Often |  |
|  |  |  | 3 | All of the time |  |
| 1269 | Exposure to tobacco smoke at home | 100291 | set -1 -3 as NA | | Turn data into binary classification：1：exposure to tobacco smoke at home; 0: no exposure |
| 1279 | Exposure to tobacco smoke outside home | 100291 |  |  | set -1 -3 as NA; Turn data into binary classification：1：exposure to tobacco smoke outside; 0: no exposure |
| 1120 | Weekly usage of mobile phone in last 3 months | 100336 | -1 | Do not know | set as NA |
|  |  |  | -3 | Prefer not to answer | set as NA |
|  |  |  | 0 | Less than 5mins |  |
|  |  |  | 1 | 5-29 mins |  |
|  |  |  | 2 | 30-59 mins |  |
|  |  |  | 3 | 1-3 hours |  |
|  |  |  | 4 | 4-6 hours |  |
|  |  |  | 5 | More than 6 hours |  |
| 1130 | Hands-free device/speakerphone use with mobile phone in last 3 month | 100337 | -1 | Do not know | set as NA |
|  |  |  | -3 | Prefer not to answer | set as NA |
|  |  |  | 0 | Never or almost never |  |
|  |  |  | 1 | Less than half the time |  |
|  |  |  | 2 | About half the time |  |
|  |  |  | 3 | More than half the time |  |
|  |  |  | 4 | Always or almost always |  |
| Derived | Fruit eaten above recommendation |  |  |  | Original data (FieldID 1309 1319) are listed in Supplementary Table1, details in ref ^[3]^ |
| Derived | Fish eaten above recommendation |  |  |  | Original data (FieldID 1329 1339) are listed in Supplementary Table1, details in ref^[3]^ |
| Derived | White bread/refined cereals intake reduced |  |  |  | Original data (FieldID 1438 1448) are listed in Supplementary Table1, details in ref^[3]^ |
| Derived | Wholegrain eaten above recommendation |  |  |  | Original data (FieldID 1458 1468) are listed in Supplementary Table1, details in ref^[3]^ |
| Derived | Red meat intake reudced |  |  |  | Original data (FieldID 1369 1379 1389) are listed in Supplementary Table1, details in ref^[3]^ |
| Derived | Processed meat intake reduced |  |  |  | Original data (FieldID 1349) are listed in Supplementary Table1, details in ref^[3]^ |
| **Medical history** | | | | | |
| 130176 | Date B01 first reported (varicella [chickenpox]) |  |  |  |  |
| 130178 | Date B02 first reported (zoster [herpes zoster]) |  |  |  |  |
| 130188 | Date B07 first reported (viral warts) |  |  |  |  |
| 130226 | Date B35 first reported (dermatophytosis) |  |  |  |  |
| 130622 | Date D50 first reported (iron deficiency anaemia) |  |  |  |  |
| 130648 | Date D64 first reported (other anaemias) |  |  |  |  |
| 130696 | Date E03 first reported (other hypothyroidism) |  |  |  |  |
| 130792 | Date E66 first reported (obesity) |  |  |  |  |
| 130814 | Date E78 first reported (disorders of lipoprotein metabolism and other lipidaemias) |  |  |  |  |
| 131052 | Date G43 first reported (migraine) |  |  |  |  |
| 131060 | Date G47 first reported (sleep disorders) |  |  |  |  |
| 131074 | Date G56 first reported (mononeuropathies of upper limb) |  |  |  |  |
| 131128 | Date H00 first reported (hordeolum and chalazion) |  |  |  |  |
| 131142 | Date H10 first reported (conjunctivitis) |  |  |  |  |
| 131166 | Date H26 first reported (other cataract) |  |  |  |  |
| 131222 | Date H60 first reported (otitis externa) |  |  |  |  |
| 131224 | Date H61 first reported (other disorders of external ear) |  |  |  |  |
| 131262 | Date H92 first reported (otalgia and effusion of ear) |  |  |  |  |
| 131264 | Date H93 first reported (other disorders of ear, not elsewhere classified) |  |  |  |  |
| 131350 | Date I48 first reported (atrial fibrillation and flutter) |  |  |  |  |
| 131396 | Date I80 first reported (phlebitis and thrombophlebitis) |  |  |  |  |
| 131402 | Date I83 first reported (varicose veins of lower extremities) |  |  |  |  |
| 131404 | Date I84 first reported (haemorrhoids) |  |  |  |  |
| 131426 | Date J01 first reported (acute sinusitis) |  |  |  |  |
| 131428 | Date J02 first reported (acute pharyngitis) |  |  |  |  |
| 131430 | Date J03 first reported (acute tonsillitis) |  |  |  |  |
| 131436 | Date J06 first reported (acute upper respiratory infections of multiple and unspecified sites) |  |  |  |  |
| 131442 | Date J11 first reported (influenza, virus not identified) |  |  |  |  |
| 131456 | Date J18 first reported (pneumonia, organism unspecified) |  |  |  |  |
| 131462 | Date J22 first reported (unspecified acute lower respiratory infection) |  |  |  |  |
| 131464 | Date J30 first reported (vasomotor and allergic rhinitis) |  |  |  |  |
| 131468 | Date J32 first reported (chronic sinusitis) |  |  |  |  |
| 131472 | Date J34 first reported (other disorders of nose and nasal sinuses) |  |  |  |  |
| 131494 | Date J45 first reported (asthma) |  |  |  |  |
| 131584 | Date K21 first reported (gastro-oesophageal reflux Medical history) |  |  |  |  |
| 131598 | Date K29 first reported (gastritis and duodenitis) |  |  |  |  |
| 131600 | Date K30 first reported (dyspepsia) |  |  |  |  |
| 131612 | Date K40 first reported (inguinal hernia) |  |  |  |  |
| 131620 | Date K44 first reported (diaphragmatic hernia) |  |  |  |  |
| 131630 | Date K52 first reported (other non-infective gastro-enteritis and colitis) |  |  |  |  |
| 131636 | Date K57 first reported (diverticular Medical history of intestine) |  |  |  |  |
| 13163 | Date K58 first reported (irritable bowel syndrome) |  |  |  |  |
| 131640 | Date K59 first reported (other functional intestinal disorders) |  |  |  |  |
| 131646 | Date K62 first reported (other Medical historys of anus and rectum) |  |  |  |  |
| 131650 | Date K64 first reported (haemorrhoids and perianal venous thrombosis) |  |  |  |  |
| 131674 | Date K80 first reported (cholelithiasis) |  |  |  |  |
| 131702 | Date L03 first reported (cellulitis) |  |  |  |  |
| 131708 | Date L08 first reported (other local infections of skin and subcutaneous tissue) |  |  |  |  |
| 131720 | Date L20 first reported (atopic dermatitis) |  |  |  |  |
| 131740 | Date L30 first reported (other dermatitis) |  |  |  |  |
| 131742 | Date L40 first reported (psoriasis) |  |  |  |  |
| 131768 | Date L57 first reported (skin changes due to chronic exposure to nonionising radiation) |  |  |  |  |
| 131794 | Date L72 first reported (follicular cysts of skin and subcutaneous tissue) |  |  |  |  |
| 131806 | Date L82 first reported (seborrhoeic keratosis) |  |  |  |  |
| 131836 | Date L98 first reported (other disorders of skin and subcutaneous tissue, not elsewhere classified) |  |  |  |  |
| 131858 | Date M10 first reported (gout) |  |  |  |  |
| 131864 | Date M13 first reported (other arthritis) |  |  |  |  |
| 131872 | Date M17 first reported (gonarthrosis [arthrosis of knee]) |  |  |  |  |
| 131876 | Date M19 first reported (other arthrosis) |  |  |  |  |
| 131878 | Date M20 first reported (acquired deformities of fingers and toes) |  |  |  |  |
| 131884 | Date M23 first reported (internal derangement of knee) |  |  |  |  |
| 131888 | Date M25 first reported (other joint disorders, not elsewhere classified) |  |  |  |  |
| 131916 | Date M47 first reported (spondylosis) |  |  |  |  |
| 131924 | Date M51 first reported (other intervertebral disk disorders) |  |  |  |  |
| 131928 | Date M54 first reported (dorsalgia) |  |  |  |  |
| 131938 | Date M65 first reported (synovitis and tenosynovitis) |  |  |  |  |
| 131946 | Date M70 first reported (soft tissue disorders related to use, overuse and pressure) |  |  |  |  |
| 131950 | Date M72 first reported (fibroblastic disorders) |  |  |  |  |
| 131954 | Date M75 first reported (shoulder lesions) |  |  |  |  |
| 131958 | Date M77 first reported (other enthesopathies) |  |  |  |  |
| 131960 | Date M79 first reported (other soft tissue disorders, not elsewhere classified) |  |  |  |  |
| 131964 | Date M81 first reported (osteoporosis without pathological fracture) |  |  |  |  |
| 132054 | Date N30 first reported (cystitis) |  |  |  |  |
| 132070 | Date N39 first reported (other disorders of urinary system) |  |  |  |  |
| 132072 | Date N40 first reported (hyperplasia of prostate) |  |  |  |  |
| 132096 | Date N60 first reported (benign mammary dysplasia) |  |  |  |  |
| 132102 | Date N63 first reported (unspecified lump in breast) |  |  |  |  |
| 132104 | Date N64 first reported (other disorders of breast) |  |  |  |  |
| 132124 | Date N81 first reported (female genital prolapse) |  |  |  |  |
| 132128 | Date N83 first reported (noninflammatory disorders of ovary, fallopian tube and broad ligament) |  |  |  |  |
| 132130 | Date N84 first reported (polyp of female genital tract) |  |  |  |  |
| 132140 | Date N89 first reported (other noninflammatory disorders of vagina) |  |  |  |  |
| 132146 | Date N92 first reported (excessive, frequent and irregular menstruation) |  |  |  |  |
| 132148 | Date N93 first reported (other abnormal uterine and vaginal bleeding) |  |  |  |  |
| 132150 | Date N94 first reported (pain and other conditions associated with female genital organs and menstrual cycle) |  |  |  | |
| 132152 | Date N95 first reported (menopausal and other perimenopausal disorders) |  |  |  |  |
| 132276 | Date O80 first reported (single spontaneous delivery) |  |  |  |  |
| Derived^[7]^ | TBI (traumatic brain injury) |  |  |  | Based on ICD10 (S00-S09, T90), details in ref^[7]^ |
| Derived^[5]^ | Diabetes |  |  |  | Based on FieldID (130706, 130708, 130710, 130712, 130714), details in ref^[5]^ |
| Derived^[5]^ | CHD (coronary heart Medical history) |  |  |  | Based on FieldID (131296, 131298, 131300, 131302, 131304, 131306), details in ref^[5]^ |
| Derived^[5]^ | stroke |  |  |  | Based on FieldID (131360, 131362, 131364, 131366, 131368), details in ref^[5]^ |
| Derived^[5]^ | COPD |  |  |  | Based on FieldID (131486, 131488, 131490, 131492), details in ref^[5]^ |
| Derived | Arthritis |  |  |  | Based on FieldID (131868, 131870, 131872, 131874, 131876), details in ref^[5]^ |
| Derived | Hypertension |  |  |  | Based on FieldID (131286, 131288, 131290, 131292, 131294), details in ref^[8]^ |
| Derived | Hearing_loss |  |  |  | Based on FieldID (131258, 131260), details in ref^[4]^ |
| Derived | Vision_impairment |  |  |  | Based on FieldID (131212), details in ref^[6]^ |
| **Mental Health** | | | | | |
| 130854 | Date F10 first reported (mental and behavioural disorders due to use of alcohol) |  |  | |  |
| 130856 | Date F11 first reported (mental and behavioural disorders due to use of opioids) |  |  | |  |
| 130858 | Date F12 first reported (mental and behavioural disorders due to use of cannabinoids) |  |  | |  |
| 130860 | Date F13 first reported (mental and behavioural disorders due to use of sedatives or hypnotics) |  |  | |  |
| 130868 | Date F17 first reported (mental and behavioural disorders due to use of tobacco) |  |  | |  |
| 130872 | Date F19 first reported (mental and behavioural disorders due to multiple drug use and use of other psychoactive substances) |  |  | |  |
| 130890 | Date F30 first reported (manic episode) |  |  | |  |
| 130892 | Date F31 first reported (bipolar affective disorder) |  |  | |  |
| 130894 | Date F32 first reported (depressive episode) |  |  | |  |
| 130896 | Date F33 first reported (recurrent depressive disorder) |  |  | |  |
| 130898 | Date F34 first reported (persistent mood [affective] disorders) |  |  | |  |
| 130904 | Date F40 first reported (phobic anxiety disorders) |  |  | |  |
| 130906 | Date F41 first reported (other anxiety disorders) |  |  | |  |
| 130908 | Date F42 first reported (obsessive-compulsive disorder) |  |  | |  |
| 130910 | Date F43 first reported (reaction to severe stress, and adjustment disorders) |  |  | |  |
| 130912 | Date F44 first reported (dissociative [conversion] disorders) |  |  | |  |
| 130914 | Date F45 first reported (somatoform disorders) |  |  | |  |
| 130916 | Date F48 first reported (other neurotic disorders) |  |  | |  |
| 130918 | Date F50 first reported (eating disorders) |  |  | |  |
| 130920 | Date F51 first reported (nonorganic sleep disorders) |  |  | |  |
| 130922 | Date F52 first reported (sexual dysfunction, not caused by organic disorder or disease) |  |  | |  |
| 130924 | Date F53 first reported (mental and behavioural disorders associated with the puerperium, not elsewhere classified) |  |  | |  |
| 130932 | Date F60 first reported (specific personality disorders) |  |  | |  |
| 130944 | Date F66 first reported (psychological and behavioural disorders associated with sexual development and orientation) |  |  | |  |
| 130990 | Date F99 first reported (mental disorder, not otherwise specified) |  |  | |  |
| Derived | Dementia |  |  | |  |
| Derived | Intellectual disability |  |  | |  |
| Derived | Other personality disorders |  |  | |  |
| Derived | Other behavioural syndromes |  |  | |  |
| Derived | Other substance abuse |  |  | |  |
| Derived | Others Mood disorders |  |  | |  |
| Derived | Other organic mental disorders |  |  | |  |
| Derived | Psychological development disorders |  |  | |  |
| 1920 | Mood swings | 100349 | set -1 -3 as NA | |  |
| 1930 | Miserableness | 100349 | set -1 -3 as NA | |  |
| 1940 | Irritability | 100349 | set -1 -3 as NA | |  |
| 1950 | Sensitivity / hurt feelings | 100349 | set -1 -3 as NA | |  |
| 1960 | Fed-up feelings | 100349 | set -1 -3 as NA | |  |
| 1970 | Nervous feelings | 100349 | set -1 -3 as NA | |  |
| 1980 | Worrier / anxious feelings | 100349 | set -1 -3 as NA | |  |
| 1990 | Tense / 'highly strung' | 100349 | set -1 -3 as NA | |  |
| 2000 | Worry too long after embarrassment | 100349 | set -1 -3 as NA | |  |
| 2010 | Suffer from 'nerves' | 100349 | set -1 -3 as NA | |  |
| 2020 | Loneliness, isolation | 100349 | set -1 -3 as NA | |  |
| 2030 | Guilty feelings | 100349 | set -1 -3 as NA | |  |
| 2090 | seen doctor gp for nerves anxiety tension or depression | 100349 | set -1 -3 as NA | |  |
| 2100 | seen a psychiatrist for nerves anxiety tension or depression | 100349 | set -1 -3 as NA | |  |
| 2110 | Able to confide | 100501 | -1 | Do not know | set as NA |
|  |  |  | -3 | Prefer not to answer | set as NA |
|  |  |  | 0 | Never or almost never | set as 1 |
|  |  |  | 1 | Once every few months | set as 1 |
|  |  |  | 2 | About once a month | set as 1 |
|  |  |  | 3 | About once a week | set as 0 |
|  |  |  | 4 | 2-4 times a week | set as 0 |
|  |  |  | 5 | Almost daily | set as 0 |
| 2050 | Frequency of depressed mood in last 2 weeks | 100484 | -1 | Do not know | set as NA |
|  |  |  | -3 | Prefer not to answer | set as NA |
|  |  |  | 1 | Not at all | set as 0 |
|  |  |  | 2 | Several days | set as 1 |
|  |  |  | 3 | More than half the days | set as 1 |
|  |  |  | 4 | Nearly every day | set as 1 |
| 2070 | frequency of tenseness restlessness in last 2 week | 100484 | same as above | | |
| 2080 | frequency of tiredness lethargy in last 2 weeks | 100484 | same as above | | |
| 2060 | frequency of tiredness lethargy in last 2 weeks | 100484 | same as above | | |
| 6145 | Illness, injury, bereavement, stress in last 2 years | 100502 | -3 | Prefer not to answer | set as NA |
|  |  |  | -7 | None of the above | set as 0 |
|  |  |  | 1 | Serious illness, injury or assault to yourself | set as 1 |
|  |  |  | 2 | Serious illness, injury or assault of a close relative | set as 1 |
|  |  |  | 3 | Death of a close relative | set as 1 |
|  |  |  | 4 | Death of a spouse or partner | set as 1 |
|  |  |  | 5 | Marital separation/divorce | set as 1 |
|  |  |  | 6 | Financial difficulties | set as 1 |
|  | | | | | |

| **Supplementary Table 3. Multiple-choice categorical variables and processing.** FieldID, the index number of each variable; Field, the detail meaning of each variable; Data-coding, the index number of each variable’s coding; Encoding, the coding of each class of variable; Label, the meaning of each class of variable; Coding notes, the recoding of the variable; SES: socioeconomic status. | | | | | | |
| --- | --- | --- | --- | --- | --- | --- |
| **FieldID** | **Field** | **Data-coding** | **Encoding** | **Label** | **coding notes** | |
| **SES** | | | | | | |
| 6142 | Current employment status | 100295 | -3 | Prefer not to answer | set as NA |  |
|  |  |  | -7 | None of the above | set as NA |  |
|  |  |  | 1 | In paid employment or self-employed | set as 0 | Turn data into binary classification: 1 unemployed; 0: employed |
|  |  |  | 2 | Retired | set as 0 |  |
|  |  |  | 3 | Looking after home and/or family | set as 1 |  |
|  |  |  | 4 | Unable to work because of sickness or disability | set as 1 |  |
|  |  |  | 5 | Unemployed | set as 1 |  |
|  |  |  | 6 | Doing unpaid or voluntary work | set as 0 |  |
|  |  |  | 7 | Full or part-time student | set as 0 |  |
| 6138 | Qualifications | 100305 | 1 | College or University degree | set as 0 |  |
|  |  |  | 2 | A levels/AS levels or equivalent | set as 1 | Turn data into binary classification：1: lower education; 0: higher education |
|  |  |  | 3 | O levels/GCSEs or equivalent | set as 1 |  |
|  |  |  | 4 | CSEs or equivalent | set as 1 |  |
|  |  |  | 5 | NVQ or HND or HNC or equivalent | set as 1 |  |
|  |  |  | 6 | Other professional qualifications eg: nursing, teaching | set as 0 |  |
|  |  |  | -1 | None of the above | set as 1 | Regarded as below high school diploma |
|  |  |  | -3 | Prefer not to answer | set as NA |  |
| **Medical history** | | | | | | |
| 6146 | Attendance/disability/mobility allowance | 100510 | -1 | Do not know | set as NA | Turn data into binary classification: 1: disability; 0: no disability |
|  |  |  | -3 | Prefer not to answer | set as NA |  |
|  |  |  | -7 | None of the above | set as 0 |  |
|  |  |  | 1 | Attendance allowance (means severely disabled) | set as 1 |  |
|  |  |  | 2 | Disability living allowance | set as 1 |  |
|  |  |  | 3 | Blue badge (Disabilities or health conditions cause immobility) | set as 1 |  |
| 6149 | Mouth/teeth dental problems | 100538 | -3 | Prefer not to answer | set as NA |  |
|  |  |  | -7 | None of the above |  |  |
|  |  |  | 1 | Mouth ulcers |  |  |
|  |  |  | 2 | Painful gums |  |  |
|  |  |  | 3 | Bleeding gums |  |  |
|  |  |  | 4 | Loose teeth |  |  |
|  |  |  | 5 | Toothache |  |  |
|  |  |  | 6 | Dentures |  |  |
| **Lifestyles** |  |  | 1 |  |  |  |
| 6160 | Leisure/social activities | 100328 | -3 | Prefer not to answer | set as NA |  |
|  |  |  | -7 | None of the above |  |  |
|  |  |  | 1 | Sports club or gym |  |  |
|  |  |  | 2 | Pub or social club |  |  |
|  |  |  | 3 | Religious group |  |  |
|  |  |  | 4 | Adult education class |  |  |
|  |  |  | 5 | Other group activity |  |  |
| 1031 | Frequency of friend/family visits | 100327 | -1 | Do not know | set as NA |  |
|  |  |  | -3 | Prefer not to answer | set as NA |  |
|  |  |  | 1 | Almost daily | set as 1 |  |
|  |  |  | 2 | 2-4 times a week | set as 1 |  |
|  |  |  | 3 | About once a week | set as 1 |  |
|  |  |  | 4 | About once a month | set as 1 |  |
|  |  |  | 5 | Once every few months | set as 1 |  |
|  |  |  | 6 | Never or almost never | set as 0 |  |
|  |  |  | 7 | No friends/family outside household | set as 0 |  |
| 6155 | Vitamin and mineral supplements | 100629 | -3 | Prefer not to answer | set as NA |  |
|  |  |  | -7 | None of the above | set as 0 |  |
|  |  |  | 1 | Vitamin A |  |  |
|  |  |  | 2 | Vitamin B |  |  |
|  |  |  | 3 | Vitamin C |  |  |
|  |  |  | 4 | Vitamin D |  |  |
|  |  |  | 5 | Vitamin E |  |  |
|  |  |  | 6 | Folic acid or Folate (Vit B9) |  |  |
|  |  |  | 7 | Multivitamins +/- minerals |  |  |
| 6179 | Mineral and other dietary supplements | 100630 | -3 | Prefer not to answer | set as NA |  |
|  |  |  | -7 | None of the above |  |  |
|  |  |  | 1 | Fish oil (including cod liver oil) |  |  |
|  |  |  | 2 | Glucosamine |  |  |
|  |  |  | 3 | Calcium |  |  |
|  |  |  | 4 | Zinc |  |  |
|  |  |  | 5 | Iron |  |  |
|  |  |  | 6 | Selenium |  |  |
|  | | | | | | |

| **Supplementary Table 4. Continuous variables and processing.** FieldID, the index number of each variable; Field, the detail meaning of each variable; Data-coding, the index number of each variable’s coding; Encoding, the coding of each class of variable; Label, the meaning of each class of variable; Coding notes, the recoding of the variable, Derived, derived variables. | | | | | | | |
| --- | --- | --- | --- | --- | --- | --- | --- |
| **FieldID** | **Field** | **Data-coding** | | **Encoding** | **Label** | **coding notes** | **Derived** |
| **SES** |  | | | | | | |
| 189 | Townsend deprivation index at recruitment |  |  | |  |  |  |
| **Physical measures** | | | | | | | |
| 23099 | Body fat percentage |  |  | |  |  |  |
| 23105 | Basal metabolic rate |  |  | |  |  |  |
| 23111 | Leg fat percentage (right) |  |  | |  | mean value of left and right | Leg_fat_percentage |
| 23115 | Leg fat percentage (left) |  |  | |  |  |  |
| 23119 | Arm fat percentage (right) |  |  | |  | mean value of left and right | arm_fat_percentage |
| 23123 | Arm fat percentage (left) |  |  | |  |  |  |
| 23127 | Trunk fat percentage |  |  | |  |  |  |
| 3062 | Forced vital capacity (FVC) |  |  | |  | mean value of 3 arrays | mean value of z-scores of 3 fields= Lung_function |
| 3063 | Forced expiratory volume in 1-second (FEV1) | |  | |  |  |  |
| 3064 | Peak expiratory flow (PEF) |  |  | |  |  |  |
| 4079 | Diastolic blood pressure, automated reading |  |  | |  | mean value of 2 arrays |  |
| 4080 | Systolic blood pressure, automated reading |  |  | |  |  |  |
| 46 | Hand grip strength (left) |  |  | |  | mean value of left and right | hand_grip_strength |
| 47 | Hand grip strength (right) |  |  | |  |  |  |
| **Lifestyles** | | | | | | | |
| 1050 | Time spend outdoors in summer | 100329 | -1 | | Do not know | set as NA |  |
|  |  |  | - 10 | | Less than an hour a day | set as 0.5 |  |
|  |  |  | -3 | | Prefer not to answer | set as NA |  |
|  |  |  |  | |  |  |  |
| 1060 | Time spent outdoors in winter | 100329 |  | |  |  |  |
| 2139 | Age first had sexual intercourse | 100504 | -1 | | Do not know | set as NA |  |
|  |  |  | -2 | | Never had sex | set as NA |  |
|  |  |  | -3 | | Prefer not to answer | set as NA |  |
| 2149 | Lifetime number of sexual partners | 100291 |  | |  | set -1 -3 as NA |  |
| 22037 | MET minutes per week for walking |  |  | |  |  |  |
| 22038 | MET minutes per week for moderate activity |  |  | |  |  |  |
| 22039 | MET minutes per week for vigorous activity |  |  | |  |  |  |
| 22040 | Summed MET minutes per week for all activity | |  | |  |  |  |
| 1070 | Time spent watching television (TV) | 100329 | -1 | | Do not know | set as NA |  |
|  |  |  | - 10 | | Less than an hour a day | set as 0.5 |  |
|  |  |  | -3 | | Prefer not to answer | set as NA |  |
| 1080 | Time spent using computer | 100329 |  | |  |  |  |
| 1488 | Tea intake | 100373 | -1 | | Do not know | set as NA |  |
|  |  |  | - 10 | | Less than one | set as 0.5 |  |
|  |  |  | -3 | | Prefer not to answer | set as NA |  |
| 1498 | Coffee intake | 100373 |  | |  |  |  |
| 1528 | Water intake | 100373 |  | |  |  |  |
| Derived | Healthy_diet_level |  |  | |  |  |  |
| Derived | Vegetable intake |  |  | |  |  |  |
| **Local environment** | |  |  | |  |  |  |
| 24003 | Nitrogen dioxide air pollution; 2010 |  |  | |  | Mean values |  |
| 24004 | Nitrogen oxides air pollution; 2010 |  |  | |  |  |  |
| 24016 | Nitrogen dioxide air pollution; 2005 |  |  | |  |  |  |
| 24017 | Nitrogen dioxide air pollution; 2006 |  |  | |  |  |  |
| 24018 | Nitrogen dioxide air pollution; 2007 |  |  | |  |  |  |
| 24005 | Particulate matter air pollution (pm10); 2010 |  |  | |  | Mean values |  |
| 24019 | Particulate matter air pollution (pm10); 2007 |  |  | |  |  |  |
| 24006 | Particulate matter air pollution (pm2.5); 2010 |  |  | |  |  |  |
| 24008 | Particulate matter air pollution 2.5-10um; 2010 |  |  | |  |  |  |
| 24020 | Average daytime sound level of noise pollution |  |  | |  |  |  |
| 24021 | Average evening sound level of noise pollution |  |  | |  |  |  |
| 24022 | Average night-time sound level of noise pollution |  |  | |  |  |  |
| 24023 | Average 16-hour sound level of noise pollution |  |  | |  |  |  |
| 24024 | Average 24-hour sound level of noise pollution |  |  | |  |  |  |
| 24500 | Greenspace percentage, buffer 1000m |  |  | |  |  |  |
| 24501 | Domestic garden percentage, buffer 1000m |  |  | |  |  |  |
| 24502 | Water percentage, buffer 1000m |  |  | |  |  |  |
| 24503 | Greenspace percentage, buffer 300m |  |  | |  |  |  |
| 24504 | Domestic garden percentage, buffer 300m |  |  | |  |  |  |
| 24505 | Water percentage, buffer 300m |  |  | |  |  |  |
| 24506 | Natural environment percentage, buffer 1000m |  |  | |  |  |  |
| 24507 | Natural environment percentage, buffer 300m |  |  | |  |  |  |
|  | | | | | | | |

| **Supplementary Table 5. The missing values for exposure factors** | | | |
| --- | --- | --- | --- |
| Variables | Late-onset schizophrenia | | Total  (N=482708) |
|  | No (N=481432) | Yes (N=1276) |  |
| X738 |  |  |  |
| Less than 18,000 | 92865 (19.3%) | 526 (41.2%) | 93391 (19.3%) |
| 18,000 to 30,999 | 104864 (21.8%) | 227 (17.8%) | 105091 (21.8%) |
| 31,000 to 51,999 | 107649 (22.4%) | 148 (11.6%) | 107797 (22.3%) |
| 52,000 to 100,000 | 83974 (17.4%) | 75 (5.9%) | 84049 (17.4%) |
| Greader than 100,000 | 22299 (4.6%) | 20 (1.6%) | 22319 (4.6%) |
| Missing | 69781 (14.5%) | 280 (21.9%) | 70061 (14.5%) |
| X1120 |  |  |  |
| Less than 5 mins | 85625 (17.8%) | 224 (17.6%) | 85849 (17.8%) |
| 5-29 mins | 156928 (32.6%) | 340 (26.6%) | 157268 (32.6%) |
| 30-59 mins | 69193 (14.4%) | 148 (11.6%) | 69341 (14.4%) |
| 1-3 hours | 57251 (11.9%) | 135 (10.6%) | 57386 (11.9%) |
| 4-6 hours | 16739 (3.5%) | 36 (2.8%) | 16775 (3.5%) |
| More than 6 hours | 17359 (3.6%) | 36 (2.8%) | 17395 (3.6%) |
| Missing | 78337 (16.3%) | 357 (28.0%) | 78694 (16.3%) |
| X1130 |  |  |  |
| Never or almost never | 333186 (69.2%) | 811 (63.6%) | 333997 (69.2%) |
| Less than half the time | 37807 (7.9%) | 62 (4.9%) | 37869 (7.8%) |
| About half the time | 14660 (3.0%) | 13 (1.0%) | 14673 (3.0%) |
| More than half the time | 8253 (1.7%) | 14 (1.1%) | 8267 (1.7%) |
| Always or almost always | 10907 (2.3%) | 22 (1.7%) | 10929 (2.3%) |
| Missing | 76619 (15.9%) | 354 (27.7%) | 76973 (15.9%) |
| X1160 |  |  |  |
| 0 | 351846 (73.1%) | 768 (60.2%) | 352614 (73.0%) |
| 1 | 126633 (26.3%) | 480 (37.6%) | 127113 (26.3%) |
| Missing | 2953 (0.6%) | 28 (2.2%) | 2981 (0.6%) |
| X1190 |  |  |  |
| Never/rarely | 270433 (56.2%) | 549 (43.0%) | 270982 (56.1%) |
| Sometimes | 184649 (38.4%) | 588 (46.1%) | 185237 (38.4%) |
| Usually | 25568 (5.3%) | 130 (10.2%) | 25698 (5.3%) |
| Missing | 782 (0.2%) | 9 (0.7%) | 791 (0.2%) |
| X1200 |  |  |  |
| Never/rarely | 116132 (24.1%) | 261 (20.5%) | 116393 (24.1%) |
| Sometimes | 229327 (47.6%) | 578 (45.3%) | 229905 (47.6%) |
| Usually | 135547 (28.2%) | 433 (33.9%) | 135980 (28.2%) |
| Missing | 426 (0.1%) | 4 (0.3%) | 430 (0.1%) |
| X1210 |  |  |  |
| 1 | 166908 (34.7%) | 351 (27.5%) | 167259 (34.7%) |
| 2 | 280322 (58.2%) | 750 (58.8%) | 281072 (58.2%) |
| Missing | 34202 (7.1%) | 175 (13.7%) | 34377 (7.1%) |
| X1220 |  |  |  |
| Never/rarely | 364149 (75.6%) | 824 (64.6%) | 364973 (75.6%) |
| Sometimes | 101456 (21.1%) | 345 (27.0%) | 101801 (21.1%) |
| Often | 13322 (2.8%) | 75 (5.9%) | 13397 (2.8%) |
| All of the time | 36 (0.0%) | 1 (0.1%) | 37 (0.0%) |
| Missing | 2469 (0.5%) | 31 (2.4%) | 2500 (0.5%) |
| X1269 |  |  |  |
| 0 | 409317 (85.0%) | 900 (70.5%) | 410217 (85.0%) |
| 1 | 24353 (5.1%) | 95 (7.4%) | 24448 (5.1%) |
| Missing | 47762 (9.9%) | 281 (22.0%) | 48043 (10.0%) |
| X1279 |  |  |  |
| 0 | 327013 (67.9%) | 675 (52.9%) | 327688 (67.9%) |
| 1 | 78375 (16.3%) | 246 (19.3%) | 78621 (16.3%) |
| Missing | 76044 (15.8%) | 355 (27.8%) | 76399 (15.8%) |
| X1920 |  |  |  |
| 0 | 256003 (53.2%) | 482 (37.8%) | 256485 (53.1%) |
| 1 | 212833 (44.2%) | 743 (58.2%) | 213576 (44.2%) |
| Missing | 12596 (2.6%) | 51 (4.0%) | 12647 (2.6%) |
| X1930 |  |  |  |
| 0 | 270905 (56.3%) | 589 (46.2%) | 271494 (56.2%) |
| 1 | 201676 (41.9%) | 641 (50.2%) | 202317 (41.9%) |
| Missing | 8851 (1.8%) | 46 (3.6%) | 8897 (1.8%) |
| X1940 |  |  |  |
| 0 | 330611 (68.7%) | 806 (63.2%) | 331417 (68.7%) |
| 1 | 128466 (26.7%) | 369 (28.9%) | 128835 (26.7%) |
| Missing | 22355 (4.6%) | 101 (7.9%) | 22456 (4.7%) |
| X1950 |  |  |  |
| 0 | 208346 (43.3%) | 452 (35.4%) | 208798 (43.3%) |
| 1 | 258563 (53.7%) | 763 (59.8%) | 259326 (53.7%) |
| Missing | 14523 (3.0%) | 61 (4.8%) | 14584 (3.0%) |
| X1960 |  |  |  |
| 0 | 280004 (58.2%) | 592 (46.4%) | 280596 (58.1%) |
| 1 | 190518 (39.6%) | 637 (49.9%) | 191155 (39.6%) |
| Missing | 10910 (2.3%) | 47 (3.7%) | 10957 (2.3%) |
| X1970 |  |  |  |
| 0 | 358560 (74.5%) | 689 (54.0%) | 359249 (74.4%) |
| 1 | 109697 (22.8%) | 532 (41.7%) | 110229 (22.8%) |
| Missing | 13175 (2.7%) | 55 (4.3%) | 13230 (2.7%) |
| X1980 |  |  |  |
| 0 | 204222 (42.4%) | 371 (29.1%) | 204593 (42.4%) |
| 1 | 264043 (54.8%) | 857 (67.2%) | 264900 (54.9%) |
| Missing | 13167 (2.7%) | 48 (3.8%) | 13215 (2.7%) |
| X1990 |  |  |  |
| 0 | 382148 (79.4%) | 769 (60.3%) | 382917 (79.3%) |
| 1 | 81519 (16.9%) | 416 (32.6%) | 81935 (17.0%) |
| Missing | 17765 (3.7%) | 91 (7.1%) | 17856 (3.7%) |
| X2000 |  |  |  |
| 0 | 241464 (50.2%) | 548 (42.9%) | 242012 (50.1%) |
| 1 | 219700 (45.6%) | 650 (50.9%) | 220350 (45.6%) |
| Missing | 20268 (4.2%) | 78 (6.1%) | 20346 (4.2%) |
| X2010 |  |  |  |
| 0 | 365884 (76.0%) | 688 (53.9%) | 366572 (75.9%) |
| 1 | 96905 (20.1%) | 507 (39.7%) | 97412 (20.2%) |
| Missing | 18643 (3.9%) | 81 (6.3%) | 18724 (3.9%) |
| X2020 |  |  |  |
| 0 | 386306 (80.2%) | 776 (60.8%) | 387082 (80.2%) |
| 1 | 86802 (18.0%) | 456 (35.7%) | 87258 (18.1%) |
| Missing | 8324 (1.7%) | 44 (3.4%) | 8368 (1.7%) |
| X2030 |  |  |  |
| 0 | 333281 (69.2%) | 753 (59.0%) | 334034 (69.2%) |
| 1 | 134542 (27.9%) | 465 (36.4%) | 135007 (28.0%) |
| Missing | 13609 (2.8%) | 58 (4.5%) | 13667 (2.8%) |
| X2110 |  |  |  |
| 0 | 344767 (71.6%) | 800 (62.7%) | 345567 (71.6%) |
| 1 | 119705 (24.9%) | 402 (31.5%) | 120107 (24.9%) |
| Missing | 16960 (3.5%) | 74 (5.8%) | 17034 (3.5%) |
| X2159 |  |  |  |
| 0 | 416718 (86.6%) | 952 (74.6%) | 417670 (86.5%) |
| 1 | 15005 (3.1%) | 58 (4.5%) | 15063 (3.1%) |
| Missing | 49709 (10.3%) | 266 (20.8%) | 49975 (10.4%) |
| X22036 |  |  |  |
| 0 | 304879 (63.3%) | 699 (54.8%) | 305578 (63.3%) |
| 1 | 67937 (14.1%) | 195 (15.3%) | 68132 (14.1%) |
| Missing | 108616 (22.6%) | 382 (29.9%) | 108998 (22.6%) |
| X6142 |  |  |  |
| 0 | 466086 (96.8%) | 1197 (93.8%) | 467283 (96.8%) |
| 1 | 15346 (3.2%) | 79 (6.2%) | 15425 (3.2%) |
| X6138 |  |  |  |
| 0 | 226067 (47.0%) | 475 (37.2%) | 226542 (46.9%) |
| 1 | 249811 (51.9%) | 779 (61.1%) | 250590 (51.9%) |
| Missing | 5554 (1.2%) | 22 (1.7%) | 5576 (1.2%) |
| X6146 |  |  |  |
| 0 | 453932 (94.3%) | 947 (74.2%) | 454879 (94.2%) |
| 1 | 27500 (5.7%) | 329 (25.8%) | 27829 (5.8%) |
| X6149_0 |  |  |  |
| 0 | 192349 (40.0%) | 676 (53.0%) | 193025 (40.0%) |
| 1 | 289083 (60.0%) | 600 (47.0%) | 289683 (60.0%) |
| X6149_1 |  |  |  |
| 0 | 432897 (89.9%) | 1136 (89.0%) | 434033 (89.9%) |
| 1 | 48535 (10.1%) | 140 (11.0%) | 48675 (10.1%) |
| X6149_2 |  |  |  |
| 0 | 466898 (97.0%) | 1189 (93.2%) | 468087 (97.0%) |
| 1 | 14534 (3.0%) | 87 (6.8%) | 14621 (3.0%) |
| X6149_3 |  |  |  |
| 0 | 417526 (86.7%) | 1075 (84.2%) | 418601 (86.7%) |
| 1 | 63906 (13.3%) | 201 (15.8%) | 64107 (13.3%) |
| X6149_4 |  |  |  |
| 0 | 460698 (95.7%) | 1179 (92.4%) | 461877 (95.7%) |
| 1 | 20734 (4.3%) | 97 (7.6%) | 20831 (4.3%) |
| X6149_5 |  |  |  |
| 0 | 460167 (95.6%) | 1163 (91.1%) | 461330 (95.6%) |
| 1 | 21265 (4.4%) | 113 (8.9%) | 21378 (4.4%) |
| X6149_6 |  |  |  |
| 0 | 401327 (83.4%) | 956 (74.9%) | 402283 (83.3%) |
| 1 | 80105 (16.6%) | 320 (25.1%) | 80425 (16.7%) |
| X6155_0 |  |  |  |
| 0 | 155143 (32.2%) | 490 (38.4%) | 155633 (32.2%) |
| 1 | 326289 (67.8%) | 786 (61.6%) | 327075 (67.8%) |
| X6155_1 |  |  |  |
| 0 | 471858 (98.0%) | 1227 (96.2%) | 473085 (98.0%) |
| 1 | 9574 (2.0%) | 49 (3.8%) | 9623 (2.0%) |
| X6155_2 |  |  |  |
| 0 | 460865 (95.7%) | 1179 (92.4%) | 462044 (95.7%) |
| 1 | 20567 (4.3%) | 97 (7.6%) | 20664 (4.3%) |
| X6155_3 |  |  |  |
| 0 | 439504 (91.3%) | 1137 (89.1%) | 440641 (91.3%) |
| 1 | 41928 (8.7%) | 139 (10.9%) | 42067 (8.7%) |
| X6155_4 |  |  |  |
| 0 | 462271 (96.0%) | 1199 (94.0%) | 463470 (96.0%) |
| 1 | 19161 (4.0%) | 77 (6.0%) | 19238 (4.0%) |
| X6155_5 |  |  |  |
| 0 | 466895 (97.0%) | 1221 (95.7%) | 468116 (97.0%) |
| 1 | 14537 (3.0%) | 55 (4.3%) | 14592 (3.0%) |
| X6155_6 |  |  |  |
| 0 | 470592 (97.7%) | 1229 (96.3%) | 471821 (97.7%) |
| 1 | 10840 (2.3%) | 47 (3.7%) | 10887 (2.3%) |
| X6155_7 |  |  |  |
| 0 | 376407 (78.2%) | 964 (75.5%) | 377371 (78.2%) |
| 1 | 105025 (21.8%) | 312 (24.5%) | 105337 (21.8%) |
| X6179_0 |  |  |  |
| 0 | 208532 (43.3%) | 571 (44.7%) | 209103 (43.3%) |
| 1 | 272900 (56.7%) | 705 (55.3%) | 273605 (56.7%) |
| X6179_1 |  |  |  |
| 0 | 330534 (68.7%) | 878 (68.8%) | 331412 (68.7%) |
| 1 | 150898 (31.3%) | 398 (31.2%) | 151296 (31.3%) |
| X6179_2 |  |  |  |
| 0 | 389529 (80.9%) | 1102 (86.4%) | 390631 (80.9%) |
| 1 | 91903 (19.1%) | 174 (13.6%) | 92077 (19.1%) |
| X6179_3 |  |  |  |
| 0 | 447941 (93.0%) | 1148 (90.0%) | 449089 (93.0%) |
| 1 | 33491 (7.0%) | 128 (10.0%) | 33619 (7.0%) |
| X6179_4 |  |  |  |
| 0 | 461668 (95.9%) | 1210 (94.8%) | 462878 (95.9%) |
| 1 | 19764 (4.1%) | 66 (5.2%) | 19830 (4.1%) |
| X6179_5 |  |  |  |
| 0 | 465555 (96.7%) | 1194 (93.6%) | 466749 (96.7%) |
| 1 | 15877 (3.3%) | 82 (6.4%) | 15959 (3.3%) |
| X6179_6 |  |  |  |
| 0 | 470028 (97.6%) | 1234 (96.7%) | 471262 (97.6%) |
| 1 | 11404 (2.4%) | 42 (3.3%) | 11446 (2.4%) |
| X6160_0 |  |  |  |
| 0 | 335622 (69.7%) | 853 (66.8%) | 336475 (69.7%) |
| 1 | 145810 (30.3%) | 423 (33.2%) | 146233 (30.3%) |
| X6160_1 |  |  |  |
| 0 | 338482 (70.3%) | 1022 (80.1%) | 339504 (70.3%) |
| 1 | 142950 (29.7%) | 254 (19.9%) | 143204 (29.7%) |
| X6160_2 |  |  |  |
| 0 | 354509 (73.6%) | 943 (73.9%) | 355452 (73.6%) |
| 1 | 126923 (26.4%) | 333 (26.1%) | 127256 (26.4%) |
| X6160_3 |  |  |  |
| 0 | 406550 (84.4%) | 1046 (82.0%) | 407596 (84.4%) |
| 1 | 74882 (15.6%) | 230 (18.0%) | 75112 (15.6%) |
| X6160_4 |  |  |  |
| 0 | 446783 (92.8%) | 1144 (89.7%) | 447927 (92.8%) |
| 1 | 34649 (7.2%) | 132 (10.3%) | 34781 (7.2%) |
| X6160_5 |  |  |  |
| 0 | 377092 (78.3%) | 1004 (78.7%) | 378096 (78.3%) |
| 1 | 104340 (21.7%) | 272 (21.3%) | 104612 (21.7%) |
| Ever smoke |  |  |  |
| 0 | 192995 (40.1%) | 471 (36.9%) | 193466 (40.1%) |
| 1 | 249625 (51.9%) | 575 (45.1%) | 250200 (51.8%) |
| Missing | 38812 (8.1%) | 230 (18.0%) | 39042 (8.1%) |
| Alcohol intake |  |  |  |
| 0 | 213515 (44.4%) | 435 (34.1%) | 213950 (44.3%) |
| 1 | 197398 (41.0%) | 579 (45.4%) | 197977 (41.0%) |
| Missing | 70519 (14.6%) | 262 (20.5%) | 70781 (14.7%) |
| Fruit intake |  |  |  |
| 0 | 240965 (50.1%) | 633 (49.6%) | 241598 (50.1%) |
| 1 | 240467 (49.9%) | 643 (50.4%) | 241110 (49.9%) |
| Fish intake |  |  |  |
| 0 | 370092 (76.9%) | 939 (73.6%) | 371031 (76.9%) |
| 1 | 108782 (22.6%) | 317 (24.8%) | 109099 (22.6%) |
| Missing | 2558 (0.5%) | 20 (1.6%) | 2578 (0.5%) |
| Refined cereal intake |  |  |  |
| 0 | 387162 (80.4%) | 958 (75.1%) | 388120 (80.4%) |
| 1 | 55353 (11.5%) | 167 (13.1%) | 55520 (11.5%) |
| Missing | 38917 (8.1%) | 151 (11.8%) | 39068 (8.1%) |
| Grain intake |  |  |  |
| 0 | 288308 (59.9%) | 674 (52.8%) | 288982 (59.9%) |
| 1 | 130390 (27.1%) | 364 (28.5%) | 130754 (27.1%) |
| Missing | 62734 (13.0%) | 238 (18.7%) | 62972 (13.0%) |
| Red meat intake |  |  |  |
| 0 | 426163 (88.5%) | 1104 (86.5%) | 427267 (88.5%) |
| 1 | 52996 (11.0%) | 149 (11.7%) | 53145 (11.0%) |
| Missing | 2273 (0.5%) | 23 (1.8%) | 2296 (0.5%) |
| Process meat intake |  |  |  |
| 0 | 190917 (39.7%) | 448 (35.1%) | 191365 (39.6%) |
| 1 | 289419 (60.1%) | 815 (63.9%) | 290234 (60.1%) |
| Missing | 1096 (0.2%) | 13 (1.0%) | 1109 (0.2%) |
| X130176 |  |  |  |
| 0 | 456451 (94.8%) | 1255 (98.4%) | 457706 (94.8%) |
| 1 | 24981 (5.2%) | 21 (1.6%) | 25002 (5.2%) |
| X130178 |  |  |  |
| 0 | 472276 (98.1%) | 1264 (99.1%) | 473540 (98.1%) |
| 1 | 9156 (1.9%) | 12 (0.9%) | 9168 (1.9%) |
| X130188 |  |  |  |
| 0 | 470019 (97.6%) | 1252 (98.1%) | 471271 (97.6%) |
| 1 | 11413 (2.4%) | 24 (1.9%) | 11437 (2.4%) |
| X130226 |  |  |  |
| 0 | 461180 (95.8%) | 1221 (95.7%) | 462401 (95.8%) |
| 1 | 20252 (4.2%) | 55 (4.3%) | 20307 (4.2%) |
| X130622 |  |  |  |
| 0 | 470646 (97.8%) | 1240 (97.2%) | 471886 (97.8%) |
| 1 | 10786 (2.2%) | 36 (2.8%) | 10822 (2.2%) |
| X130648 |  |  |  |
| 0 | 472198 (98.1%) | 1235 (96.8%) | 473433 (98.1%) |
| 1 | 9234 (1.9%) | 41 (3.2%) | 9275 (1.9%) |
| X130696 |  |  |  |
| 0 | 456155 (94.7%) | 1180 (92.5%) | 457335 (94.7%) |
| 1 | 25277 (5.3%) | 96 (7.5%) | 25373 (5.3%) |
| X130792 |  |  |  |
| 0 | 468473 (97.3%) | 1235 (96.8%) | 469708 (97.3%) |
| 1 | 12959 (2.7%) | 41 (3.2%) | 13000 (2.7%) |
| X130814 |  |  |  |
| 0 | 408999 (85.0%) | 1027 (80.5%) | 410026 (84.9%) |
| 1 | 72433 (15.0%) | 249 (19.5%) | 72682 (15.1%) |
| X130868 |  |  |  |
| 0 | 460423 (95.6%) | 1187 (93.0%) | 461610 (95.6%) |
| 1 | 21009 (4.4%) | 89 (7.0%) | 21098 (4.4%) |
| X130906 |  |  |  |
| 0 | 464241 (96.4%) | 1126 (88.2%) | 465367 (96.4%) |
| 1 | 17191 (3.6%) | 150 (11.8%) | 17341 (3.6%) |
| X130910 |  |  |  |
| 0 | 471497 (97.9%) | 1230 (96.4%) | 472727 (97.9%) |
| 1 | 9935 (2.1%) | 46 (3.6%) | 9981 (2.1%) |
| X131052 |  |  |  |
| 0 | 460971 (95.8%) | 1228 (96.2%) | 462199 (95.8%) |
| 1 | 20461 (4.3%) | 48 (3.8%) | 20509 (4.2%) |
| X131060 |  |  |  |
| 0 | 472260 (98.1%) | 1236 (96.9%) | 473496 (98.1%) |
| 1 | 9172 (1.9%) | 40 (3.1%) | 9212 (1.9%) |
| X131074 |  |  |  |
| 0 | 468408 (97.3%) | 1245 (97.6%) | 469653 (97.3%) |
| 1 | 13024 (2.7%) | 31 (2.4%) | 13055 (2.7%) |
| X131128 |  |  |  |
| 0 | 473971 (98.5%) | 1259 (98.7%) | 475230 (98.5%) |
| 1 | 7461 (1.5%) | 17 (1.3%) | 7478 (1.5%) |
| X131142 |  |  |  |
| 0 | 464328 (96.4%) | 1226 (96.1%) | 465554 (96.4%) |
| 1 | 17104 (3.6%) | 50 (3.9%) | 17154 (3.6%) |
| X131166 |  |  |  |
| 0 | 469244 (97.5%) | 1227 (96.2%) | 470471 (97.5%) |
| 1 | 12188 (2.5%) | 49 (3.8%) | 12237 (2.5%) |
| X131222 |  |  |  |
| 0 | 468882 (97.4%) | 1245 (97.6%) | 470127 (97.4%) |
| 1 | 12550 (2.6%) | 31 (2.4%) | 12581 (2.6%) |
| X131224 |  |  |  |
| 0 | 465956 (96.8%) | 1243 (97.4%) | 467199 (96.8%) |
| 1 | 15476 (3.2%) | 33 (2.6%) | 15509 (3.2%) |
| X131262 |  |  |  |
| 0 | 471693 (98.0%) | 1250 (98.0%) | 472943 (98.0%) |
| 1 | 9739 (2.0%) | 26 (2.0%) | 9765 (2.0%) |
| X131264 |  |  |  |
| 0 | 469536 (97.5%) | 1240 (97.2%) | 470776 (97.5%) |
| 1 | 11896 (2.5%) | 36 (2.8%) | 11932 (2.5%) |
| X131350 |  |  |  |
| 0 | 473380 (98.3%) | 1249 (97.9%) | 474629 (98.3%) |
| 1 | 8052 (1.7%) | 27 (2.1%) | 8079 (1.7%) |
| X131396 |  |  |  |
| 0 | 469892 (97.6%) | 1226 (96.1%) | 471118 (97.6%) |
| 1 | 11540 (2.4%) | 50 (3.9%) | 11590 (2.4%) |
| X131402 |  |  |  |
| 0 | 466174 (96.8%) | 1238 (97.0%) | 467412 (96.8%) |
| 1 | 15258 (3.2%) | 38 (3.0%) | 15296 (3.2%) |
| X131404 |  |  |  |
| 0 | 467763 (97.2%) | 1226 (96.1%) | 468989 (97.2%) |
| 1 | 13669 (2.8%) | 50 (3.9%) | 13719 (2.8%) |
| X131426 |  |  |  |
| 0 | 463805 (96.3%) | 1236 (96.9%) | 465041 (96.3%) |
| 1 | 17627 (3.7%) | 40 (3.1%) | 17667 (3.7%) |
| X131428 |  |  |  |
| 0 | 464148 (96.4%) | 1240 (97.2%) | 465388 (96.4%) |
| 1 | 17284 (3.6%) | 36 (2.8%) | 17320 (3.6%) |
| X131430 |  |  |  |
| 0 | 463366 (96.2%) | 1249 (97.9%) | 464615 (96.3%) |
| 1 | 18066 (3.8%) | 27 (2.1%) | 18093 (3.7%) |
| X131436 |  |  |  |
| 0 | 450711 (93.6%) | 1187 (93.0%) | 451898 (93.6%) |
| 1 | 30721 (6.4%) | 89 (7.0%) | 30810 (6.4%) |
| X131442 |  |  |  |
| 0 | 473764 (98.4%) | 1262 (98.9%) | 475026 (98.4%) |
| 1 | 7668 (1.6%) | 14 (1.1%) | 7682 (1.6%) |
| X131456 |  |  |  |
| 0 | 470060 (97.6%) | 1234 (96.7%) | 471294 (97.6%) |
| 1 | 11372 (2.4%) | 42 (3.3%) | 11414 (2.4%) |
| X131462 |  |  |  |
| 0 | 451273 (93.7%) | 1175 (92.1%) | 452448 (93.7%) |
| 1 | 30159 (6.3%) | 101 (7.9%) | 30260 (6.3%) |
| X131464 |  |  |  |
| 0 | 438694 (91.1%) | 1174 (92.0%) | 439868 (91.1%) |
| 1 | 42738 (8.9%) | 102 (8.0%) | 42840 (8.9%) |
| X131468 |  |  |  |
| 0 | 472640 (98.2%) | 1258 (98.6%) | 473898 (98.2%) |
| 1 | 8792 (1.8%) | 18 (1.4%) | 8810 (1.8%) |
| X131472 |  |  |  |
| 0 | 469783 (97.6%) | 1240 (97.2%) | 471023 (97.6%) |
| 1 | 11649 (2.4%) | 36 (2.8%) | 11685 (2.4%) |
| X131494 |  |  |  |
| 0 | 424820 (88.2%) | 1065 (83.5%) | 425885 (88.2%) |
| 1 | 56612 (11.8%) | 211 (16.5%) | 56823 (11.8%) |
| X131584 |  |  |  |
| 0 | 446187 (92.7%) | 1153 (90.4%) | 447340 (92.7%) |
| 1 | 35245 (7.3%) | 123 (9.6%) | 35368 (7.3%) |
| X131598 |  |  |  |
| 0 | 459312 (95.4%) | 1173 (91.9%) | 460485 (95.4%) |
| 1 | 22120 (4.6%) | 103 (8.1%) | 22223 (4.6%) |
| X131600 |  |  |  |
| 0 | 471599 (98.0%) | 1234 (96.7%) | 472833 (98.0%) |
| 1 | 9833 (2.0%) | 42 (3.3%) | 9875 (2.0%) |
| X131612 |  |  |  |
| 0 | 466082 (96.8%) | 1239 (97.1%) | 467321 (96.8%) |
| 1 | 15350 (3.2%) | 37 (2.9%) | 15387 (3.2%) |
| X131620 |  |  |  |
| 0 | 459447 (95.4%) | 1184 (92.8%) | 460631 (95.4%) |
| 1 | 21985 (4.6%) | 92 (7.2%) | 22077 (4.6%) |
| X131630 |  |  |  |
| 0 | 467803 (97.2%) | 1197 (93.8%) | 469000 (97.2%) |
| 1 | 13629 (2.8%) | 79 (6.2%) | 13708 (2.8%) |
| X131636 |  |  |  |
| 0 | 465718 (96.7%) | 1219 (95.5%) | 466937 (96.7%) |
| 1 | 15714 (3.3%) | 57 (4.5%) | 15771 (3.3%) |
| X131638 |  |  |  |
| 0 | 456584 (94.8%) | 1187 (93.0%) | 457771 (94.8%) |
| 1 | 24848 (5.2%) | 89 (7.0%) | 24937 (5.2%) |
| X131640 |  |  |  |
| 0 | 473505 (98.4%) | 1227 (96.2%) | 474732 (98.3%) |
| 1 | 7927 (1.6%) | 49 (3.8%) | 7976 (1.7%) |
| X131646 |  |  |  |
| 0 | 460924 (95.7%) | 1200 (94.0%) | 462124 (95.7%) |
| 1 | 20508 (4.3%) | 76 (6.0%) | 20584 (4.3%) |
| X131650 |  |  |  |
| 0 | 460891 (95.7%) | 1232 (96.6%) | 462123 (95.7%) |
| 1 | 20541 (4.3%) | 44 (3.4%) | 20585 (4.3%) |
| X131674 |  |  |  |
| 0 | 465674 (96.7%) | 1237 (96.9%) | 466911 (96.7%) |
| 1 | 15758 (3.3%) | 39 (3.1%) | 15797 (3.3%) |
| X131702 |  |  |  |
| 0 | 468037 (97.2%) | 1224 (95.9%) | 469261 (97.2%) |
| 1 | 13395 (2.8%) | 52 (4.1%) | 13447 (2.8%) |
| X131708 |  |  |  |
| 0 | 474064 (98.5%) | 1252 (98.1%) | 475316 (98.5%) |
| 1 | 7368 (1.5%) | 24 (1.9%) | 7392 (1.5%) |
| X131720 |  |  |  |
| 0 | 471641 (98.0%) | 1256 (98.4%) | 472897 (98.0%) |
| 1 | 9791 (2.0%) | 20 (1.6%) | 9811 (2.0%) |
| X131740 |  |  |  |
| 0 | 448205 (93.1%) | 1198 (93.9%) | 449403 (93.1%) |
| 1 | 33227 (6.9%) | 78 (6.1%) | 33305 (6.9%) |
| X131742 |  |  |  |
| 0 | 470726 (97.8%) | 1251 (98.0%) | 471977 (97.8%) |
| 1 | 10706 (2.2%) | 25 (2.0%) | 10731 (2.2%) |
| X131768 |  |  |  |
| 0 | 474377 (98.5%) | 1261 (98.8%) | 475638 (98.5%) |
| 1 | 7055 (1.5%) | 15 (1.2%) | 7070 (1.5%) |
| X131794 |  |  |  |
| 0 | 472376 (98.1%) | 1253 (98.2%) | 473629 (98.1%) |
| 1 | 9056 (1.9%) | 23 (1.8%) | 9079 (1.9%) |
| X131806 |  |  |  |
| 0 | 467975 (97.2%) | 1250 (98.0%) | 469225 (97.2%) |
| 1 | 13457 (2.8%) | 26 (2.0%) | 13483 (2.8%) |
| X131836 |  |  |  |
| 0 | 459083 (95.4%) | 1229 (96.3%) | 460312 (95.4%) |
| 1 | 22349 (4.6%) | 47 (3.7%) | 22396 (4.6%) |
| X131858 |  |  |  |
| 0 | 471131 (97.9%) | 1250 (98.0%) | 472381 (97.9%) |
| 1 | 10301 (2.1%) | 26 (2.0%) | 10327 (2.1%) |
| X131864 |  |  |  |
| 0 | 468729 (97.4%) | 1236 (96.9%) | 469965 (97.4%) |
| 1 | 12703 (2.6%) | 40 (3.1%) | 12743 (2.6%) |
| X131872 |  |  |  |
| 0 | 467760 (97.2%) | 1221 (95.7%) | 468981 (97.2%) |
| 1 | 13672 (2.8%) | 55 (4.3%) | 13727 (2.8%) |
| X131876 |  |  |  |
| 0 | 430914 (89.5%) | 1116 (87.5%) | 432030 (89.5%) |
| 1 | 50518 (10.5%) | 160 (12.5%) | 50678 (10.5%) |
| X131878 |  |  |  |
| 0 | 470797 (97.8%) | 1242 (97.3%) | 472039 (97.8%) |
| 1 | 10635 (2.2%) | 34 (2.7%) | 10669 (2.2%) |
| X131884 |  |  |  |
| 0 | 470964 (97.8%) | 1252 (98.1%) | 472216 (97.8%) |
| 1 | 10468 (2.2%) | 24 (1.9%) | 10492 (2.2%) |
| X131888 |  |  |  |
| 0 | 428908 (89.1%) | 1140 (89.3%) | 430048 (89.1%) |
| 1 | 52524 (10.9%) | 136 (10.7%) | 52660 (10.9%) |
| X131916 |  |  |  |
| 0 | 465059 (96.6%) | 1213 (95.1%) | 466272 (96.6%) |
| 1 | 16373 (3.4%) | 63 (4.9%) | 16436 (3.4%) |
| X131924 |  |  |  |
| 0 | 465817 (96.8%) | 1241 (97.3%) | 467058 (96.8%) |
| 1 | 15615 (3.2%) | 35 (2.7%) | 15650 (3.2%) |
| X131928 |  |  |  |
| 0 | 435459 (90.5%) | 1134 (88.9%) | 436593 (90.4%) |
| 1 | 45973 (9.5%) | 142 (11.1%) | 46115 (9.6%) |
| X131938 |  |  |  |
| 0 | 471972 (98.0%) | 1253 (98.2%) | 473225 (98.0%) |
| 1 | 9460 (2.0%) | 23 (1.8%) | 9483 (2.0%) |
| X131946 |  |  |  |
| 0 | 473338 (98.3%) | 1260 (98.7%) | 474598 (98.3%) |
| 1 | 8094 (1.7%) | 16 (1.3%) | 8110 (1.7%) |
| X131950 |  |  |  |
| 0 | 470458 (97.7%) | 1253 (98.2%) | 471711 (97.7%) |
| 1 | 10974 (2.3%) | 23 (1.8%) | 10997 (2.3%) |
| X131954 |  |  |  |
| 0 | 465470 (96.7%) | 1241 (97.3%) | 466711 (96.7%) |
| 1 | 15962 (3.3%) | 35 (2.7%) | 15997 (3.3%) |
| X131958 |  |  |  |
| 0 | 460117 (95.6%) | 1232 (96.6%) | 461349 (95.6%) |
| 1 | 21315 (4.4%) | 44 (3.4%) | 21359 (4.4%) |
| X131960 |  |  |  |
| 0 | 437061 (90.8%) | 1144 (89.7%) | 438205 (90.8%) |
| 1 | 44371 (9.2%) | 132 (10.3%) | 44503 (9.2%) |
| X131964 |  |  |  |
| 0 | 471998 (98.0%) | 1233 (96.6%) | 473231 (98.0%) |
| 1 | 9434 (2.0%) | 43 (3.4%) | 9477 (2.0%) |
| X132054 |  |  |  |
| 0 | 470398 (97.7%) | 1239 (97.1%) | 471637 (97.7%) |
| 1 | 11034 (2.3%) | 37 (2.9%) | 11071 (2.3%) |
| X132070 |  |  |  |
| 0 | 454299 (94.4%) | 1180 (92.5%) | 455479 (94.4%) |
| 1 | 27133 (5.6%) | 96 (7.5%) | 27229 (5.6%) |
| X132072 |  |  |  |
| 0 | 468082 (97.2%) | 1238 (97.0%) | 469320 (97.2%) |
| 1 | 13350 (2.8%) | 38 (3.0%) | 13388 (2.8%) |
| X132096 |  |  |  |
| 0 | 470829 (97.8%) | 1256 (98.4%) | 472085 (97.8%) |
| 1 | 10603 (2.2%) | 20 (1.6%) | 10623 (2.2%) |
| X132102 |  |  |  |
| 0 | 468828 (97.4%) | 1246 (97.6%) | 470074 (97.4%) |
| 1 | 12604 (2.6%) | 30 (2.4%) | 12634 (2.6%) |
| X132104 |  |  |  |
| 0 | 472428 (98.1%) | 1261 (98.8%) | 473689 (98.1%) |
| 1 | 9004 (1.9%) | 15 (1.2%) | 9019 (1.9%) |
| X132124 |  |  |  |
| 0 | 470769 (97.8%) | 1240 (97.2%) | 472009 (97.8%) |
| 1 | 10663 (2.2%) | 36 (2.8%) | 10699 (2.2%) |
| X132128 |  |  |  |
| 0 | 472078 (98.1%) | 1243 (97.4%) | 473321 (98.1%) |
| 1 | 9354 (1.9%) | 33 (2.6%) | 9387 (1.9%) |
| X132130 |  |  |  |
| 0 | 469894 (97.6%) | 1251 (98.0%) | 471145 (97.6%) |
| 1 | 11538 (2.4%) | 25 (2.0%) | 11563 (2.4%) |
| X132140 |  |  |  |
| 0 | 473079 (98.3%) | 1255 (98.4%) | 474334 (98.3%) |
| 1 | 8353 (1.7%) | 21 (1.6%) | 8374 (1.7%) |
| X132146 |  |  |  |
| 0 | 457673 (95.1%) | 1226 (96.1%) | 458899 (95.1%) |
| 1 | 23759 (4.9%) | 50 (3.9%) | 23809 (4.9%) |
| X132148 |  |  |  |
| 0 | 473260 (98.3%) | 1260 (98.7%) | 474520 (98.3%) |
| 1 | 8172 (1.7%) | 16 (1.3%) | 8188 (1.7%) |
| X132150 |  |  |  |
| 0 | 471324 (97.9%) | 1246 (97.6%) | 472570 (97.9%) |
| 1 | 10108 (2.1%) | 30 (2.4%) | 10138 (2.1%) |
| X132276 |  |  |  |
| 0 | 466782 (97.0%) | 1244 (97.5%) | 468026 (97.0%) |
| 1 | 14650 (3.0%) | 32 (2.5%) | 14682 (3.0%) |
| Hypertension |  |  |  |
| 0 | 65764 (13.7%) | 280 (21.9%) | 66044 (13.7%) |
| 1 | 129000 (26.8%) | 447 (35.0%) | 129447 (26.8%) |
| Missing | 286668 (59.5%) | 549 (43.0%) | 287217 (59.5%) |
| Hearing loss |  |  |  |
| 0 | 18378 (3.8%) | 110 (8.6%) | 18488 (3.8%) |
| 1 | 8535 (1.8%) | 30 (2.4%) | 8565 (1.8%) |
| Missing | 454519 (94.4%) | 1136 (89.0%) | 455655 (94.4%) |
| Vision loss |  |  |  |
| 0 | 3389 (0.7%) | 34 (2.7%) | 3423 (0.7%) |
| 1 | 861 (0.2%) | 5 (0.4%) | 866 (0.2%) |
| Missing | 477182 (99.1%) | 1237 (96.9%) | 478419 (99.1%) |
| TBI |  |  |  |
| 0 | 15424 (3.2%) | 216 (16.9%) | 15640 (3.2%) |
| 1 | 5531 (1.1%) | 44 (3.4%) | 5575 (1.2%) |
| Missing | 460477 (95.6%) | 1016 (79.6%) | 461493 (95.6%) |
| p132152 |  |  |  |
| 0 | 14450 (3.0%) | 50 (3.9%) | 14500 (3.0%) |
| 1 | 23684 (4.9%) | 68 (5.3%) | 23752 (4.9%) |
| Missing | 443298 (92.1%) | 1158 (90.8%) | 444456 (92.1%) |
| X1050 |  |  |  |
| Low | 160209 (33.3%) | 402 (31.5%) | 160611 (33.3%) |
| Medium | 149837 (31.1%) | 351 (27.5%) | 150188 (31.1%) |
| High | 143339 (29.8%) | 398 (31.2%) | 143737 (29.8%) |
| Missing | 28047 (5.8%) | 125 (9.8%) | 28172 (5.8%) |
| X1060 |  |  |  |
| Low | 239704 (49.8%) | 532 (41.7%) | 240236 (49.8%) |
| Medium | 108291 (22.5%) | 278 (21.8%) | 108569 (22.5%) |
| High | 105161 (21.8%) | 356 (27.9%) | 105517 (21.9%) |
| Missing | 28276 (5.9%) | 110 (8.6%) | 28386 (5.9%) |
| X1070 |  |  |  |
| Low | 225487 (46.8%) | 506 (39.7%) | 225993 (46.8%) |
| Medium | 112586 (23.4%) | 247 (19.4%) | 112833 (23.4%) |
| High | 139257 (28.9%) | 496 (38.9%) | 139753 (29.0%) |
| Missing | 4102 (0.9%) | 27 (2.1%) | 4129 (0.9%) |
| X1080 |  |  |  |
| Low | 232311 (48.3%) | 730 (57.2%) | 233041 (48.3%) |
| Medium | 136553 (28.4%) | 262 (20.5%) | 136815 (28.3%) |
| High | 107860 (22.4%) | 238 (18.7%) | 108098 (22.4%) |
| Missing | 4708 (1.0%) | 46 (3.6%) | 4754 (1.0%) |
| X1488 |  |  |  |
| Low | 194948 (40.5%) | 511 (40.0%) | 195459 (40.5%) |
| Medium | 140890 (29.3%) | 342 (26.8%) | 141232 (29.3%) |
| High | 144517 (30.0%) | 413 (32.4%) | 144930 (30.0%) |
| Missing | 1077 (0.2%) | 10 (0.8%) | 1087 (0.2%) |
| X1528 |  |  |  |
| Low | 266568 (55.4%) | 652 (51.1%) | 267220 (55.4%) |
| Medium | 72343 (15.0%) | 188 (14.7%) | 72531 (15.0%) |
| High | 138972 (28.9%) | 411 (32.2%) | 139383 (28.9%) |
| Missing | 3549 (0.7%) | 25 (2.0%) | 3574 (0.7%) |
| X2139 |  |  |  |
| Low | 153785 (31.9%) | 374 (29.3%) | 154159 (31.9%) |
| Medium | 147812 (30.7%) | 301 (23.6%) | 148113 (30.7%) |
| High | 117359 (24.4%) | 280 (21.9%) | 117639 (24.4%) |
| Missing | 62476 (13.0%) | 321 (25.2%) | 62797 (13.0%) |
| X2149 |  |  |  |
| Low | 163148 (33.9%) | 318 (24.9%) | 163466 (33.9%) |
| Medium | 106189 (22.1%) | 250 (19.6%) | 106439 (22.1%) |
| High | 120622 (25.1%) | 293 (23.0%) | 120915 (25.0%) |
| Missing | 91473 (19.0%) | 415 (32.5%) | 91888 (19.0%) |
| X22037 |  |  |  |
| Low | 129625 (26.9%) | 324 (25.4%) | 129949 (26.9%) |
| Medium | 124816 (25.9%) | 287 (22.5%) | 125103 (25.9%) |
| High | 118375 (24.6%) | 283 (22.2%) | 118658 (24.6%) |
| Missing | 108616 (22.6%) | 382 (29.9%) | 108998 (22.6%) |
| X22038 |  |  |  |
| Low | 143579 (29.8%) | 343 (26.9%) | 143922 (29.8%) |
| Medium | 109094 (22.7%) | 254 (19.9%) | 109348 (22.7%) |
| High | 120143 (25.0%) | 297 (23.3%) | 120440 (25.0%) |
| Missing | 108616 (22.6%) | 382 (29.9%) | 108998 (22.6%) |
| X22039 |  |  |  |
| Low | 146260 (30.4%) | 439 (34.4%) | 146699 (30.4%) |
| Medium | 102705 (21.3%) | 185 (14.5%) | 102890 (21.3%) |
| High | 123851 (25.7%) | 270 (21.2%) | 124121 (25.7%) |
| Missing | 108616 (22.6%) | 382 (29.9%) | 108998 (22.6%) |
| X23099 |  |  |  |
| Low | 158498 (32.9%) | 371 (29.1%) | 158869 (32.9%) |
| Medium | 158424 (32.9%) | 404 (31.7%) | 158828 (32.9%) |
| High | 155734 (32.3%) | 451 (35.3%) | 156185 (32.4%) |
| Missing | 8776 (1.8%) | 50 (3.9%) | 8826 (1.8%) |
| X23127 |  |  |  |
| Low | 158503 (32.9%) | 361 (28.3%) | 158864 (32.9%) |
| Medium | 159282 (33.1%) | 397 (31.1%) | 159679 (33.1%) |
| High | 154843 (32.2%) | 468 (36.7%) | 155311 (32.2%) |
| Missing | 8804 (1.8%) | 50 (3.9%) | 8854 (1.8%) |
| X24006 |  |  |  |
| Low | 149222 (31.0%) | 297 (23.3%) | 149519 (31.0%) |
| Medium | 147054 (30.5%) | 348 (27.3%) | 147402 (30.5%) |
| High | 145897 (30.3%) | 522 (40.9%) | 146419 (30.3%) |
| Missing | 39259 (8.2%) | 109 (8.5%) | 39368 (8.2%) |
| X24008 |  |  |  |
| Low | 149538 (31.1%) | 342 (26.8%) | 149880 (31.0%) |
| Medium | 147405 (30.6%) | 425 (33.3%) | 147830 (30.6%) |
| High | 145230 (30.2%) | 400 (31.3%) | 145630 (30.2%) |
| Missing | 39259 (8.2%) | 109 (8.5%) | 39368 (8.2%) |
| X24020 |  |  |  |
| Low | 158998 (33.0%) | 393 (30.8%) | 159391 (33.0%) |
| Medium | 158254 (32.9%) | 417 (32.7%) | 158671 (32.9%) |
| High | 157676 (32.8%) | 434 (34.0%) | 158110 (32.8%) |
| Missing | 6504 (1.4%) | 32 (2.5%) | 6536 (1.4%) |
| X24021 |  |  |  |
| Low | 159312 (33.1%) | 394 (30.9%) | 159706 (33.1%) |
| Medium | 157634 (32.7%) | 416 (32.6%) | 158050 (32.7%) |
| High | 157982 (32.8%) | 434 (34.0%) | 158416 (32.8%) |
| Missing | 6504 (1.4%) | 32 (2.5%) | 6536 (1.4%) |
| X24022 |  |  |  |
| Low | 158989 (33.0%) | 393 (30.8%) | 159382 (33.0%) |
| Medium | 158258 (32.9%) | 417 (32.7%) | 158675 (32.9%) |
| High | 157681 (32.8%) | 434 (34.0%) | 158115 (32.8%) |
| Missing | 6504 (1.4%) | 32 (2.5%) | 6536 (1.4%) |
| X24500 |  |  |  |
| Low | 139187 (28.9%) | 474 (37.1%) | 139661 (28.9%) |
| Medium | 140068 (29.1%) | 390 (30.6%) | 140458 (29.1%) |
| High | 142851 (29.7%) | 262 (20.5%) | 143113 (29.6%) |
| Missing | 59326 (12.3%) | 150 (11.8%) | 59476 (12.3%) |
| X24501 |  |  |  |
| Low | 142026 (29.5%) | 388 (30.4%) | 142414 (29.5%) |
| Medium | 140876 (29.3%) | 377 (29.5%) | 141253 (29.3%) |
| High | 139204 (28.9%) | 361 (28.3%) | 139565 (28.9%) |
| Missing | 59326 (12.3%) | 150 (11.8%) | 59476 (12.3%) |
| X24502 |  |  |  |
| Low | 140409 (29.2%) | 385 (30.2%) | 140794 (29.2%) |
| Medium | 140636 (29.2%) | 336 (26.3%) | 140972 (29.2%) |
| High | 141061 (29.3%) | 405 (31.7%) | 141466 (29.3%) |
| Missing | 59326 (12.3%) | 150 (11.8%) | 59476 (12.3%) |
| X24503 |  |  |  |
| Low | 139546 (29.0%) | 416 (32.6%) | 139962 (29.0%) |
| Medium | 140287 (29.1%) | 422 (33.1%) | 140709 (29.2%) |
| High | 142273 (29.6%) | 288 (22.6%) | 142561 (29.5%) |
| Missing | 59326 (12.3%) | 150 (11.8%) | 59476 (12.3%) |
| X24504 |  |  |  |
| Low | 141393 (29.4%) | 419 (32.8%) | 141812 (29.4%) |
| Medium | 140532 (29.2%) | 374 (29.3%) | 140906 (29.2%) |
| High | 140181 (29.1%) | 333 (26.1%) | 140514 (29.1%) |
| Missing | 59326 (12.3%) | 150 (11.8%) | 59476 (12.3%) |
| X24505 |  |  |  |
| Low | 141616 (29.4%) | 413 (32.4%) | 142029 (29.4%) |
| Medium | 139542 (29.0%) | 326 (25.5%) | 139868 (29.0%) |
| High | 140948 (29.3%) | 387 (30.3%) | 141335 (29.3%) |
| Missing | 59326 (12.3%) | 150 (11.8%) | 59476 (12.3%) |
| X24506 |  |  |  |
| Low | 156954 (32.6%) | 538 (42.2%) | 157492 (32.6%) |
| Medium | 158696 (33.0%) | 432 (33.9%) | 159128 (33.0%) |
| High | 161152 (33.5%) | 290 (22.7%) | 161442 (33.4%) |
| Missing | 4630 (1.0%) | 16 (1.3%) | 4646 (1.0%) |
| X24507 |  |  |  |
| Low | 159300 (33.1%) | 483 (37.9%) | 159783 (33.1%) |
| Medium | 157532 (32.7%) | 445 (34.9%) | 157977 (32.7%) |
| High | 159970 (33.2%) | 332 (26.0%) | 160302 (33.2%) |
| Missing | 4630 (1.0%) | 16 (1.3%) | 4646 (1.0%) |
| X22189 |  |  |  |
| Low | 162836 (33.8%) | 264 (20.7%) | 163100 (33.8%) |
| Medium | 159773 (33.2%) | 331 (25.9%) | 160104 (33.2%) |
| High | 158235 (32.9%) | 677 (53.1%) | 158912 (32.9%) |
| Missing | 588 (0.1%) | 4 (0.3%) | 592 (0.1%) |
| X23105 |  |  |  |
| Low | 157916 (32.8%) | 386 (30.3%) | 158302 (32.8%) |
| Medium | 157289 (32.7%) | 431 (33.8%) | 157720 (32.7%) |
| High | 157697 (32.8%) | 412 (32.3%) | 158109 (32.8%) |
| Missing | 8530 (1.8%) | 47 (3.7%) | 8577 (1.8%) |
| X22040 |  |  |  |
| Low | 123985 (25.8%) | 328 (25.7%) | 124313 (25.8%) |
| Medium | 124248 (25.8%) | 271 (21.2%) | 124519 (25.8%) |
| High | 124583 (25.9%) | 295 (23.1%) | 124878 (25.9%) |
| Missing | 108616 (22.6%) | 382 (29.9%) | 108998 (22.6%) |
| X1498 |  |  |  |
| Low | 237613 (49.4%) | 647 (50.7%) | 238260 (49.4%) |
| Medium | 90216 (18.7%) | 236 (18.5%) | 90452 (18.7%) |
| High | 152463 (31.7%) | 379 (29.7%) | 152842 (31.7%) |
| Missing | 1140 (0.2%) | 14 (1.1%) | 1154 (0.2%) |
| X24023 |  |  |  |
| Low | 158667 (33.0%) | 393 (30.8%) | 159060 (33.0%) |
| Medium | 158385 (32.9%) | 417 (32.7%) | 158802 (32.9%) |
| High | 157876 (32.8%) | 434 (34.0%) | 158310 (32.8%) |
| Missing | 6504 (1.4%) | 32 (2.5%) | 6536 (1.4%) |
| Leg fat |  |  |  |
| Low | 158530 (32.9%) | 398 (31.2%) | 158928 (32.9%) |
| Medium | 158213 (32.9%) | 382 (29.9%) | 158595 (32.9%) |
| High | 156140 (32.4%) | 449 (35.2%) | 156589 (32.4%) |
| Missing | 8549 (1.8%) | 47 (3.7%) | 8596 (1.8%) |
| Arm fat |  |  |  |
| Low | 158797 (33.0%) | 367 (28.8%) | 159164 (33.0%) |
| Medium | 157901 (32.8%) | 398 (31.2%) | 158299 (32.8%) |
| High | 156112 (32.4%) | 464 (36.4%) | 156576 (32.4%) |
| Missing | 8622 (1.8%) | 47 (3.7%) | 8669 (1.8%) |
| Lung function |  |  |  |
| Low | 145228 (30.2%) | 500 (39.2%) | 145728 (30.2%) |
| Medium | 146419 (30.4%) | 319 (25.0%) | 146738 (30.4%) |
| High | 147289 (30.6%) | 261 (20.5%) | 147550 (30.6%) |
| Missing | 42496 (8.8%) | 196 (15.4%) | 42692 (8.8%) |
| DBP |  |  |  |
| Low | 154696 (32.1%) | 433 (33.9%) | 155129 (32.1%) |
| Medium | 148517 (30.8%) | 349 (27.4%) | 148866 (30.8%) |
| High | 151368 (31.4%) | 424 (33.2%) | 151792 (31.4%) |
| Missing | 26851 (5.6%) | 70 (5.5%) | 26921 (5.6%) |
| SBP |  |  |  |
| Low | 152599 (31.7%) | 430 (33.7%) | 153029 (31.7%) |
| Medium | 152720 (31.7%) | 374 (29.3%) | 153094 (31.7%) |
| High | 149257 (31.0%) | 402 (31.5%) | 149659 (31.0%) |
| Missing | 26856 (5.6%) | 70 (5.5%) | 26926 (5.6%) |
| Hand grip |  |  |  |
| Low | 161471 (33.5%) | 528 (41.4%) | 161999 (33.6%) |
| Medium | 162517 (33.8%) | 413 (32.4%) | 162930 (33.8%) |
| High | 156222 (32.4%) | 327 (25.6%) | 156549 (32.4%) |
| Missing | 1222 (0.3%) | 8 (0.6%) | 1230 (0.3%) |
| Nitrogen dioxide |  |  |  |
| Low | 160588 (33.4%) | 289 (22.6%) | 160877 (33.3%) |
| Medium | 158999 (33.0%) | 406 (31.8%) | 159405 (33.0%) |
| High | 155341 (32.3%) | 549 (43.0%) | 155890 (32.3%) |
| Missing | 6504 (1.4%) | 32 (2.5%) | 6536 (1.4%) |
| PM10 |  |  |  |
| Low | 160060 (33.2%) | 306 (24.0%) | 160366 (33.2%) |
| Medium | 158629 (32.9%) | 410 (32.1%) | 159039 (32.9%) |
| High | 156228 (32.5%) | 528 (41.4%) | 156756 (32.5%) |
| Missing | 6515 (1.4%) | 32 (2.5%) | 6547 (1.4%) |
| Vegetable intake |  |  |  |
| Low | 166100 (34.5%) | 499 (39.1%) | 166599 (34.5%) |
| Medium | 164996 (34.3%) | 378 (29.6%) | 165374 (34.3%) |
| High | 150336 (31.2%) | 399 (31.3%) | 150735 (31.2%) |
| Diet score |  |  |  |
| Low | 286612 (59.5%) | 824 (64.6%) | 287436 (59.5%) |
| Medium | 135808 (28.2%) | 319 (25.0%) | 136127 (28.2%) |
| High | 59012 (12.3%) | 133 (10.4%) | 59145 (12.3%) |
| X24024 |  |  |  |
| Low | 158624 (32.9%) | 393 (30.8%) | 159017 (32.9%) |
| Medium | 158404 (32.9%) | 417 (32.7%) | 158821 (32.9%) |
| High | 157900 (32.8%) | 434 (34.0%) | 158334 (32.8%) |
| Missing | 6504 (1.4%) | 32 (2.5%) | 6536 (1.4%) |
| Chronic heart disease |  |  |  |
| 0 | 455422 (94.6%) | 1170 (91.7%) | 456592 (94.6%) |
| 1 | 26010 (5.4%) | 106 (8.3%) | 26116 (5.4%) |
| Stroke |  |  |  |
| 0 | 473674 (98.4%) | 1230 (96.4%) | 474904 (98.4%) |
| 1 | 7758 (1.6%) | 46 (3.6%) | 7804 (1.6%) |
| Diabetes |  |  |  |
| 0 | 456581 (94.8%) | 1159 (90.8%) | 457740 (94.8%) |
| 1 | 24851 (5.2%) | 117 (9.2%) | 24968 (5.2%) |
| COPD |  |  |  |
| 0 | 472078 (98.1%) | 1223 (95.8%) | 473301 (98.1%) |
| 1 | 9354 (1.9%) | 53 (4.2%) | 9407 (1.9%) |
| Arthritis |  |  |  |
| 0 | 419107 (87.1%) | 1068 (83.7%) | 420175 (87.0%) |
| 1 | 62325 (12.9%) | 208 (16.3%) | 62533 (13.0%) |
| Dementia |  |  |  |
| 0 | 481250 (100.0%) | 1273 (99.8%) | 482523 (100.0%) |
| 1 | 182 (0.0%) | 3 (0.2%) | 185 (0.0%) |
| Other organic disease |  |  |  |
| 0 | 480752 (99.9%) | 1238 (97.0%) | 481990 (99.9%) |
| 1 | 680 (0.1%) | 38 (3.0%) | 718 (0.1%) |
| X130854 |  |  |  |
| 0 | 477557 (99.2%) | 1222 (95.8%) | 478779 (99.2%) |
| 1 | 3875 (0.8%) | 54 (4.2%) | 3929 (0.8%) |
| X130856 |  |  |  |
| 0 | 481298 (100.0%) | 1271 (99.6%) | 482569 (100.0%) |
| 1 | 134 (0.0%) | 5 (0.4%) | 139 (0.0%) |
| X130858 |  |  |  |
| 0 | 481348 (100.0%) | 1272 (99.7%) | 482620 (100.0%) |
| 1 | 84 (0.0%) | 4 (0.3%) | 88 (0.0%) |
| X130860 |  |  |  |
| 0 | 481260 (100.0%) | 1275 (99.9%) | 482535 (100.0%) |
| 1 | 172 (0.0%) | 1 (0.1%) | 173 (0.0%) |
| Other mental disorders |  |  |  |
| 0 | 481367 (100.0%) | 1275 (99.9%) | 482642 (100.0%) |
| 1 | 65 (0.0%) | 1 (0.1%) | 66 (0.0%) |
| X130872 |  |  |  |
| 0 | 481260 (100.0%) | 1269 (99.5%) | 482529 (100.0%) |
| 1 | 172 (0.0%) | 7 (0.5%) | 179 (0.0%) |
| X130890 |  |  |  |
| 0 | 481235 (100.0%) | 1256 (98.4%) | 482491 (100.0%) |
| 1 | 197 (0.0%) | 20 (1.6%) | 217 (0.0%) |
| X130892 |  |  |  |
| 0 | 480151 (99.7%) | 1163 (91.1%) | 481314 (99.7%) |
| 1 | 1281 (0.3%) | 113 (8.9%) | 1394 (0.3%) |
| X130894 |  |  |  |
| 0 | 442428 (91.9%) | 893 (70.0%) | 443321 (91.8%) |
| 1 | 39004 (8.1%) | 383 (30.0%) | 39387 (8.2%) |
| X130896 |  |  |  |
| 0 | 478979 (99.5%) | 1220 (95.6%) | 480199 (99.5%) |
| 1 | 2453 (0.5%) | 56 (4.4%) | 2509 (0.5%) |
| X130898 |  |  |  |
| 0 | 480802 (99.9%) | 1267 (99.3%) | 482069 (99.9%) |
| 1 | 630 (0.1%) | 9 (0.7%) | 639 (0.1%) |
| Other mood disorders |  |  |  |
| 0 | 480806 (99.9%) | 1268 (99.4%) | 482074 (99.9%) |
| 1 | 626 (0.1%) | 8 (0.6%) | 634 (0.1%) |
| X130904 |  |  |  |
| 0 | 479937 (99.7%) | 1267 (99.3%) | 481204 (99.7%) |
| 1 | 1495 (0.3%) | 9 (0.7%) | 1504 (0.3%) |
| X130908 |  |  |  |
| 0 | 480870 (99.9%) | 1259 (98.7%) | 482129 (99.9%) |
| 1 | 562 (0.1%) | 17 (1.3%) | 579 (0.1%) |
| Other behavior disorders |  |  |  |
| 0 | 481414 (100.0%) | 1275 (99.9%) | 482689 (100.0%) |
| 1 | 18 (0.0%) | 1 (0.1%) | 19 (0.0%) |
| X130932 |  |  |  |
| 0 | 481104 (99.9%) | 1259 (98.7%) | 482363 (99.9%) |
| 1 | 328 (0.1%) | 17 (1.3%) | 345 (0.1%) |
| X130912 |  |  |  |
| 0 | 481286 (100.0%) | 1269 (99.5%) | 482555 (100.0%) |
| 1 | 146 (0.0%) | 7 (0.5%) | 153 (0.0%) |
| X130914 |  |  |  |
| 0 | 479618 (99.6%) | 1264 (99.1%) | 480882 (99.6%) |
| 1 | 1814 (0.4%) | 12 (0.9%) | 1826 (0.4%) |
| X130916 |  |  |  |
| 0 | 480568 (99.8%) | 1241 (97.3%) | 481809 (99.8%) |
| 1 | 864 (0.2%) | 35 (2.7%) | 899 (0.2%) |
| X130918 |  |  |  |
| 0 | 480639 (99.8%) | 1266 (99.2%) | 481905 (99.8%) |
| 1 | 793 (0.2%) | 10 (0.8%) | 803 (0.2%) |
| X130920 |  |  |  |
| 0 | 481075 (99.9%) | 1274 (99.8%) | 482349 (99.9%) |
| 1 | 357 (0.1%) | 2 (0.2%) | 359 (0.1%) |
| X130922 |  |  |  |
| 0 | 480097 (99.7%) | 1269 (99.5%) | 481366 (99.7%) |
| 1 | 1335 (0.3%) | 7 (0.5%) | 1342 (0.3%) |
| X130924 |  |  |  |
| 0 | 480153 (99.7%) | 1262 (98.9%) | 481415 (99.7%) |
| 1 | 1279 (0.3%) | 14 (1.1%) | 1293 (0.3%) |
| Other adult disorders |  |  |  |
| 0 | 481225 (100.0%) | 1274 (99.8%) | 482499 (100.0%) |
| 1 | 207 (0.0%) | 2 (0.2%) | 209 (0.0%) |
| X130944 |  |  |  |
| 0 | 481080 (99.9%) | 1275 (99.9%) | 482355 (99.9%) |
| 1 | 352 (0.1%) | 1 (0.1%) | 353 (0.1%) |
| Mental retardation |  |  |  |
| 0 | 481396 (100.0%) | 1273 (99.8%) | 482669 (100.0%) |
| 1 | 36 (0.0%) | 3 (0.2%) | 39 (0.0%) |
| Psychological development |  |  |  |
| 0 | 481208 (100.0%) | 1263 (99.0%) | 482471 (100.0%) |
| 1 | 224 (0.0%) | 13 (1.0%) | 237 (0.0%) |
| Emotional disorders |  |  |  |
| 0 | 481174 (99.9%) | 1276 (100%) | 482450 (99.9%) |
| 1 | 258 (0.1%) | 0 (0%) | 258 (0.1%) |
| X130990 |  |  |  |
| 0 | 481298 (100.0%) | 1263 (99.0%) | 482561 (100.0%) |
| 1 | 134 (0.0%) | 13 (1.0%) | 147 (0.0%) |
| Depressed mood |  |  |  |
| 0 | 351690 (73.1%) | 669 (52.4%) | 352359 (73.0%) |
| 1 | 107762 (22.4%) | 519 (40.7%) | 108281 (22.4%) |
| Missing | 21980 (4.6%) | 88 (6.9%) | 22068 (4.6%) |
| Friend visit |  |  |  |
| 0 | 8153 (1.7%) | 91 (7.1%) | 8244 (1.7%) |
| 1 | 469908 (97.6%) | 1155 (90.5%) | 471063 (97.6%) |
| Missing | 3371 (0.7%) | 30 (2.4%) | 3401 (0.7%) |
| Tenseness restlessness |  |  |  |
| 0 | 339045 (70.4%) | 664 (52.0%) | 339709 (70.4%) |
| 1 | 122349 (25.4%) | 532 (41.7%) | 122881 (25.5%) |
| Missing | 20038 (4.2%) | 80 (6.3%) | 20118 (4.2%) |
| Tiredness lethargy |  |  |  |
| 0 | 217973 (45.3%) | 416 (32.6%) | 218389 (45.2%) |
| 1 | 248050 (51.5%) | 797 (62.5%) | 248847 (51.6%) |
| Missing | 15409 (3.2%) | 63 (4.9%) | 15472 (3.2%) |
| Unenthusiasm disinterest |  |  |  |
| 0 | 365098 (75.8%) | 716 (56.1%) | 365814 (75.8%) |
| 1 | 98583 (20.5%) | 476 (37.3%) | 99059 (20.5%) |
| Missing | 17751 (3.7%) | 84 (6.6%) | 17835 (3.7%) |
| Illness injury |  |  |  |
| 0 | 264077 (54.9%) | 564 (44.2%) | 264641 (54.8%) |
| 1 | 213762 (44.4%) | 685 (53.7%) | 214447 (44.4%) |
| Missing | 3593 (0.7%) | 27 (2.1%) | 3620 (0.8%) |
| risk_taking |  |  |  |
| 0 | 338561 (70.3%) | 827 (64.8%) | 339388 (70.3%) |
| 1 | 124433 (25.8%) | 365 (28.6%) | 124798 (25.9%) |
| Missing | 18438 (3.8%) | 84 (6.6%) | 18522 (3.8%) |
| Ever seen psychiatrist |  |  |  |
| 0 | 425428 (88.4%) | 660 (51.7%) | 426088 (88.3%) |
| 1 | 53590 (11.1%) | 585 (45.8%) | 54175 (11.2%) |
| Missing | 2414 (0.5%) | 31 (2.4%) | 2445 (0.5%) |
| Ever seen doctor |  |  |  |
| 0 | 316460 (65.7%) | 457 (35.8%) | 316917 (65.7%) |
| 1 | 161296 (33.5%) | 794 (62.2%) | 162090 (33.6%) |
| Missing | 3676 (0.8%) | 25 (2.0%) | 3701 (0.8%) |

| **Supplementary Table 6. The baseline characteristics of participants by late onset schizophrenia.** | | | |
| --- | --- | --- | --- |
| Variables | Late onset schizophrenia | | P |
|  | No (N=481432) | Yes (N=1276) |  |
| Sex |  |  | 0.262 |
| Female | 261283 (54.3%) | 672 (52.7%) |  |
| Male | 220149 (45.7%) | 604 (47.3%) |  |
| Age |  |  | 0.008 |
| Mean (SD) | 56.6 (8.1) | 57.2 (8.4) |  |
| Median (P25, P75) | 58.0 [50.0, 63.0] | 59.0 [50.0, 65.0] |  |
| Education |  |  | <0.001 |
| Lower | 249811 (52.5) | 779 (62.1) |  |
| Higher | 226067 (47.5) | 475 (37.9) |  |
| Household Income |  |  | <0.001 |
| Less than 18000 | 92865 (22.6) | 526 (52.8) |  |
| 18000-30999 | 104864 (25.5) | 227 (22.8) |  |
| 31000-51999 | 107649 (26.2) | 148 (14.9) |  |
| 52000-100000 | 83974 (20.4) | 75 (7.5) |  |
| Greater than 100000 | 22299 (5.4) | 20 (2.0) |  |
| PRS |  |  | <0.001 |
| Low | 160629 (33.4%) | 275 (21.6%) |  |
| Medium | 160554 (33.3%) | 347 (27.2%) |  |
| High | 160249 (33.3%) | 654 (51.3%) |  |
| SD, Standard Deviation; PRS, polygenic risk score | | | |

| **Supplementary Table 7. Associations between 232 risk factors and late onset schizophrenia in EWAS.** Blue cells indicate P.adjust<0.05; P.adjust, after Bonferroni correction. | | | | | | | | | |
| --- | --- | --- | --- | --- | --- | --- | --- | --- | --- |
| **FieldID** | **HR** | **CI5** | **CI95** | **P.adjust** | **case** | **Full sample** | **P.value.for.Schoenfeld.residuals** | **Exposures** | **Category** |
| Alcohol_level | 1.53 | 1.36 | 1.72 | 1.49×10^-10^ | 238877 | 482708 | 0.166 | Improper consumption of alcohol | Lifestyles |
| Ever_smoke | 0.94 | 0.84 | 1.05 | 1.000 | 275748 | 482708 | 0.627 | Ever_smok | Lifestyles |
| Fish | 1.22 | 1.08 | 1.39 | 0.584 | 109979 | 482708 | 0.010 | Fish eaten above recommendation | Lifestyles |
| Fruit | 1.04 | 0.93 | 1.16 | 1.000 | 241110 | 482708 | 0.732 | Fruit eaten above recommendation | Lifestyles |
| Wholegrain | 1.17 | 1.05 | 1.31 | 1.000 | 159081 | 482708 | 0.193 | Wholegrain eaten above recommendation | Lifestyles |
| X1120 | 0.94 | 0.82 | 1.09 | 1.000 | 189839 | 482708 | 0.167 | Weekly usage of mobile phone in last 4 months | Lifestyles |
| X1120 | 0.96 | 0.80 | 1.15 | 1.000 | 81788 | 482708 | 0.167 | Weekly usage of mobile phone in last 4 months | Lifestyles |
| X1120 | 1.00 | 0.82 | 1.21 | 1.000 | 65829 | 482708 | 0.167 | Weekly usage of mobile phone in last 4 months | Lifestyles |
| X1120 | 0.94 | 0.69 | 1.29 | 1.000 | 18817 | 482708 | 0.167 | Weekly usage of mobile phone in last 4 months | Lifestyles |
| X1120 | 0.85 | 0.61 | 1.18 | 1.000 | 19280 | 482708 | 0.167 | Weekly usage of mobile phone in last 4 months | Lifestyles |
| X1130 | 0.75 | 0.60 | 0.93 | 1.000 | 43334 | 482708 | 0.993 | Hands -free device/speakerphone use with mobile phone in last 3 month | Lifestyles |
| X1130 | 0.46 | 0.30 | 0.71 | 0.156 | 16661 | 482708 | 0.993 | Hands -free device/speakerphone use with mobile phone in last 3 month | Lifestyles |
| X1130 | 0.79 | 0.50 | 1.23 | 1.000 | 9291 | 482708 | 0.993 | Hands -free device/speakerphone use with mobile phone in last 3 month | Lifestyles |
| X1130 | 0.96 | 0.68 | 1.36 | 1.000 | 12524 | 482708 | 0.993 | Hands -free device/speakerphone use with mobile phone in last 3 month | Lifestyles |
| Sleep_level | 1.77 | 1.58 | 1.98 | 9.66×10^-21^ | 128279 | 482708 | 0.232 | Sleep duration | Lifestyles |
| X1190 | 1.55 | 1.38 | 1.75 | 1.53×10^-10^ | 185608 | 482708 | 0.835 | Nap during day | Lifestyles |
| X1190 | 2.43 | 1.99 | 2.97 | 5.58×10^-16^ | 25754 | 482708 | 0.835 | Nap during day | Lifestyles |
| X1200 | 1.12 | 0.97 | 1.30 | 1.000 | 230078 | 482708 | 0.834 | Sleeplessness / insomnia | Lifestyles |
| X1200 | 1.43 | 1.22 | 1.68 | 0.003 | 136092 | 482708 | 0.834 | Sleeplessness / insomnia | Lifestyles |
| X1210 | 1.23 | 1.09 | 1.39 | 0.245 | 303371 | 482708 | 0.578 | Snoring | Lifestyles |
| X1220 | 1.49 | 1.31 | 1.69 | 2.70×10^-07^ | 102525 | 482708 | 0.016 | Daytime dozing / sleeping (narcolepsy) | Lifestyles |
| X1220 | 2.50 | 1.98 | 3.15 | 3.22×10^-12^ | 13546 | 482708 | 0.016 | Daytime dozing / sleeping (narcolepsy) | Lifestyles |
| X1220 | 11.96 | 1.73 | 82.69 | 1.000 | 37 | 482708 | 0.016 | Daytime dozing / sleeping (narcolepsy) | Lifestyles |
| X1269 | 1.83 | 1.53 | 2.19 | 1.23×10^-08^ | 28867 | 482708 | 0.027 | Exposure to tobacco smoke at home | Lifestyles |
| X1279 | 1.52 | 1.35 | 1.72 | 6.68×10^-09^ | 96917 | 482708 | 0.229 | Exposure to tobacco smoke outside home | Lifestyles |
| X2159 | 1.60 | 1.25 | 2.04 | 0.053 | 16666 | 482708 | 0.886 | Ever had sam×10-sex intercourse | Lifestyles |
| X22036 | 1.29 | 1.13 | 1.47 | 0.033 | 96166 | 482708 | 0.090 | Below moderate/vigorous/walking recommendation | Lifestyles |
| X6155_0 | 0.77 | 0.69 | 0.87 | 0.003 | 327075 | 482708 | 0.892 | No vitamin and mineral supplements | Lifestyles |
| X6155_1 | 1.86 | 1.40 | 2.48 | 0.006 | 9623 | 482708 | 0.352 | Vitamin A supplements | Lifestyles |
| X6155_2 | 1.81 | 1.47 | 2.23 | 8.64×10^-06^ | 20664 | 482708 | 0.535 | Vitamin B supplements | Lifestyles |
| X6155_3 | 1.25 | 1.04 | 1.49 | 1.000 | 42067 | 482708 | 0.323 | Vitamin C supplements | Lifestyles |
| X6155_4 | 1.50 | 1.19 | 1.89 | 0.202 | 19238 | 482708 | 0.075 | Vitamin D supplements | Lifestyles |
| X6155_5 | 1.38 | 1.05 | 1.81 | 1.000 | 14592 | 482708 | 0.328 | Vitamin E supplements | Lifestyles |
| X6155_6 | 1.65 | 1.23 | 2.21 | 0.236 | 10887 | 482708 | 0.076 | Folic acid or Folate (Vit B9) supplements | Lifestyles |
| X6155_7 | 1.15 | 1.01 | 1.31 | 1.000 | 105337 | 482708 | 0.573 | Multivitamins +/- minerals supplements | Lifestyles |
| X6179_0 | 0.98 | 0.87 | 1.09 | 1.000 | 273605 | 482708 | 0.066 | No mineral or other dietary supplements | Lifestyles |
| X6179_1 | 0.96 | 0.85 | 1.08 | 1.000 | 151296 | 482708 | 0.050 | Fish oil (including cod liver oil) supplements | Lifestyles |
| X6179_2 | 0.65 | 0.55 | 0.76 | 4.01×10^-05^ | 92077 | 482708 | 0.402 | Glucosamine supplements | Lifestyles |
| X6179_3 | 1.48 | 1.23 | 1.80 | 0.015 | 33619 | 482708 | 0.361 | Calcium supplements | Lifestyles |
| X6179_4 | 1.23 | 0.96 | 1.58 | 1.000 | 19830 | 482708 | 0.194 | Zinc supplements | Lifestyles |
| X6179_5 | 2.06 | 1.64 | 2.59 | 1.19×10^-07^ | 15959 | 482708 | 0.316 | Iron supplements | Lifestyles |
| X6179_6 | 1.34 | 0.99 | 1.83 | 1.000 | 11446 | 482708 | 0.898 | Selenium supplements | Lifestyles |
| Procesdd_meat | 1.21 | 1.08 | 1.36 | 0.477 | 291040 | 482708 | 0.855 | Processed meat intake reduced | Lifestyles |
| Healthy_diet_level | 0.79 | 0.70 | 0.90 | 0.157 | 136127 | 482708 | 0.110 | Healthy diet score | Lifestyles |
| Healthy_diet_level | 0.75 | 0.62 | 0.90 | 0.732 | 59145 | 482708 | 0.110 | Healthy diet score | Lifestyles |
| X1050 | 0.96 | 0.84 | 1.11 | 1.000 | 159627 | 482708 | 0.646 | Time spend outdoors in summer | Lifestyles |
| X1050 | 1.08 | 0.94 | 1.24 | 1.000 | 152491 | 482708 | 0.646 | Time spend outdoors in summer | Lifestyles |
| X1060 | 1.12 | 0.97 | 1.29 | 1.000 | 115403 | 482708 | 0.329 | Time spent outdoors in winter | Lifestyles |
| X1060 | 1.45 | 1.27 | 1.66 | 9.08×10^-06^ | 112332 | 482708 | 0.329 | Time spent outdoors in winter | Lifestyles |
| X1070 | 1.00 | 0.86 | 1.17 | 1.000 | 113790 | 482708 | 0.571 | Time spent watching television (TV) | Lifestyles |
| X1070 | 1.62 | 1.42 | 1.84 | 7.18×10^-11^ | 141552 | 482708 | 0.571 | Time spent watching television (TV) | Lifestyles |
| X1080 | 0.61 | 0.53 | 0.71 | 2.40×10^-09^ | 137832 | 482708 | 0.728 | Time spent using computer | Lifestyles |
| X1080 | 0.70 | 0.60 | 0.81 | 4.52×10^-04^ | 108874 | 482708 | 0.728 | Time spent using computer | Lifestyles |
| X1488 | 0.90 | 0.78 | 1.03 | 1.000 | 141534 | 482708 | 0.622 | Tea intake | Lifestyles |
| X1488 | 1.05 | 0.92 | 1.20 | 1.000 | 145309 | 482708 | 0.622 | Tea intake | Lifestyles |
| X1528 | 1.06 | 0.90 | 1.25 | 1.000 | 73065 | 482708 | 0.547 | Water intake | Lifestyles |
| X1528 | 1.23 | 1.08 | 1.39 | 0.427 | 140326 | 482708 | 0.547 | Water intake | Lifestyles |
| X2139 | 0.82 | 0.72 | 0.94 | 1.000 | 170308 | 482708 | 0.623 | Age first had sexual intercourse | Lifestyles |
| X2139 | 0.93 | 0.81 | 1.06 | 1.000 | 137656 | 482708 | 0.623 | Age first had sexual intercourse | Lifestyles |
| X2149 | 1.13 | 0.99 | 1.30 | 1.000 | 131599 | 482708 | 0.492 | Lifetime number of sexual partners | Lifestyles |
| X2149 | 1.24 | 1.08 | 1.42 | 0.566 | 148822 | 482708 | 0.492 | Lifetime number of sexual partners | Lifestyles |
| X22037 | 0.90 | 0.79 | 1.03 | 1.000 | 158789 | 482708 | 0.119 | MET minutes per week for walking | Lifestyles |
| X22037 | 0.90 | 0.79 | 1.03 | 1.000 | 150023 | 482708 | 0.119 | MET minutes per week for walking | Lifestyles |
| X22038 | 0.94 | 0.82 | 1.08 | 1.000 | 140336 | 482708 | 0.674 | MET minutes per week for moderate activity | Lifestyles |
| X22038 | 0.98 | 0.85 | 1.11 | 1.000 | 151800 | 482708 | 0.674 | MET minutes per week for moderate activity | Lifestyles |
| X22039 | 0.72 | 0.62 | 0.82 | 6.23×10^-04^ | 132270 | 482708 | 0.145 | MET minutes per week for vigorous activity | Lifestyles |
| X22039 | 0.72 | 0.63 | 0.82 | 2.24×10^-04^ | 150166 | 482708 | 0.145 | MET minutes per week for vigorous activity | Lifestyles |
| X1498 | 0.96 | 0.83 | 1.12 | 1.000 | 90659 | 482708 | 0.312 | Coffee intake | Lifestyles |
| X1498 | 0.94 | 0.83 | 1.07 | 1.000 | 153184 | 482708 | 0.312 | Coffee intake | Lifestyles |
| X22040 | 0.82 | 0.72 | 0.94 | 1.000 | 153639 | 482708 | 0.757 | Summed MET minutes per week for all activity | Lifestyles |
| X22040 | 0.86 | 0.75 | 0.98 | 1.000 | 157158 | 482708 | 0.757 | Summed MET minutes per week for all activity | Lifestyles |
| Vegetable | 0.75 | 0.66 | 0.86 | 0.010 | 165374 | 482708 | 0.291 | vegetable | Lifestyles |
| Vegetable | 0.86 | 0.75 | 0.98 | 1.000 | 150735 | 482708 | 0.291 | vegetable | Lifestyles |
| Red_meat | 1.09 | 0.92 | 1.30 | 1.000 | 53380 | 482708 | 0.139 | Red meat intake reudced | Lifestyles |
| White_bread_refined_cereals | 1.24 | 1.07 | 1.45 | 1.000 | 63227 | 482708 | 0.136 | White bread/refined cereals intake reduced | Lifestyles |
| X6160_0 | 1.17 | 1.04 | 1.31 | 1.000 | 146233 | 482708 | 0.954 | Leisure/social activities | Lifestyles |
| X6160_1 | 0.59 | 0.51 | 0.68 | 1.50×10^-11^ | 143204 | 482708 | 0.955 | Leisure/social activities | Lifestyles |
| X6160_2 | 0.97 | 0.86 | 1.11 | 1.000 | 127256 | 482708 | 0.562 | Leisure/social activities | Lifestyles |
| X6160_3 | 1.17 | 1.01 | 1.35 | 1.000 | 75112 | 482708 | 0.143 | Leisure/social activities | Lifestyles |
| X6160_4 | 1.45 | 1.21 | 1.74 | 0.019 | 34781 | 482708 | 0.532 | Leisure/social activities | Lifestyles |
| X6160_5 | 0.96 | 0.84 | 1.10 | 1.000 | 104612 | 482708 | 0.205 | Leisure/social activities | Lifestyles |
| X1031 | 0.23 | 0.18 | 0.28 | 2.09×10^-39^ | 474335 | 482708 | 0.046 | frequency of friend family visits | Lifestyle |
| X24006 | 1.23 | 1.06 | 1.42 | 1.000 | 161563 | 482708 | 0.346 | Particulate matter air pollution (pm2.5) | Local environment |
| X24006 | 1.78 | 1.55 | 2.04 | 6.14×10^-14^ | 159765 | 482708 | 0.346 | Particulate matter air pollution (pm2.5) | Local environment |
| X24008 | 1.27 | 1.11 | 1.46 | 0.146 | 162091 | 482708 | 0.093 | Particulate matter air pollution 2.5-10um | Local environment |
| X24008 | 1.22 | 1.06 | 1.40 | 1.000 | 154597 | 482708 | 0.093 | Particulate matter air pollution 2.5-10um | Local environment |
| X24020 | 1.06 | 0.93 | 1.22 | 1.000 | 160731 | 482708 | 0.661 | Average daytime sound level of noise pollution | Local environment |
| X24020 | 1.14 | 0.99 | 1.30 | 1.000 | 160632 | 482708 | 0.661 | Average daytime sound level of noise pollution | Local environment |
| X24021 | 1.07 | 0.93 | 1.23 | 1.000 | 158050 | 482708 | 0.739 | Average evening sound level of noise pollution | Local environment |
| X24021 | 1.11 | 0.97 | 1.27 | 1.000 | 158416 | 482708 | 0.739 | Average evening sound level of noise pollution | Local environment |
| X24022 | 1.07 | 0.93 | 1.23 | 1.000 | 158675 | 482708 | 0.739 | Average night-time sound level of noise pollution | Local environment |
| X24022 | 1.11 | 0.97 | 1.27 | 1.000 | 158115 | 482708 | 0.739 | Average night-time sound level of noise pollution | Local environment |
| X24500 | 0.85 | 0.75 | 0.97 | 1.000 | 161598 | 482708 | 0.504 | Greenspace percentage, buffer 1000m | Local environment |
| X24500 | 0.56 | 0.49 | 0.65 | 4.94×10^-13^ | 160948 | 482708 | 0.504 | Greenspace percentage, buffer 1000m | Local environment |
| X24501 | 1.03 | 0.90 | 1.18 | 1.000 | 163203 | 482708 | 0.299 | Domestic garden percentage, buffer 1000m | Local environment |
| X24501 | 0.97 | 0.85 | 1.11 | 1.000 | 159865 | 482708 | 0.299 | Domestic garden percentage, buffer 1000m | Local environment |
| X24502 | 0.88 | 0.77 | 1.01 | 1.000 | 160386 | 482708 | 0.610 | Water percentage, buffer 1000m | Local environment |
| X24502 | 1.06 | 0.93 | 1.21 | 1.000 | 160644 | 482708 | 0.610 | Water percentage, buffer 1000m | Local environment |
| X24503 | 1.02 | 0.90 | 1.16 | 1.000 | 162151 | 482708 | 0.718 | Greenspace percentage, buffer 300m | Local environment |
| X24503 | 0.69 | 0.60 | 0.79 | 7.59×10^-05^ | 160599 | 482708 | 0.718 | Greenspace percentage, buffer 300m | Local environment |
| X24504 | 0.92 | 0.81 | 1.05 | 1.000 | 162644 | 482708 | 0.383 | Domestic garden percentage, buffer 300m | Local environment |
| X24504 | 0.82 | 0.72 | 0.94 | 1.000 | 160537 | 482708 | 0.383 | Domestic garden percentage, buffer 300m | Local environment |
| X24505 | 0.82 | 0.71 | 0.94 | 1.000 | 159157 | 482708 | 0.919 | Water percentage, buffer 300m | Local environment |
| X24505 | 0.96 | 0.85 | 1.10 | 1.000 | 160009 | 482708 | 0.919 | Water percentage, buffer 300m | Local environment |
| X24506 | 0.80 | 0.71 | 0.91 | 0.162 | 160927 | 482708 | 0.028 | Natural environment percentage, buffer 1000m | Local environment |
| X24506 | 0.53 | 0.46 | 0.61 | 2.23×10^-16^ | 162845 | 482708 | 0.028 | Natural environment percentage, buffer 1000m | Local environment |
| X24507 | 0.94 | 0.83 | 1.07 | 1.000 | 159701 | 482708 | 0.778 | Natural environment percentage, buffer 300m | Local environment |
| X24507 | 0.68 | 0.59 | 0.78 | 2.07×10^-05^ | 161740 | 482708 | 0.778 | Natural environment percentage, buffer 300m | Local environment |
| NOx | 1.44 | 1.24 | 1.67 | 4.56×10^-04^ | 161280 | 482708 | 0.843 | Nitrogen oxides | Local environment |
| NOx | 1.96 | 1.70 | 2.26 | 2.20×10^-18^ | 158953 | 482708 | 0.843 | Nitrogen oxides | Local environment |
| X24023 | 1.06 | 0.93 | 1.22 | 1.000 | 158802 | 482708 | 0.739 | Average 16-hour sound level of noise pollution | Local environment |
| X24023 | 1.11 | 0.97 | 1.27 | 1.000 | 158310 | 482708 | 0.739 | Average 16-hour sound level of noise pollution | Local environment |
| X24024 | 1.06 | 0.93 | 1.22 | 1.000 | 158821 | 482708 | 0.739 | Average 24-hour sound level of noise pollution | Local environment |
| X24024 | 1.11 | 0.97 | 1.27 | 1.000 | 158334 | 482708 | 0.739 | Average 24-hour sound level of noise pollution | Local environment |
| PM10 | 1.38 | 1.19 | 1.59 | 0.005 | 161105 | 482708 | 0.009 | Particulate matter air pollution (pm10) | Local environment |
| PM10 | 1.77 | 1.54 | 2.03 | 2.77×10^-13^ | 159322 | 482708 | 0.009 | Particulate matter air pollution (pm10) | Local environment |
| Arthritis | 1.29 | 1.11 | 1.50 | 0.345 | 62533 | 482708 | 0.334 | Arthritis | Medical history |
| CHD | 1.59 | 1.29 | 1.95 | 0.003 | 26116 | 482708 | 0.461 | CHD | Medical history |
| COPD | 2.26 | 1.71 | 2.99 | 2.57×10^-06^ | 9407 | 482708 | 0.418 | COPD | Medical history |
| Diabetes | 1.90 | 1.56 | 2.31 | 2.77×10^-08^ | 24968 | 482708 | 0.062 | Diabetes | Medical history |
| Hear_loss | 0.88 | 0.78 | 0.99 | 1.000 | 184727 | 482708 | 0.552 | Hearing_loss | Medical history |
| Hepertension | 0.98 | 0.88 | 1.10 | 1.000 | 292288 | 482708 | 0.135 | Hypertension | Medical history |
| X6146 | 6.08 | 5.33 | 6.92 | <2×10^-16^ | 27829 | 482708 | 0.003 | Disability | Medical history |
| X6149_0 | 0.60 | 0.53 | 0.67 | 1.37×10^-17^ | 289683 | 482708 | 0.820 | Mouth/teeth dental problems | Medical history |
| X6149_1 | 1.11 | 0.93 | 1.32 | 1.000 | 48675 | 482708 | 0.332 | Mouth/teeth dental problems | Medical history |
| X6149_2 | 2.34 | 1.88 | 2.91 | 7.30×10^-12^ | 14621 | 482708 | 0.508 | Mouth/teeth dental problems | Medical history |
| X6149_3 | 1.24 | 1.07 | 1.45 | 1.000 | 64107 | 482708 | 0.355 | Mouth/teeth dental problems | Medical history |
| X6149_4 | 1.78 | 1.45 | 2.19 | 1.37×10^-05^ | 20831 | 482708 | 0.298 | Mouth/teeth dental problems | Medical history |
| X6149_5 | 2.10 | 1.74 | 2.55 | 1.24×10^-11^ | 21378 | 482708 | 0.453 | Mouth/teeth dental problems | Medical history |
| X6149_6 | 1.70 | 1.48 | 1.94 | 4.91×10^-12^ | 80425 | 482708 | 0.416 | Mouth/teeth dental problems | Medical history |
| X130176 | 0.31 | 0.20 | 0.48 | 3.01×10^-05^ | 25002 | 482708 | 0.518 | varicella [chickenpox] | Medical history |
| X130178 | 0.49 | 0.28 | 0.86 | 1.000 | 9168 | 482708 | 0.024 | zoster [herpes zoster] | Medical history |
| X130188 | 0.79 | 0.53 | 1.19 | 1.000 | 11437 | 482708 | 0.375 | viral warts | Medical history |
| X130226 | 1.01 | 0.77 | 1.32 | 1.000 | 20307 | 482708 | 0.195 | dermatophytosis | Medical history |
| X130622 | 1.33 | 0.95 | 1.86 | 1.000 | 10822 | 482708 | 0.926 | iron deficiency anaemia | Medical history |
| X130648 | 1.81 | 1.32 | 2.47 | 0.065 | 9275 | 482708 | 0.002 | other anaemias | Medical history |
| X130696 | 1.49 | 1.21 | 1.84 | 0.067 | 25373 | 482708 | 0.563 | other hypothyroidism | Medical history |
| X130792 | 1.25 | 0.91 | 1.70 | 1.000 | 13000 | 482708 | 0.561 | obesity | Medical history |
| X130814 | 1.34 | 1.16 | 1.55 | 0.030 | 72682 | 482708 | 0.266 | disorders of lipoprotein metabolism and other lipidaemias | Medical history |
| X131052 | 0.91 | 0.68 | 1.22 | 1.000 | 20509 | 482708 | 0.797 | migraine | Medical history |
| X131060 | 1.64 | 1.20 | 2.25 | 0.629 | 9212 | 482708 | 0.806 | sleep disorders) | Medical history |
| X131074 | 0.92 | 0.64 | 1.31 | 1.000 | 13055 | 482708 | 0.348 | mononeuropathies of upper limb | Medical history |
| X131128 | 0.84 | 0.52 | 1.36 | 1.000 | 7478 | 482708 | 0.222 | hordeolum and chalazion | Medical history |
| X131142 | 1.11 | 0.83 | 1.47 | 1.000 | 17154 | 482708 | 0.455 | conjunctivitis | Medical history |
| X131166 | 1.50 | 1.12 | 2.01 | 1.000 | 12237 | 482708 | 0.055 | other cataract | Medical history |
| X131222 | 0.92 | 0.64 | 1.31 | 1.000 | 12581 | 482708 | 0.660 | otitis externa | Medical history |
| X131224 | 0.78 | 0.55 | 1.10 | 1.000 | 15509 | 482708 | 0.852 | other disorders of external ear | Medical history |
| X131262 | 1.01 | 0.69 | 1.50 | 1.000 | 9765 | 482708 | 0.523 | otalgia and effusion of ear | Medical history |
| X131264 | 1.11 | 0.79 | 1.54 | 1.000 | 11932 | 482708 | 0.178 | other disorders of ear, not elsewhere classified | Medical history |
| X131350 | 1.27 | 0.86 | 1.86 | 1.000 | 8079 | 482708 | 0.352 | atrial fibrillation and flutter | Medical history |
| X131396 | 1.68 | 1.27 | 2.23 | 0.101 | 11590 | 482708 | 0.424 | phlebitis and thrombophlebitis | Medical history |
| X131402 | 0.91 | 0.66 | 1.26 | 1.000 | 15296 | 482708 | 0.653 | varicose veins of lower extremities | Medical history |
| X131404 | 1.36 | 1.03 | 1.81 | 1.000 | 13719 | 482708 | 0.278 | haemorrhoids | Medical history |
| X131426 | 0.86 | 0.63 | 1.18 | 1.000 | 17667 | 482708 | 0.879 | acute sinusitis | Medical history |
| X131428 | 0.79 | 0.57 | 1.11 | 1.000 | 17320 | 482708 | 0.695 | acute pharyngitis | Medical history |
| X131430 | 0.57 | 0.39 | 0.83 | 1.000 | 18093 | 482708 | 0.176 | acute tonsillitis | Medical history |
| X131436 | 1.10 | 0.89 | 1.37 | 1.000 | 30810 | 482708 | 0.482 | acute upper respiratory infections of multiple and unspecified sites | Medical history |
| X131442 | 0.68 | 0.40 | 1.15 | 1.000 | 7682 | 482708 | 0.347 | influenza, virus not identified | Medical history |
| X131456 | 1.41 | 1.03 | 1.92 | 1.000 | 11414 | 482708 | 0.338 | pneumonia, organism unspecified | Medical history |
| X131462 | 1.29 | 1.05 | 1.58 | 1.000 | 30260 | 482708 | 0.605 | unspecified acute lower respiratory infection | Medical history |
| X131464 | 0.90 | 0.74 | 1.10 | 1.000 | 42840 | 482708 | 0.949 | vasomotor and allergic rhinitis | Medical history |
| X131468 | 0.75 | 0.47 | 1.19 | 1.000 | 8810 | 482708 | 0.260 | chronic sinusitis | Medical history |
| X131472 | 1.15 | 0.83 | 1.60 | 1.000 | 11685 | 482708 | 0.073 | other disorders of nose and nasal sinuses | Medical history |
| X131494 | 1.51 | 1.30 | 1.75 | 1.55×10^-05^ | 56823 | 482708 | 0.842 | asthma | Medical history |
| X131584 | 1.32 | 1.10 | 1.59 | 1.000 | 35368 | 482708 | 0.130 | gastro -oesophageal reflux Medical history | Medical history |
| X131598 | 1.81 | 1.48 | 2.22 | 3.05×10^-06^ | 22223 | 482708 | 0.124 | gastritis and duodenitis | Medical history |
| X131600 | 1.61 | 1.18 | 2.19 | 0.722 | 9875 | 482708 | 0.767 | dyspepsia | Medical history |
| X131612 | 0.85 | 0.61 | 1.18 | 1.000 | 15387 | 482708 | 0.047 | inguinal hernia | Medical history |
| X131620 | 1.59 | 1.28 | 1.97 | 0.007 | 22077 | 482708 | 0.666 | diaphragmatic hernia | Medical history |
| X131630 | 2.28 | 1.82 | 2.87 | 3.91×10^-10^ | 13708 | 482708 | 0.158 | other non -infective gastro -enteritis and colitis | Medical history |
| X131636 | 1.33 | 1.02 | 1.74 | 1.000 | 15771 | 482708 | 0.653 | diverticular Medical history of intestine | Medical history |
| X131638 | 1.39 | 1.12 | 1.72 | 0.934 | 24937 | 482708 | 0.547 | irritable bowel syndrome | Medical history |
| X131640 | 2.41 | 1.81 | 3.21 | 4.53×10^-07^ | 7976 | 482708 | 0.750 | other functional intestinal disorders | Medical history |
| X131646 | 1.39 | 1.10 | 1.76 | 1.000 | 20584 | 482708 | 0.907 | other medical historys of anus and rectum | Medical history |
| X131650 | 0.79 | 0.58 | 1.06 | 1.000 | 20585 | 482708 | 0.403 | haemorrhoids and perianal venous thrombosis | Medical history |
| X131674 | 0.94 | 0.68 | 1.29 | 1.000 | 15797 | 482708 | 0.174 | cholelithiasis | Medical history |
| X131702 | 1.51 | 1.14 | 1.99 | 1.000 | 13447 | 482708 | 0.468 | cellulitis | Medical history |
| X131708 | 1.24 | 0.83 | 1.86 | 1.000 | 7392 | 482708 | 0.210 | other local infections of skin and subcutaneous tissue | Medical history |
| X131720 | 0.78 | 0.50 | 1.21 | 1.000 | 9811 | 482708 | 0.310 | atopic dermatitis | Medical history |
| X131740 | 0.88 | 0.70 | 1.11 | 1.000 | 33305 | 482708 | 0.940 | other dermatitis | Medical history |
| X131742 | 0.87 | 0.58 | 1.29 | 1.000 | 10731 | 482708 | 0.817 | psoriasis | Medical history |
| X131768 | 0.77 | 0.46 | 1.28 | 1.000 | 7070 | 482708 | 0.454 | skin changes due to chronic exposure to nonionising radiation | Medical history |
| X131794 | 0.96 | 0.63 | 1.45 | 1.000 | 9079 | 482708 | 0.061 | follicular cysts of skin and subcutaneous tissue | Medical history |
| X131806 | 0.70 | 0.48 | 1.04 | 1.000 | 13483 | 482708 | 0.380 | seborrhoeic keratosis | Medical history |
| X131836 | 0.78 | 0.58 | 1.05 | 1.000 | 22396 | 482708 | 0.994 | other disorders of skin and subcutaneous tissue, not elsewhere classified | Medical history |
| X131858 | 0.92 | 0.62 | 1.37 | 1.000 | 10327 | 482708 | 0.182 | gout | Medical history |
| X131864 | 1.18 | 0.86 | 1.62 | 1.000 | 12743 | 482708 | 0.263 | other arthritis | Medical history |
| X131872 | 1.52 | 1.16 | 2.00 | 0.686 | 13727 | 482708 | 0.733 | gonarthrosis [arthrosis of knee] | Medical history |
| X131876 | 1.20 | 1.01 | 1.42 | 1.000 | 50678 | 482708 | 0.412 | other arthrosis | Medical history |
| X131878 | 1.20 | 0.85 | 1.69 | 1.000 | 10669 | 482708 | 0.602 | acquired deformities of fingers and toes | Medical history |
| X131884 | 0.86 | 0.58 | 1.30 | 1.000 | 10492 | 482708 | 0.096 | internal derangement of knee | Medical history |
| X131888 | 0.98 | 0.82 | 1.17 | 1.000 | 52660 | 482708 | 0.563 | other joint disorders, not elsewhere classified | Medical history |
| X131916 | 1.43 | 1.11 | 1.85 | 1.000 | 16436 | 482708 | 0.532 | spondylosis | Medical history |
| X131924 | 0.84 | 0.60 | 1.17 | 1.000 | 15650 | 482708 | 0.174 | other intervertebral disk disorders | Medical history |
| X131928 | 1.19 | 1.00 | 1.41 | 1.000 | 46115 | 482708 | 0.462 | dorsalgia | Medical history |
| X131938 | 0.93 | 0.61 | 1.40 | 1.000 | 9483 | 482708 | 0.144 | synovitis and tenosynovitis | Medical history |
| X131946 | 0.74 | 0.45 | 1.22 | 1.000 | 8110 | 482708 | 0.298 | soft tissue disorders related to use, overuse and pressure | Medical history |
| X131950 | 0.77 | 0.51 | 1.17 | 1.000 | 10997 | 482708 | 0.743 | fibroblastic disorders | Medical history |
| X131954 | 0.82 | 0.58 | 1.14 | 1.000 | 15997 | 482708 | 0.656 | shoulder lesions | Medical history |
| X131958 | 0.77 | 0.57 | 1.05 | 1.000 | 21359 | 482708 | 0.261 | other enthesopathies | Medical history |
| X131960 | 1.14 | 0.95 | 1.36 | 1.000 | 44503 | 482708 | 0.809 | other soft tissue disorders, not elsewhere classified | Medical history |
| X131964 | 1.73 | 1.27 | 2.36 | 0.156 | 9477 | 482708 | 0.204 | osteoporosis without pathological fracture | Medical history |
| X132054 | 1.29 | 0.93 | 1.79 | 1.000 | 11071 | 482708 | 0.985 | cystitis | Medical history |
| X132070 | 1.38 | 1.12 | 1.70 | 0.877 | 27229 | 482708 | 0.009 | other disorders of urinary system | Medical history |
| X132072 | 0.97 | 0.69 | 1.35 | 1.000 | 13388 | 482708 | 0.828 | hyperplasia of prostate | Medical history |
| X132096 | 0.71 | 0.45 | 1.11 | 1.000 | 10623 | 482708 | 0.663 | benign mammary dysplasia | Medical history |
| X132102 | 0.93 | 0.64 | 1.34 | 1.000 | 12634 | 482708 | 0.436 | unspecified lump in breast | Medical history |
| X132104 | 0.64 | 0.38 | 1.07 | 1.000 | 9019 | 482708 | 0.651 | other disorders of breast | Medical history |
| X132124 | 1.27 | 0.91 | 1.78 | 1.000 | 10699 | 482708 | 0.664 | female genital prolapse | Medical history |
| X132128 | 1.40 | 0.99 | 1.99 | 1.000 | 9387 | 482708 | 0.170 | noninflammatory disorders of ovary, fallopian tube and broad ligament | Medical history |
| X132130 | 0.82 | 0.55 | 1.22 | 1.000 | 11563 | 482708 | 0.774 | polyp of female genital tract | Medical history |
| X132140 | 0.97 | 0.63 | 1.50 | 1.000 | 8374 | 482708 | 0.795 | other noninflammatory disorders of vagina | Medical history |
| X132146 | 0.83 | 0.62 | 1.10 | 1.000 | 23809 | 482708 | 0.076 | excessive, frequent and irregular menstruation | Medical history |
| X132148 | 0.77 | 0.47 | 1.27 | 1.000 | 8188 | 482708 | 0.491 | other abnormal uterine and vaginal bleeding | Medical history |
| X132150 | 1.20 | 0.83 | 1.73 | 1.000 | 10138 | 482708 | 0.247 | pain and other conditions associated with female genital organs and menstrual cycle | Medical history |
| X132152 | 1.08 | 0.94 | 1.23 | 1.000 | 312133 | 482708 | 0.688 | menopausal and other perimenopausal disorders | Medical history |
| X132276 | 0.86 | 0.60 | 1.23 | 1.000 | 14682 | 482708 | 0.318 | single spontaneous delivery | Medical history |
| Stroke | 2.33 | 1.73 | 3.13 | 6.47×10^-06^ | 7804 | 482708 | 0.650 | stroke | Medical history |
| TBI | 1.05 | 0.93 | 1.20 | 1.000 | 126627 | 482708 | 0.010 | Traumatic brain injury | Medical history |
| Vision_impairment | 0.89 | 0.78 | 1.01 | 1.000 | 134298 | 482708 | 0.204 | Vision_impairment | Medical history |
| Dementia | 7.52 | 2.42 | 23.36 | 0.147 | 185 | 482708 | 0.443 | dementia | Mental health |
| Intellectual disability | 35.15 | 11.23 | 110.08 | 2.96×10^-07^ | 39 | 482708 | 0.677 | Intellectual disability | Mental health |
| Others personality disorders | 3.76 | 0.94 | 15.08 | 1.000 | 209 | 482708 | 0.324 | others disorders of adult personality and behaviour | Mental health |
| Others behavioural syndromes | 22.29 | 3.05 | 162.95 | 0.671 | 19 | 482708 | 0.960 | others behavioural syndromes associated with physiological disturbances and physical factors | Mental health |
| Others substance abuse | 5.98 | 0.84 | 42.46 | 1.000 | 66 | 482708 | 0.949 | others mental and behavioural disorders due to psychoactive substance use | Mental health |
| Others Mood disorders | 5.09 | 2.54 | 10.21 | 0.001 | 634 | 482708 | 0.514 | others mood [affective] disorders | Mental health |
| Other organic mental disorders | 20.63 | 14.93 | 28.51 | 1.40×10^-72^ | 718 | 482708 | 0.214 | other organic,including symptomatic, mental disorders | Mental health |
| Other mental disorders | 12.02 | 6.95 | 20.77 | 1.58×10^-16^ | 405 | 482708 | 0.822 | other mental and behavioural disorders | Mental health |
| Psychological development disorders | 23.73 | 13.67 | 41.20 | 6.93×10^-27^ | 237 | 482708 | 0.235 | Disorders of psychological development | Mental health |
| X130854 | 5.80 | 4.41 | 7.63 | 1.11×10^-33^ | 3929 | 482708 | 0.012 | Mental and behavioural disorders due to use of alcohol | Mental health |
| X130856 | 15.93 | 6.57 | 38.65 | 2.78×10^-07^ | 139 | 482708 | 0.387 | Mental and behavioural disorders due to use of opioids | Mental health |
| X130858 | 20.05 | 7.50 | 53.59 | 6.80×10^-07^ | 88 | 482708 | 0.728 | Mental and behavioural disorders due to use of cannabinoids | Mental health |
| X130860 | 2.13 | 0.30 | 15.17 | 1.000 | 173 | 482708 | 0.592 | Mental and behavioural disorders due to use of sedatives or hypnotics | Mental health |
| X130868 | 1.69 | 1.36 | 2.09 | 5.87×10^-04^ | 21098 | 482708 | 0.651 | Mental and behavioural disorders due to use of tobacco | Mental health |
| X130872 | 15.62 | 7.36 | 33.14 | 2.36×10^-10^ | 179 | 482708 | 0.066 | Mental and behavioural disorders due to multiple drug use and use of other psychoactive substances | Mental health |
| X130890 | 33.14 | 21.16 | 51.90 | 2.50×10^-50^ | 217 | 482708 | 0.380 | Manic episode | Mental health |
| X130892 | 32.91 | 27.07 | 40.01 | <2×10^-16^ | 1394 | 482708 | 0.131 | Bipolar affective disorder | Mental health |
| X130894 | 4.95 | 4.38 | 5.58 | <2×10^-16^ | 39387 | 482708 | 0.000 | Depressive episode | Mental health |
| X130896 | 8.85 | 6.76 | 11.58 | 3.19×10^-54^ | 2509 | 482708 | 0.005 | Recurrent depressive disorder | Mental health |
| X130898 | 5.32 | 2.76 | 10.28 | 1.91×10^-04^ | 639 | 482708 | 0.284 | Persistent mood [affective] disorders | Mental health |
| X130904 | 2.33 | 1.21 | 4.50 | 1.000 | 1504 | 482708 | 0.582 | Phobic anxiety disorders | Mental health |
| X130906 | 3.55 | 3.00 | 4.22 | 1.55×10^-45^ | 17341 | 482708 | 0.001 | Other anxiety disorders | Mental health |
| X130908 | 11.05 | 6.84 | 17.85 | 2.71×10^-20^ | 579 | 482708 | 0.901 | Obsessiv×10-compulsive disorder | Mental health |
| X130910 | 1.78 | 1.33 | 2.39 | 0.037 | 9981 | 482708 | 0.073 | Reaction to severe stress, and adjustment disorders | Mental health |
| X130912 | 18.48 | 8.80 | 38.82 | 4.07×10^-12^ | 153 | 482708 | 0.287 | Dissociative [conversion] disorders | Mental health |
| X130914 | 2.47 | 1.40 | 4.36 | 0.558 | 1826 | 482708 | 0.174 | Somatoform disorders | Mental health |
| X130916 | 14.58 | 10.41 | 20.42 | 3.06×10^-52^ | 899 | 482708 | 0.956 | Other neurotic disorders | Mental health |
| X130918 | 5.19 | 2.77 | 9.70 | 7.71×10^-05^ | 803 | 482708 | 0.046 | Eating disorders | Mental health |
| X130920 | 2.03 | 0.51 | 8.15 | 1.000 | 359 | 482708 | 0.071 | Nonorganic sleep disorders | Mental health |
| X130922 | 1.97 | 0.94 | 4.14 | 1.000 | 1342 | 482708 | 0.026 | Sexual dysfunction, not caused by organic disorder or disease | Mental health |
| X130924 | 4.26 | 2.51 | 7.24 | 2.49×10^-05^ | 1293 | 482708 | 0.759 | Mental and behavioural disorders associated with the puerperium, not elsewhere classified | Mental health |
| X130932 | 20.10 | 12.44 | 32.47 | 4.22×10^-32^ | 345 | 482708 | 0.596 | Specific personality disorders | Mental health |
| X130944 | 1.08 | 0.15 | 7.68 | 1.000 | 353 | 482708 | 0.100 | Psychological and behavioural disorders associated with sexual development and orientation | Mental health |
| X1920 | 1.86 | 1.66 | 2.08 | 4.65×10^-25^ | 219251 | 482708 | 0.008 | Mood swings | Mental health |
| X1930 | 1.53 | 1.37 | 1.71 | 1.70×10^-11^ | 206348 | 482708 | 0.098 | Miserableness | Mental health |
| X1940 | 1.21 | 1.08 | 1.36 | 0.448 | 136085 | 482708 | 0.570 | Irritability | Mental health |
| X1950 | 1.39 | 1.24 | 1.56 | 7.48×10^-06^ | 266796 | 482708 | 0.734 | Sensitivity / hurt feelings | Mental health |
| X1960 | 1.61 | 1.45 | 1.80 | 4.53×10^-15^ | 196454 | 482708 | 0.031 | Fed-up feelings | Mental health |
| X1970 | 2.46 | 2.20 | 2.75 | 1.57×10^-53^ | 114438 | 482708 | 0.206 | Nervous feelings | Mental health |
| X1980 | 1.79 | 1.59 | 2.02 | 1.01×10^-18^ | 272797 | 482708 | 0.545 | Worrier / anxious feelings | Mental health |
| X1990 | 2.40 | 2.13 | 2.69 | 8.67×10^-47^ | 86455 | 482708 | 0.904 | Tense / 'highly strung | Mental health |
| X2000 | 1.32 | 1.18 | 1.48 | 2.40×10^-04^ | 228876 | 482708 | 0.978 | Worry too long after embarrassment | Mental health |
| X2010 | 2.69 | 2.41 | 3.00 | 4.51×10^-66^ | 103749 | 482708 | 0.009 | Suffer from 'nerves' | Mental health |
| X2020 | 2.53 | 2.25 | 2.84 | 9.57×10^-53^ | 89862 | 482708 | 0.012 | Loneliness, isolation | Mental health |
| X2030 | 1.54 | 1.37 | 1.73 | 5.91×10^-11^ | 139695 | 482708 | 0.100 | Guilty feelings | Mental health |
| X2110 | 1.42 | 1.26 | 1.60 | 1.04×10^-06^ | 124960 | 482708 | 0.701 | Able to confide | Mental health |
| Depression | 2.48 | 2.22 | 2.77 | 4.97×10^-55^ | 116086 | 482708 | 0.015 | frequency of depressed mood in last 2 weeks | Mental health |
| X6145 | 1.53 | 1.37 | 1.70 | 1.56×10^-11^ | 216299 | 482708 | 0.021 | illness injury bereavement stress in last 2 years | Mental health |
| X2090 | 3.46 | 3.08 | 3.89 | 5.20×10^-95^ | 163336 | 482708 | 0.000 | seen doctor gp for nerves anxiety tension or depression | Mental health |
| X2100 | 6.64 | 5.95 | 7.42 | <2×10^-16^ | 405 | 482708 | 0.000 | seen a psychiatrist for nerves anxiety tension or depression | Mental health |
| X2070 | 2.27 | 2.03 | 2.54 | 2.09×10^-45^ | 130564 | 482708 | 0.011 | frequency of tenseness restlessness in last 2 week | Mental health |
| X2080 | 1.69 | 1.51 | 1.90 | 2.35×10^-16^ | 257881 | 482708 | 0.171 | frequency of tiredness lethargy in last 2 weeks | Mental health |
| X2060 | 2.44 | 2.18 | 2.73 | 2.47×10^-52^ | 104866 | 482708 | 0.020 | frequency of unenthusiasm disinterest in last 2 weeks | Mental health |
| Arm_fat_  percentage | 1.30 | 1.12 | 1.52 | 0.261 | 161719 | 482708 | 0.011 | Arm fat percentage | Physical measures |
| Arm_fat_  percentage | 1.80 | 1.47 | 2.20 | 3.71×10^-06^ | 159093 | 482708 | 0.011 | Arm fat percentage | Physical measures |
| X4079 | 0.83 | 0.72 | 0.95 | 1.000 | 157718 | 482708 | 0.033 | Diastolic blood pressure | Physical measures |
| X4079 | 0.97 | 0.85 | 1.11 | 1.000 | 161052 | 482708 | 0.033 | Diastolic blood pressure | Physical measures |
| Hand_grip_strength | 0.62 | 0.53 | 0.72 | 1.30×10^-07^ | 163277 | 482708 | 0.531 | Hand grip strength | Physical measures |
| Hand_grip_strength | 0.35 | 0.29 | 0.44 | 3.53×10^-20^ | 156918 | 482708 | 0.531 | Hand grip strength | Physical measures |
| Leg_fat_percentage | 1.23 | 1.03 | 1.47 | 1.000 | 162224 | 482708 | 0.068 | Leg fat percentage | Physical measures |
| Leg_fat_percentage | 1.66 | 1.31 | 2.11 | 0.010 | 158697 | 482708 | 0.068 | Leg fat percentage | Physical measures |
| Lung_function | 0.61 | 0.53 | 0.69 | 3.52×10^-11^ | 160798 | 482708 | 0.517 | Lung_function | Physical measures |
| Lung_function | 0.40 | 0.34 | 0.47 | 5.05×10^-26^ | 160706 | 482708 | 0.517 | Lung_function | Physical measures |
| X23099 | 1.22 | 1.05 | 1.43 | 1.000 | 162405 | 482708 | 0.017 | Body fat percentage | Physical measures |
| X23099 | 1.55 | 1.28 | 1.88 | 0.002 | 158599 | 482708 | 0.017 | Body fat percentage | Physical measures |
| X23127 | 1.14 | 0.99 | 1.31 | 1.000 | 163010 | 482708 | 0.115 | Trunk fat percentage | Physical measures |
| X23127 | 1.43 | 1.23 | 1.67 | 0.001 | 158090 | 482708 | 0.115 | Trunk fat percentage | Physical measures |
| X23105 | 1.12 | 0.96 | 1.31 | 1.000 | 160132 | 482708 | 0.114 | Basal metabolic rate | Physical measures |
| X23105 | 1.08 | 0.86 | 1.35 | 1.000 | 161112 | 482708 | 0.114 | Basal metabolic rate | Physical measures |
| X4080 | 0.84 | 0.73 | 0.97 | 1.000 | 161774 | 482708 | 0.158 | Systolic blood pressure | Physical measures |
| X4080 | 0.89 | 0.77 | 1.03 | 1.000 | 158859 | 482708 | 0.158 | Systolic blood pressure | Physical measures |
| X6138 | 1.50 | 1.34 | 1.68 | 1.01×10^-09^ | 254119 | 482708 | 0.627 | Lower education | SES |
| X6142 | 2.07 | 1.65 | 2.61 | 2.04×10^-07^ | 15425 | 482708 | 0.626 | Unemployed | SES |
| X738 | 0.44 | 0.38 | 0.50 | 3.41×10^-29^ | 124943 | 482708 | 0.107 | Average total household income before tax | SES |
| X738 | 0.28 | 0.24 | 0.33 | 5.09×10^-48^ | 122841 | 482708 | 0.107 | Average total household income before tax | SES |
| X738 | 0.19 | 0.15 | 0.24 | 1.62×10^-48^ | 92866 | 482708 | 0.107 | Average total household income before tax | SES |
| X738 | 0.14 | 0.09 | 0.22 | 9.80×10^-16^ | 24150 | 482708 | 0.107 | Average total household income before tax | SES |
| X22189 | 1.28 | 1.09 | 1.50 | 0.890 | 160287 | 482708 | 0.035 | Townsend deprivation index at recruitment | SES |
| X22189 | 2.65 | 2.30 | 3.06 | 5.38×10^-39^ | 159119 | 482708 | 0.035 | Townsend deprivation index at recruitment | SES |
|  | | | | | | | | | |

| **Supplementary Table 8. Associations between 232 risk factors and late onset schizophrenia in EWAS (by age and length of follow up).** Blue cells indicate P.adjust<0.05; P.adjust, after Bonferroni correction. | | | | | | | | | | | | |
| --- | --- | --- | --- | --- | --- | --- | --- | --- | --- | --- | --- | --- |
|  | **Whole sample** | | **Age** | | | | **Follow-up time** | | | | **Exposures** | **Category** |
|  |  |  | **<65 years** | | **≥65 years** | | **≥ 5 years** | | **≥ 10 years** | |  |  |
| **FieldID** | **HR** | **P.adjust** | **HR** | **P.adjust** | **HR** | **P.adjust** | **HR** | **P.adjust** | **HR** | **P.adjust** |  |  |
| Alcohol_level | 1.53 | 1.49×10^-10^ | 1.57 | 1.58×10^-08^ | 1.39 | 1.000 | 1.47 | 7.21×10^-06^ | 1.23 | 1.000 | Improper consumption of alcohol | Lifestyles |
| Ever_smoke | 0.94 | 1.000 | 0.95 | 1.000 | 0.93 | 1.000 | 0.95 | 1.000 | 0.93 | 1.000 | Ever_smok | Lifestyles |
| Fish | 1.22 | 0.584 | 1.23 | 1.000 | 1.21 | 1.000 | 1.19 | 1.000 | 1.01 | 1.000 | Fish eaten above recommendation | Lifestyles |
| Fruit | 1.04 | 1.000 | 1.03 | 1.000 | 1.06 | 1.000 | 1.07 | 1.000 | 1.13 | 1.000 | Fruit eaten above recommendation | Lifestyles |
| Wholegrain | 1.17 | 1.000 | 1.22 | 0.980 | 1.02 | 1.000 | 1.12 | 1.000 | 1.08 | 1.000 | Wholegrain eaten above recommendation | Lifestyles |
| X1120 | 0.94 | 1.000 | 0.90 | 1.000 | 1.03 | 1.000 | 1.05 | 1.000 | 1.11 | 1.000 | Weekly usage of mobile phone in last 4 months | Lifestyles |
| X1120 | 0.96 | 1.000 | 0.83 | 1.000 | 1.46 | 1.000 | 1.03 | 1.000 | 1.25 | 1.000 | Weekly usage of mobile phone in last 4 months | Lifestyles |
| X1120 | 1.00 | 1.000 | 0.85 | 1.000 | 1.73 | 1.000 | 1.04 | 1.000 | 1.40 | 1.000 | Weekly usage of mobile phone in last 4 months | Lifestyles |
| X1120 | 0.94 | 1.000 | 0.78 | 1.000 | 2.08 | 1.000 | 1.05 | 1.000 | 1.04 | 1.000 | Weekly usage of mobile phone in last 4 months | Lifestyles |
| X1120 | 0.85 | 1.000 | 0.76 | 1.000 | 1.07 | 1.000 | 0.89 | 1.000 | 0.89 | 1.000 | Weekly usage of mobile phone in last 4 months | Lifestyles |
| X1130 | 0.75 | 1.000 | 0.68 | 0.580 | 1.16 | 1.000 | 0.80 | 1.000 | 0.78 | 1.000 | Hands -free device/speakerphone use with mobile phone in last 3 month | Lifestyles |
| X1130 | 0.46 | 0.156 | 0.39 | 0.057 | 1.02 | 1.000 | 0.45 | 0.905 | 0.59 | 1.000 | Hands -free device/speakerphone use with mobile phone in last 3 month | Lifestyles |
| X1130 | 0.79 | 1.000 | 0.63 | 1.000 | 2.29 | 1.000 | 0.86 | 1.000 | 0.67 | 1.000 | Hands -free device/speakerphone use with mobile phone in last 3 month | Lifestyles |
| X1130 | 0.96 | 1.000 | 0.86 | 1.000 | 1.52 | 1.000 | 1.00 | 1.000 | 0.96 | 1.000 | Hands -free device/speakerphone use with mobile phone in last 3 month | Lifestyles |
| Sleep_level | 1.77 | 9.66×10^-21^ | 1.86 | 2.01×10^-18^ | 1.54 | 0.068 | 1.72 | 5.38×10^-13^ | 1.67 | 1.42×10^-04^ | Sleep duration | Lifestyles |
| X1190 | 1.55 | 1.53×10^-10^ | 1.66 | 1.13×10^-10^ | 1.21 | 1.000 | 1.55 | 3.24×10^-07^ | 1.41 | 0.363 | Nap during day | Lifestyles |
| X1190 | 2.43 | 5.58×10^-16^ | 3.04 | 1.12×10^-19^ | 1.30 | 1.000 | 2.32 | 7.34×10^-10^ | 1.97 | 0.068 | Nap during day | Lifestyles |
| X1200 | 1.12 | 1.000 | 1.08 | 1.000 | 1.25 | 1.000 | 1.05 | 1.000 | 1.22 | 1.000 | Sleeplessness / insomnia | Lifestyles |
| X1200 | 1.43 | 0.003 | 1.55 | 7.48×10^-04^ | 1.19 | 1.000 | 1.38 | 0.195 | 1.40 | 1.000 | Sleeplessness / insomnia | Lifestyles |
| X1210 | 1.23 | 0.245 | 1.18 | 1.000 | 1.32 | 1.000 | 1.23 | 1.000 | 1.27 | 1.000 | Snoring | Lifestyles |
| X1220 | 1.49 | 2.70×10^-07^ | 1.63 | 2.18×10^-08^ | - | - | 1.37 | 0.016 | 1.06 | 1.000 | Daytime dozing / sleeping (narcolepsy) | Lifestyles |
| X1220 | 2.50 | 3.22×10^-12^ | 2.87 | 8.67×10^-13^ | - | - | 2.35 | 4.68×10^-07^ | 1.30 | 1.000 | Daytime dozing / sleeping (narcolepsy) | Lifestyles |
| X1220 | 11.96 | 1.000 | 18.16 | 0.912 | - | - | 16.08 | 1.000 | 33.13 | 0.160 | Daytime dozing / sleeping (narcolepsy) | Lifestyles |
| X1269 | 1.83 | 1.23×10^-08^ | 2.05 | 2.67×10^-10^ | 1.15 | 1.000 | 1.60 | 0.012 | 1.49 | 1.000 | Exposure to tobacco smoke at home | Lifestyles |
| X1279 | 1.52 | 6.68×10^-09^ | 1.57 | 9.22×10^-08^ | 1.37 | 1.000 | 1.60 | 5.21×10^-08^ | 1.79 | 1.69×10^-05^ | Exposure to tobacco smoke outside home | Lifestyles |
| X2159 | 1.60 | 0.053 | 1.71 | 0.011 | 0.83 | 1.000 | 1.58 | 0.640 | 1.73 | 1.000 | Ever had sam×10-sex intercourse | Lifestyles |
| X22036 | 1.29 | 0.033 | 1.22 | 1.000 | 1.59 | 0.098 | 1.23 | 1.000 | 1.17 | 1.000 | Below moderate/vigorous/walking recommendation | Lifestyles |
| X6155_0 | 0.77 | 0.003 | 0.76 | 0.011 | 0.82 | 1.000 | 0.79 | 0.210 | 0.76 | 1.000 | No vitamin and mineral supplements | Lifestyles |
| X6155_1 | 1.86 | 0.006 | 1.68 | 1.000 | 2.21 | 0.228 | 1.67 | 1.000 | 1.36 | 1.000 | Vitamin A supplements | Lifestyles |
| X6155_2 | 1.81 | 8.64×10^-06^ | 1.96 | 7.16×10^-06^ | 1.40 | 1.000 | 1.61 | 0.087 | 1.77 | 0.772 | Vitamin B supplements | Lifestyles |
| X6155_3 | 1.25 | 1.000 | 1.18 | 1.000 | 1.42 | 1.000 | 1.14 | 1.000 | 1.17 | 1.000 | Vitamin C supplements | Lifestyles |
| X6155_4 | 1.50 | 0.202 | 1.53 | 0.881 | 1.42 | 1.000 | 1.29 | 1.000 | 1.01 | 1.000 | Vitamin D supplements | Lifestyles |
| X6155_5 | 1.38 | 1.000 | 1.47 | 1.000 | 1.17 | 1.000 | 1.23 | 1.000 | 0.96 | 1.000 | Vitamin E supplements | Lifestyles |
| X6155_6 | 1.65 | 0.236 | 1.61 | 1.000 | 1.70 | 1.000 | 1.45 | 1.000 | 1.07 | 1.000 | Folic acid or Folate (Vit B9) supplements | Lifestyles |
| X6155_7 | 1.15 | 1.000 | 1.19 | 1.000 | 1.06 | 1.000 | 1.12 | 1.000 | 1.26 | 1.000 | Multivitamins +/- minerals supplements | Lifestyles |
| X6179_0 | 0.98 | 1.000 | 0.95 | 1.000 | 1.04 | 1.000 | 0.97 | 1.000 | 0.92 | 1.000 | No mineral or other dietary supplements | Lifestyles |
| X6179_1 | 0.96 | 1.000 | 1.03 | 1.000 | 0.81 | 1.000 | 0.96 | 1.000 | 1.02 | 1.000 | Fish oil (including cod liver oil) supplements | Lifestyles |
| X6179_2 | 0.65 | 4.01×10^-05^ | 0.65 | 0.006 | 0.65 | 0.787 | 0.66 | 0.003 | 0.67 | 1.000 | Glucosamine supplements | Lifestyles |
| X6179_3 | 1.48 | 0.015 | 1.46 | 0.419 | 1.54 | 1.000 | 1.38 | 1.000 | 1.33 | 1.000 | Calcium supplements | Lifestyles |
| X6179_4 | 1.23 | 1.000 | 1.13 | 1.000 | 1.53 | 1.000 | 1.10 | 1.000 | 1.07 | 1.000 | Zinc supplements | Lifestyles |
| X6179_5 | 2.06 | 1.19×10^-07^ | 1.82 | 0.002 | 2.96 | 2.73×10^-04^ | 2.10 | 1.56×10^-05^ | 1.76 | 1.000 | Iron supplements | Lifestyles |
| X6179_6 | 1.34 | 1.000 | 1.27 | 1.000 | 1.50 | 1.000 | 1.45 | 1.000 | 1.23 | 1.000 | Selenium supplements | Lifestyles |
| Procesdd_meat | 1.21 | 0.477 | 1.21 | 1.000 | 1.18 | 1.000 | 1.25 | 0.502 | 1.27 | 1.000 | Processed meat intake reduced | Lifestyles |
| Healthy_diet_level | 0.79 | 0.157 | 0.76 | 0.198 | 0.89 | 1.000 | 0.85 | 1.000 | 0.86 | 1.000 | Healthy diet score | Lifestyles |
| Healthy_diet_level | 0.75 | 0.732 | 0.80 | 1.000 | 0.64 | 1.000 | 0.77 | 1.000 | 0.82 | 1.000 | Healthy diet score | Lifestyles |
| X1050 | 0.96 | 1.000 | 1.01 | 1.000 | 0.76 | 1.000 | 0.96 | 1.000 | 1.06 | 1.000 | Time spend outdoors in summer | Lifestyles |
| X1050 | 1.08 | 1.000 | 1.20 | 1.000 | 0.72 | 1.000 | 1.05 | 1.000 | 1.16 | 1.000 | Time spend outdoors in summer | Lifestyles |
| X1060 | 1.12 | 1.000 | 1.16 | 1.000 | 0.97 | 1.000 | 1.14 | 1.000 | 1.09 | 1.000 | Time spent outdoors in winter | Lifestyles |
| X1060 | 1.45 | 9.08×10^-06^ | 1.47 | 2.67×10^-04^ | 1.30 | 1.000 | 1.54 | 1.62×10^-05^ | 1.60 | 0.017 | Time spent outdoors in winter | Lifestyles |
| X1070 | 1.00 | 1.000 | 1.03 | 1.000 | 0.91 | 1.000 | 0.98 | 1.000 | 1.19 | 1.000 | Time spent watching television (TV) | Lifestyles |
| X1070 | 1.62 | 7.18×10^-11^ | 1.77 | 6.93×10^-12^ | 1.22 | 1.000 | 1.54 | 5.03×10^-06^ | 1.69 | 0.002 | Time spent watching television (TV) | Lifestyles |
| X1080 | 0.61 | 2.40×10^-09^ | 0.63 | 2.96×10^-06^ | 0.57 | 0.081 | 0.61 | 9.80×10^-07^ | 0.66 | 0.190 | Time spent using computer | Lifestyles |
| X1080 | 0.70 | 4.52×10^-04^ | 0.66 | 9.01×10^-04^ | 0.81 | 1.000 | 0.71 | 0.032 | 0.72 | 1.000 | Time spent using computer | Lifestyles |
| X1488 | 0.90 | 1.000 | 0.86 | 1.000 | 1.01 | 1.000 | 0.88 | 1.000 | 0.91 | 1.000 | Tea intake | Lifestyles |
| X1488 | 1.05 | 1.000 | 1.07 | 1.000 | 1.03 | 1.000 | 0.99 | 1.000 | 1.00 | 1.000 | Tea intake | Lifestyles |
| X1528 | 1.06 | 1.000 | 1.03 | 1.000 | 1.14 | 1.000 | 1.01 | 1.000 | 0.97 | 1.000 | Water intake | Lifestyles |
| X1528 | 1.23 | 0.427 | 1.20 | 1.000 | 1.31 | 1.000 | 1.25 | 0.930 | 1.29 | 1.000 | Water intake | Lifestyles |
| X2139 | 0.82 | 1.000 | 0.79 | 0.475 | 0.96 | 1.000 | 0.82 | 1.000 | 0.79 | 1.000 | Age first had sexual intercourse | Lifestyles |
| X2139 | 0.93 | 1.000 | 0.92 | 1.000 | 0.92 | 1.000 | 0.89 | 1.000 | 0.95 | 1.000 | Age first had sexual intercourse | Lifestyles |
| X2149 | 1.13 | 1.000 | 1.17 | 1.000 | 1.08 | 1.000 | 1.11 | 1.000 | 1.30 | 1.000 | Lifetime number of sexual partners | Lifestyles |
| X2149 | 1.24 | 0.566 | 1.25 | 1.000 | 1.29 | 1.000 | 1.24 | 1.000 | 1.27 | 1.000 | Lifetime number of sexual partners | Lifestyles |
| X22037 | 0.90 | 1.000 | 0.92 | 1.000 | 0.83 | 1.000 | 0.94 | 1.000 | 0.93 | 1.000 | MET minutes per week for walking | Lifestyles |
| X22037 | 0.90 | 1.000 | 0.87 | 1.000 | 0.99 | 1.000 | 0.96 | 1.000 | 1.08 | 1.000 | MET minutes per week for walking | Lifestyles |
| X22038 | 0.94 | 1.000 | 1.00 | 1.000 | 0.77 | 1.000 | 0.90 | 1.000 | 1.03 | 1.000 | MET minutes per week for moderate activity | Lifestyles |
| X22038 | 0.98 | 1.000 | 1.04 | 1.000 | 0.78 | 1.000 | 0.95 | 1.000 | 1.09 | 1.000 | MET minutes per week for moderate activity | Lifestyles |
| X22039 | 0.72 | 6.23×10^-04^ | 0.75 | 0.117 | 0.63 | 0.288 | 0.78 | 0.614 | 0.86 | 1.000 | MET minutes per week for vigorous activity | Lifestyles |
| X22039 | 0.72 | 2.24×10^-04^ | 0.75 | 0.089 | 0.58 | 0.042 | 0.72 | 0.019 | 0.78 | 1.000 | MET minutes per week for vigorous activity | Lifestyles |
| X1498 | 0.96 | 1.000 | 0.92 | 1.000 | 1.09 | 1.000 | 1.05 | 1.000 | 0.99 | 1.000 | Coffee intake | Lifestyles |
| X1498 | 0.94 | 1.000 | 0.94 | 1.000 | 0.94 | 1.000 | 0.97 | 1.000 | 0.89 | 1.000 | Coffee intake | Lifestyles |
| X22040 | 0.82 | 1.000 | 0.81 | 1.000 | 0.82 | 1.000 | 0.83 | 1.000 | 0.78 | 1.000 | Summed MET minutes per week for all activity | Lifestyles |
| X22040 | 0.86 | 1.000 | 0.89 | 1.000 | 0.74 | 1.000 | 0.86 | 1.000 | 0.92 | 1.000 | Summed MET minutes per week for all activity | Lifestyles |
| Vegetable | 0.75 | 0.010 | 0.68 | 4.07×10^-04^ | 1.01 | 1.000 | 0.78 | 0.492 | 0.78 | 1.000 | vegetable | Lifestyles |
| Vegetable | 0.86 | 1.000 | 0.86 | 1.000 | 0.88 | 1.000 | 0.84 | 1.000 | 0.83 | 1.000 | vegetable | Lifestyles |
| Red_meat | 1.09 | 1.000 | 0.97 | 1.000 | 1.43 | 1.000 | 1.01 | 1.000 | 0.85 | 1.000 | Red meat intake reudced | Lifestyles |
| White_bread_refined_cereals | 1.24 | 1.000 | 1.32 | 0.510 | 0.98 | 1.000 | 1.17 | 1.000 | 1.17 | 1.000 | White bread/refined cereals intake reduced | Lifestyles |
| X6160_0 | 1.17 | 1.000 | 1.17 | 1.000 | 1.20 | 1.000 | 1.20 | 1.000 | 1.17 | 1.000 | Leisure/social activities | Lifestyles |
| X6160_1 | 0.59 | 1.50×10^-11^ | 0.58 | 2.53×10^-09^ | 0.63 | 0.339 | 0.60 | 2.62×10^-07^ | 0.58 | 0.003 | Leisure/social activities | Lifestyles |
| X6160_2 | 0.97 | 1.000 | 0.92 | 1.000 | 1.17 | 1.000 | 0.95 | 1.000 | 1.12 | 1.000 | Leisure/social activities | Lifestyles |
| X6160_3 | 1.17 | 1.000 | 1.22 | 1.000 | 1.01 | 1.000 | 1.05 | 1.000 | 0.99 | 1.000 | Leisure/social activities | Lifestyles |
| X6160_4 | 1.45 | 0.019 | 1.67 | 3.07×10^-04^ | 0.94 | 1.000 | 1.54 | 0.015 | 1.42 | 1.000 | Leisure/social activities | Lifestyles |
| X6160_5 | 0.96 | 1.000 | 1.07 | 1.000 | 0.72 | 1.000 | 1.01 | 1.000 | 1.00 | 1.000 | Leisure/social activities | Lifestyles |
| X1031 | 0.23 | 2.09×10^-39^ | 0.22 | 5.12×10^-32^ | 0.23 | 6.68×10^-07^ | 0.26 | 4.32×10^-19^ | 0.24 | 2.05×10^-10^ | frequency of friend family visits | Lifestyles |
| X24006 | 1.23 | 1.000 | 1.23 | 1.000 | 1.20 | 1.000 | 1.16 | 1.000 | 1.09 | 1.000 | Particulate matter air pollution (pm2.5) | Local environment |
| X24006 | 1.78 | 6.14×10^-14^ | 1.77 | 8.15×10^-10^ | 1.75 | 0.012 | 1.73 | 5.18×10^-09^ | 1.95 | 6.86×10^-06^ | Particulate matter air pollution (pm2.5) | Local environment |
| X24008 | 1.27 | 0.146 | 1.36 | 0.046 | 1.05 | 1.000 | 1.25 | 1.000 | 1.16 | 1.000 | Particulate matter air pollution 2.5-10um | Local environment |
| X24008 | 1.22 | 1.000 | 1.26 | 1.000 | 1.10 | 1.000 | 1.16 | 1.000 | 0.93 | 1.000 | Particulate matter air pollution 2.5-10um | Local environment |
| X24020 | 1.06 | 1.000 | 1.00 | 1.000 | 1.26 | 1.000 | 1.11 | 1.000 | 1.02 | 1.000 | Average daytime sound level of noise pollution | Local environment |
| X24020 | 1.14 | 1.000 | 1.11 | 1.000 | 1.20 | 1.000 | 1.11 | 1.000 | 1.06 | 1.000 | Average daytime sound level of noise pollution | Local environment |
| X24021 | 1.07 | 1.000 | 1.00 | 1.000 | 1.28 | 1.000 | 1.11 | 1.000 | 1.03 | 1.000 | Average evening sound level of noise pollution | Local environment |
| X24021 | 1.11 | 1.000 | 1.09 | 1.000 | 1.15 | 1.000 | 1.09 | 1.000 | 1.04 | 1.000 | Average evening sound level of noise pollution | Local environment |
| X24022 | 1.07 | 1.000 | 1.00 | 1.000 | 1.27 | 1.000 | 1.11 | 1.000 | 1.02 | 1.000 | Average night-time sound level of noise pollution | Local environment |
| X24022 | 1.11 | 1.000 | 1.10 | 1.000 | 1.15 | 1.000 | 1.09 | 1.000 | 1.04 | 1.000 | Average night-time sound level of noise pollution | Local environment |
| X24500 | 0.85 | 1.000 | 0.85 | 1.000 | 0.86 | 1.000 | 0.87 | 1.000 | 0.91 | 1.000 | Greenspace percentage, buffer 1000m | Local environment |
| X24500 | 0.56 | 4.94×10^-13^ | 0.57 | 6.04×10^-09^ | 0.56 | 0.016 | 0.56 | 4.80×10^-09^ | 0.56 | 0.002 | Greenspace percentage, buffer 1000m | Local environment |
| X24501 | 1.03 | 1.000 | 1.06 | 1.000 | 0.92 | 1.000 | 1.12 | 1.000 | 1.09 | 1.000 | Domestic garden percentage, buffer 1000m | Local environment |
| X24501 | 0.97 | 1.000 | 0.93 | 1.000 | 1.06 | 1.000 | 1.05 | 1.000 | 1.08 | 1.000 | Domestic garden percentage, buffer 1000m | Local environment |
| X24502 | 0.88 | 1.000 | 0.93 | 1.000 | 0.77 | 1.000 | 0.87 | 1.000 | 0.80 | 1.000 | Water percentage, buffer 1000m | Local environment |
| X24502 | 1.06 | 1.000 | 1.10 | 1.000 | 0.96 | 1.000 | 1.01 | 1.000 | 1.01 | 1.000 | Water percentage, buffer 1000m | Local environment |
| X24503 | 1.02 | 1.000 | 1.06 | 1.000 | 0.93 | 1.000 | 1.00 | 1.000 | 1.07 | 1.000 | Greenspace percentage, buffer 300m | Local environment |
| X24503 | 0.69 | 7.59×10^-05^ | 0.71 | 0.010 | 0.67 | 1.000 | 0.68 | 0.002 | 0.63 | 0.099 | Greenspace percentage, buffer 300m | Local environment |
| X24504 | 0.92 | 1.000 | 0.98 | 1.000 | 0.76 | 1.000 | 0.94 | 1.000 | 1.01 | 1.000 | Domestic garden percentage, buffer 300m | Local environment |
| X24504 | 0.82 | 1.000 | 0.79 | 1.000 | 0.87 | 1.000 | 0.87 | 1.000 | 0.90 | 1.000 | Domestic garden percentage, buffer 300m | Local environment |
| X24505 | 0.82 | 1.000 | 0.86 | 1.000 | 0.73 | 1.000 | 0.80 | 1.000 | 0.83 | 1.000 | Water percentage, buffer 300m | Local environment |
| X24505 | 0.96 | 1.000 | 1.03 | 1.000 | 0.81 | 1.000 | 0.97 | 1.000 | 0.96 | 1.000 | Water percentage, buffer 300m | Local environment |
| X24506 | 0.80 | 0.162 | 0.81 | 1.000 | 0.80 | 1.000 | 0.86 | 1.000 | 0.93 | 1.000 | Natural environment percentage, buffer 1000m | Local environment |
| X24506 | 0.53 | 2.23×10^-16^ | 0.54 | 5.22×10^-11^ | 0.51 | 7.09×10^-04^ | 0.53 | 9.26×10^-11^ | 0.51 | 9.12×10^-05^ | Natural environment percentage, buffer 1000m | Local environment |
| X24507 | 0.94 | 1.000 | 0.96 | 1.000 | 0.89 | 1.000 | 0.94 | 1.000 | 0.97 | 1.000 | Natural environment percentage, buffer 300m | Local environment |
| X24507 | 0.68 | 2.07×10^-05^ | 0.70 | 0.005 | 0.65 | 0.613 | 0.69 | 0.002 | 0.66 | 0.285 | Natural environment percentage, buffer 300m | Local environment |
| NOx | 1.44 | 4.56×10^-04^ | 1.44 | 0.013 | 1.42 | 1.000 | 1.43 | 0.017 | 1.47 | 1.000 | Nitrogen oxides | Local environment |
| NOx | 1.96 | 2.20×10^-18^ | 2.02 | 1.73×10^-14^ | 1.73 | 0.031 | 1.93 | 2.62×10^-12^ | 2.00 | 9.07×10^-06^ | Nitrogen oxides | Local environment |
| X24023 | 1.06 | 1.000 | 1.00 | 1.000 | 1.27 | 1.000 | 1.10 | 1.000 | 1.02 | 1.000 | Average 16-hour sound level of noise pollution | Local environment |
| X24023 | 1.11 | 1.000 | 1.09 | 1.000 | 1.14 | 1.000 | 1.08 | 1.000 | 1.04 | 1.000 | Average 16-hour sound level of noise pollution | Local environment |
| X24024 | 1.06 | 1.000 | 1.00 | 1.000 | 1.27 | 1.000 | 1.10 | 1.000 | 1.02 | 1.000 | Average 24-hour sound level of noise pollution | Local environment |
| X24024 | 1.11 | 1.000 | 1.09 | 1.000 | 1.14 | 1.000 | 1.08 | 1.000 | 1.04 | 1.000 | Average 24-hour sound level of noise pollution | Local environment |
| PM10 | 1.38 | 0.005 | 1.35 | 0.161 | 1.42 | 1.000 | 1.54 | 2.99×10^-04^ | 1.71 | 0.012 | Particulate matter air pollution (pm10) | Local environment |
| PM10 | 1.77 | 2.77×10^-13^ | 1.75 | 3.32×10^-09^ | 1.77 | 0.016 | 1.83 | 4.51×10^-10^ | 1.85 | 4.57×10^-04^ | Particulate matter air pollution (pm10) | Local environment |
| Arthritis | 1.29 | 0.345 | 1.47 | 0.015 | 1.00 | 1.000 | 1.30 | 1.000 | 1.33 | 1.000 | Arthritis | Medical history |
| CHD | 1.59 | 0.003 | 1.82 | 0.002 | 1.28 | 1.000 | 1.45 | 1.000 | 1.23 | 1.000 | CHD | Medical history |
| COPD | 2.26 | 2.57×10^-06^ | 2.47 | 8.02×10^-05^ | 1.94 | 1.000 | 1.96 | 0.051 | 1.74 | 1.000 | COPD | Medical history |
| Diabetes | 1.90 | 2.77×10^-08^ | 2.15 | 2.29×10^-08^ | 1.46 | 1.000 | 1.57 | 0.098 | 1.48 | 1.000 | Diabetes | Medical history |
| Hear_loss | 0.88 | 1.000 | 0.88 | 1.000 | 0.89 | 1.000 | 0.84 | 1.000 | 0.89 | 1.000 | Hearing_loss | Medical history |
| Hepertension | 0.98 | 1.000 | 0.97 | 1.000 | 1.04 | 1.000 | 0.97 | 1.000 | 0.85 | 1.000 | Hypertension | Medical history |
| X6146 | 6.08 | <2×10^-16^ | 7.65 | <2×10^-16^ | 3.16 | 1.10×10^-14^ | 5.38 | 7.44×10^-94^ | 4.07 | 1.32×10^-24^ | Disability | Medical history |
| X6149_0 | 0.60 | 1.37×10^-17^ | 0.59 | 2.60×10^-13^ | 0.63 | 0.010 | 0.61 | 6.71×10^-11^ | 0.61 | 1.42×10^-04^ | Mouth/teeth dental problems | Medical history |
| X6149_1 | 1.11 | 1.000 | 1.05 | 1.000 | 1.29 | 1.000 | 1.20 | 1.000 | 1.13 | 1.000 | Mouth/teeth dental problems | Medical history |
| X6149_2 | 2.34 | 7.30×10^-12^ | 2.39 | 1.78×10^-09^ | 2.21 | 0.250 | 2.21 | 1.39×10^-06^ | 1.99 | 0.305 | Mouth/teeth dental problems | Medical history |
| X6149_3 | 1.24 | 1.000 | 1.27 | 1.000 | 1.18 | 1.000 | 1.19 | 1.000 | 1.12 | 1.000 | Mouth/teeth dental problems | Medical history |
| X6149_4 | 1.78 | 1.37×10^-05^ | 1.91 | 2.33×10^-05^ | 1.49 | 1.000 | 1.68 | 0.015 | 1.52 | 1.000 | Mouth/teeth dental problems | Medical history |
| X6149_5 | 2.10 | 1.24×10^-11^ | 2.34 | 1.65×10^-13^ | 1.08 | 1.000 | 2.17 | 7.25×10^-09^ | 2.17 | 0.003 | Mouth/teeth dental problems | Medical history |
| X6149_6 | 1.70 | 4.91×10^-12^ | 1.74 | 3.98×10^-08^ | 1.56 | 0.026 | 1.63 | 3.35×10^-07^ | 1.65 | 0.005 | Mouth/teeth dental problems | Medical history |
| X130176 | 0.31 | 3.01×10^-05^ | 0.34 | 5.07×10^-04^ | 0.13 | 1.000 | 0.33 | 0.004 | 0.23 | 0.328 | varicella [chickenpox] | Medical history |
| X130178 | 0.49 | 1.000 | 0.72 | 1.000 | - | - | 0.22 | 0.771 | 0.24 | 1.000 | zoster [herpes zoster] | Medical history |
| X130188 | 0.79 | 1.000 | 0.74 | 1.000 | 0.97 | 1.000 | 0.69 | 1.000 | 0.61 | 1.000 | viral warts | Medical history |
| X130226 | 1.01 | 1.000 | 1.10 | 1.000 | 0.80 | 1.000 | 0.86 | 1.000 | 0.78 | 1.000 | dermatophytosis | Medical history |
| X130622 | 1.33 | 1.000 | 1.49 | 1.000 | 0.82 | 1.000 | 1.34 | 1.000 | 1.39 | 1.000 | iron deficiency anaemia | Medical history |
| X130648 | 1.81 | 0.065 | 1.83 | 0.375 | 1.74 | 1.000 | 1.28 | 1.000 | 0.68 | 1.000 | other anaemias | Medical history |
| X130696 | 1.49 | 0.067 | 1.57 | 0.121 | 1.32 | 1.000 | 1.37 | 1.000 | 1.24 | 1.000 | other hypothyroidism | Medical history |
| X130792 | 1.25 | 1.000 | 1.51 | 1.000 | 0.62 | 1.000 | 1.18 | 1.000 | 1.31 | 1.000 | obesity | Medical history |
| X130814 | 1.34 | 0.030 | 1.56 | 2.66×10^-04^ | 0.99 | 1.000 | 1.36 | 0.132 | 1.41 | 1.000 | disorders of lipoprotein metabolism and other lipidaemias | Medical history |
| X131052 | 0.91 | 1.000 | 0.93 | 1.000 | 0.90 | 1.000 | 0.92 | 1.000 | 0.80 | 1.000 | migraine | Medical history |
| X131060 | 1.64 | 0.629 | 1.89 | 0.098 | 1.04 | 1.000 | 1.65 | 1.000 | 1.65 | 1.000 | sleep disorders) | Medical history |
| X131074 | 0.92 | 1.000 | 1.06 | 1.000 | 0.62 | 1.000 | 1.02 | 1.000 | 1.06 | 1.000 | mononeuropathies of upper limb | Medical history |
| X131128 | 0.84 | 1.000 | 0.94 | 1.000 | 0.58 | 1.000 | 0.75 | 1.000 | 0.45 | 1.000 | hordeolum and chalazion | Medical history |
| X131142 | 1.11 | 1.000 | 1.14 | 1.000 | 1.00 | 1.000 | 0.91 | 1.000 | 0.93 | 1.000 | conjunctivitis | Medical history |
| X131166 | 1.50 | 1.000 | 1.65 | 1.000 | 1.25 | 1.000 | 1.23 | 1.000 | 0.96 | 1.000 | other cataract | Medical history |
| X131222 | 0.92 | 1.000 | 0.84 | 1.000 | 1.16 | 1.000 | 0.90 | 1.000 | 0.63 | 1.000 | otitis externa | Medical history |
| X131224 | 0.78 | 1.000 | 0.78 | 1.000 | 0.76 | 1.000 | 0.78 | 1.000 | 0.71 | 1.000 | other disorders of external ear | Medical history |
| X131262 | 1.01 | 1.000 | 0.98 | 1.000 | 1.12 | 1.000 | 1.03 | 1.000 | 0.59 | 1.000 | otalgia and effusion of ear | Medical history |
| X131264 | 1.11 | 1.000 | 1.41 | 1.000 | 0.47 | 1.000 | 0.92 | 1.000 | 0.63 | 1.000 | other disorders of ear, not elsewhere classified | Medical history |
| X131350 | 1.27 | 1.000 | 1.02 | 1.000 | 1.45 | 1.000 | 1.29 | 1.000 | 1.43 | 1.000 | atrial fibrillation and flutter | Medical history |
| X131396 | 1.68 | 0.101 | 1.60 | 1.000 | 1.79 | 1.000 | 1.46 | 1.000 | 1.60 | 1.000 | phlebitis and thrombophlebitis | Medical history |
| X131402 | 0.91 | 1.000 | 0.68 | 1.000 | 1.40 | 1.000 | 0.82 | 1.000 | 0.85 | 1.000 | varicose veins of lower extremities | Medical history |
| X131404 | 1.36 | 1.000 | 1.68 | 0.251 | 0.63 | 1.000 | 1.16 | 1.000 | 1.24 | 1.000 | haemorrhoids | Medical history |
| X131426 | 0.86 | 1.000 | 0.86 | 1.000 | 0.88 | 1.000 | 0.92 | 1.000 | 0.78 | 1.000 | acute sinusitis | Medical history |
| X131428 | 0.79 | 1.000 | 0.86 | 1.000 | 0.58 | 1.000 | 0.83 | 1.000 | 0.75 | 1.000 | acute pharyngitis | Medical history |
| X131430 | 0.57 | 1.000 | 0.63 | 1.000 | 0.26 | 1.000 | 0.62 | 1.000 | 0.73 | 1.000 | acute tonsillitis | Medical history |
| X131436 | 1.10 | 1.000 | 1.15 | 1.000 | 0.97 | 1.000 | 1.04 | 1.000 | 0.98 | 1.000 | acute upper respiratory infections of multiple and unspecified sites | Medical history |
| X131442 | 0.68 | 1.000 | 0.63 | 1.000 | 0.85 | 1.000 | 0.74 | 1.000 | 0.75 | 1.000 | influenza, virus not identified | Medical history |
| X131456 | 1.41 | 1.000 | 1.53 | 1.000 | 1.16 | 1.000 | 1.30 | 1.000 | 1.76 | 1.000 | pneumonia, organism unspecified | Medical history |
| X131462 | 1.29 | 1.000 | 1.39 | 1.000 | 1.06 | 1.000 | 1.25 | 1.000 | 1.37 | 1.000 | unspecified acute lower respiratory infection | Medical history |
| X131464 | 0.90 | 1.000 | 0.88 | 1.000 | 0.98 | 1.000 | 0.89 | 1.000 | 0.94 | 1.000 | vasomotor and allergic rhinitis | Medical history |
| X131468 | 0.75 | 1.000 | 0.96 | 1.000 | 0.17 | 1.000 | 0.80 | 1.000 | 1.27 | 1.000 | chronic sinusitis | Medical history |
| X131472 | 1.15 | 1.000 | 1.24 | 1.000 | 0.91 | 1.000 | 1.25 | 1.000 | 1.93 | 1.000 | other disorders of nose and nasal sinuses | Medical history |
| X131494 | 1.51 | 1.55×10^-05^ | 1.57 | 3.12×10^-05^ | 1.28 | 1.000 | 1.45 | 0.013 | 1.62 | 0.051 | asthma | Medical history |
| X131584 | 1.32 | 1.000 | 1.36 | 1.000 | 1.24 | 1.000 | 1.36 | 1.000 | 1.61 | 0.508 | gastro -oesophageal reflux Medical history | Medical history |
| X131598 | 1.81 | 3.05×10^-06^ | 1.99 | 4.69×10^-06^ | 1.44 | 1.000 | 1.61 | 0.061 | 1.35 | 1.000 | gastritis and duodenitis | Medical history |
| X131600 | 1.61 | 0.722 | 1.65 | 1.000 | 1.51 | 1.000 | 1.74 | 0.538 | 1.48 | 1.000 | dyspepsia | Medical history |
| X131612 | 0.85 | 1.000 | 0.83 | 1.000 | 0.90 | 1.000 | 0.93 | 1.000 | 1.23 | 1.000 | inguinal hernia | Medical history |
| X131620 | 1.59 | 0.007 | 1.80 | 0.002 | 1.23 | 1.000 | 1.55 | 0.200 | 1.57 | 1.000 | diaphragmatic hernia | Medical history |
| X131630 | 2.28 | 3.91×10^-10^ | 2.55 | 3.28×10^-10^ | 1.63 | 1.000 | 2.07 | 1.09×10^-04^ | 1.65 | 1.000 | other non -infective gastro -enteritis and colitis | Medical history |
| X131636 | 1.33 | 1.000 | 1.34 | 1.000 | 1.27 | 1.000 | 1.15 | 1.000 | 0.98 | 1.000 | diverticular Medical history of intestine | Medical history |
| X131638 | 1.39 | 0.934 | 1.36 | 1.000 | 1.50 | 1.000 | 1.30 | 1.000 | 1.21 | 1.000 | irritable bowel syndrome | Medical history |
| X131640 | 2.41 | 4.53×10^-07^ | 2.42 | 1.27×10^-04^ | 2.34 | 0.370 | 2.23 | 0.002 | 2.23 | 0.666 | other functional intestinal disorders | Medical history |
| X131646 | 1.39 | 1.000 | 1.61 | 0.103 | 0.92 | 1.000 | 1.33 | 1.000 | 1.27 | 1.000 | other Medical historys of anus and rectum | Medical history |
| X131650 | 0.79 | 1.000 | 0.85 | 1.000 | 0.64 | 1.000 | 0.64 | 1.000 | 0.76 | 1.000 | haemorrhoids and perianal venous thrombosis | Medical history |
| X131674 | 0.94 | 1.000 | 1.04 | 1.000 | 0.72 | 1.000 | 0.84 | 1.000 | 0.55 | 1.000 | cholelithiasis | Medical history |
| X131702 | 1.51 | 1.000 | 1.44 | 1.000 | 1.65 | 1.000 | 1.45 | 1.000 | 1.80 | 1.000 | cellulitis | Medical history |
| X131708 | 1.24 | 1.000 | 1.14 | 1.000 | 1.51 | 1.000 | 1.21 | 1.000 | 2.08 | 1.000 | other local infections of skin and subcutaneous tissue | Medical history |
| X131720 | 0.78 | 1.000 | 0.83 | 1.000 | 0.62 | 1.000 | 0.76 | 1.000 | 0.96 | 1.000 | atopic dermatitis | Medical history |
| X131740 | 0.88 | 1.000 | 0.91 | 1.000 | 0.78 | 1.000 | 0.85 | 1.000 | 0.95 | 1.000 | other dermatitis | Medical history |
| X131742 | 0.87 | 1.000 | 1.03 | 1.000 | 0.41 | 1.000 | 0.67 | 1.000 | 0.97 | 1.000 | psoriasis | Medical history |
| X131768 | 0.77 | 1.000 | 0.84 | 1.000 | 0.67 | 1.000 | 0.83 | 1.000 | 0.88 | 1.000 | skin changes due to chronic exposure to nonionising radiation | Medical history |
| X131794 | 0.96 | 1.000 | 1.03 | 1.000 | 0.78 | 1.000 | 1.16 | 1.000 | 1.43 | 1.000 | follicular cysts of skin and subcutaneous tissue | Medical history |
| X131806 | 0.70 | 1.000 | 0.85 | 1.000 | 0.48 | 1.000 | 0.54 | 1.000 | 0.62 | 1.000 | seborrhoeic keratosis | Medical history |
| X131836 | 0.78 | 1.000 | 0.89 | 1.000 | 0.56 | 1.000 | 0.80 | 1.000 | 0.74 | 1.000 | other disorders of skin and subcutaneous tissue, not elsewhere classified | Medical history |
| X131858 | 0.92 | 1.000 | 1.02 | 1.000 | 0.77 | 1.000 | 0.84 | 1.000 | 0.77 | 1.000 | gout | Medical history |
| X131864 | 1.18 | 1.000 | 1.15 | 1.000 | 1.22 | 1.000 | 0.92 | 1.000 | 1.04 | 1.000 | other arthritis | Medical history |
| X131872 | 1.52 | 0.686 | 1.39 | 1.000 | 1.67 | 1.000 | 1.38 | 1.000 | 1.63 | 1.000 | gonarthrosis [arthrosis of knee] | Medical history |
| X131876 | 1.20 | 1.000 | 1.47 | 0.043 | 0.79 | 1.000 | 1.23 | 1.000 | 1.20 | 1.000 | other arthrosis | Medical history |
| X131878 | 1.20 | 1.000 | 1.09 | 1.000 | 1.42 | 1.000 | 1.19 | 1.000 | 1.00 | 1.000 | acquired deformities of fingers and toes | Medical history |
| X131884 | 0.86 | 1.000 | 0.83 | 1.000 | 0.96 | 1.000 | 0.60 | 1.000 | 0.44 | 1.000 | internal derangement of knee | Medical history |
| X131888 | 0.98 | 1.000 | 1.00 | 1.000 | 0.93 | 1.000 | 0.88 | 1.000 | 0.91 | 1.000 | other joint disorders, not elsewhere classified | Medical history |
| X131916 | 1.43 | 1.000 | 1.80 | 0.028 | 0.88 | 1.000 | 1.51 | 1.000 | 1.38 | 1.000 | spondylosis | Medical history |
| X131924 | 0.84 | 1.000 | 0.75 | 1.000 | 1.09 | 1.000 | 0.93 | 1.000 | 0.96 | 1.000 | other intervertebral disk disorders | Medical history |
| X131928 | 1.19 | 1.000 | 1.23 | 1.000 | 1.08 | 1.000 | 1.11 | 1.000 | 0.97 | 1.000 | dorsalgia | Medical history |
| X131938 | 0.93 | 1.000 | 1.07 | 1.000 | 0.64 | 1.000 | 0.94 | 1.000 | 1.33 | 1.000 | synovitis and tenosynovitis | Medical history |
| X131946 | 0.74 | 1.000 | 0.87 | 1.000 | 0.46 | 1.000 | 0.83 | 1.000 | 0.85 | 1.000 | soft tissue disorders related to use, overuse and pressure | Medical history |
| X131950 | 0.77 | 1.000 | 0.85 | 1.000 | 0.63 | 1.000 | 0.60 | 1.000 | 0.80 | 1.000 | fibroblastic disorders | Medical history |
| X131954 | 0.82 | 1.000 | 0.93 | 1.000 | 0.61 | 1.000 | 0.83 | 1.000 | 0.68 | 1.000 | shoulder lesions | Medical history |
| X131958 | 0.77 | 1.000 | 0.85 | 1.000 | 0.60 | 1.000 | 0.80 | 1.000 | 0.91 | 1.000 | other enthesopathies | Medical history |
| X131960 | 1.14 | 1.000 | 1.27 | 1.000 | 0.85 | 1.000 | 1.06 | 1.000 | 1.11 | 1.000 | other soft tissue disorders, not elsewhere classified | Medical history |
| X131964 | 1.73 | 0.156 | 1.97 | 0.227 | 1.36 | 1.000 | 1.43 | 1.000 | 1.44 | 1.000 | osteoporosis without pathological fracture | Medical history |
| X132054 | 1.29 | 1.000 | 1.28 | 1.000 | 1.29 | 1.000 | 1.13 | 1.000 | 1.44 | 1.000 | cystitis | Medical history |
| X132070 | 1.38 | 0.877 | 1.40 | 1.000 | 1.31 | 1.000 | 1.12 | 1.000 | 0.95 | 1.000 | other disorders of urinary system | Medical history |
| X132072 | 0.97 | 1.000 | 1.36 | 1.000 | 0.61 | 1.000 | 0.90 | 1.000 | 0.89 | 1.000 | hyperplasia of prostate | Medical history |
| X132096 | 0.71 | 1.000 | 0.90 | 1.000 | 0.25 | 1.000 | 0.71 | 1.000 | 0.71 | 1.000 | benign mammary dysplasia | Medical history |
| X132102 | 0.93 | 1.000 | 1.00 | 1.000 | 0.74 | 1.000 | 0.85 | 1.000 | 0.73 | 1.000 | unspecified lump in breast | Medical history |
| X132104 | 0.64 | 1.000 | 0.71 | 1.000 | 0.40 | 1.000 | 0.64 | 1.000 | 0.90 | 1.000 | other disorders of breast | Medical history |
| X132124 | 1.27 | 1.000 | 1.27 | 1.000 | 1.22 | 1.000 | 1.11 | 1.000 | 1.37 | 1.000 | female genital prolapse | Medical history |
| X132128 | 1.40 | 1.000 | 1.45 | 1.000 | 1.26 | 1.000 | 1.22 | 1.000 | 1.01 | 1.000 | noninflammatory disorders of ovary, fallopian tube and broad ligament | Medical history |
| X132130 | 0.82 | 1.000 | 0.93 | 1.000 | 0.60 | 1.000 | 0.79 | 1.000 | 1.04 | 1.000 | polyp of female genital tract | Medical history |
| X132140 | 0.97 | 1.000 | 0.90 | 1.000 | 1.22 | 1.000 | 0.95 | 1.000 | 1.25 | 1.000 | other noninflammatory disorders of vagina | Medical history |
| X132146 | 0.83 | 1.000 | 0.87 | 1.000 | 0.67 | 1.000 | 0.66 | 1.000 | 0.66 | 1.000 | excessive, frequent and irregular menstruation | Medical history |
| X132148 | 0.77 | 1.000 | 0.80 | 1.000 | 0.70 | 1.000 | 0.73 | 1.000 | 0.89 | 1.000 | other abnormal uterine and vaginal bleeding | Medical history |
| X132150 | 1.20 | 1.000 | 1.24 | 1.000 | 1.03 | 1.000 | 1.05 | 1.000 | 0.86 | 1.000 | pain and other conditions associated with female genital organs and menstrual cycle | Medical history |
| X132152 | 1.08 | 1.000 | 1.17 | 1.000 | 0.90 | 1.000 | 1.02 | 1.000 | 0.99 | 1.000 | menopausal and other perimenopausal disorders | Medical history |
| X132276 | 0.86 | 1.000 | 0.83 | 1.000 | 0.97 | 1.000 | 0.74 | 1.000 | 0.57 | 1.000 | single spontaneous delivery | Medical history |
| Stroke | 2.33 | 6.47×10^-06^ | 2.62 | 6.42×10^-05^ | 1.88 | 1.000 | 2.32 | 6.48×10^-04^ | 1.87 | 1.000 | stroke | Medical history |
| TBI | 1.05 | 1.000 | 1.05 | 1.000 | 0.91 | 1.000 | 0.98 | 1.000 | 0.99 | 1.000 | Traumatic brain injury | Medical history |
| Vision_impairment | 0.89 | 1.000 | 0.93 | 1.000 | 0.74 | 1.000 | 0.83 | 1.000 | 0.84 | 1.000 | Vision_impairment | Medical history |
| Dementia | 7.52 | 0.147 | 13.43 | 0.002 | - | - | 7.51 | 1.000 | - | - | dementia | Mental health |
| Intellectual disability | 35.15 | 2.96×10^-07^ | 34.75 | 3.09×10^-07^ | - | - | 35.91 | 1.84×10^-04^ | - | - | Intellectual disability | Mental health |
| Others personality disorders | 3.76 | 1.000 | 4.10 | 1.000 | - | - | 2.71 | 1.000 | - | - | others disorders of adult personality and behaviour | Mental health |
| Others behavioural syndromes | 22.29 | 0.671 | - | - | 91.93 | 0.014 | 32.35 | 0.209 | - | - | others behavioural syndromes associated with physiological disturbances and physical factors | Mental health |
| Others substance abuse | 5.98 | 1.000 | 6.01 | 1.000 | - | - | 9.05 | 1 | - | - | others mental and behavioural disorders due to psychoactive substance use | Mental health |
| Others Mood disorders | 5.09 | 0.001 | 5.74 | 2.59×10^-04^ | - | - | 5.41 | 0.011 | 4.17 | 1.000 | others mood [affective] disorders | Mental health |
| Other organic mental disorders | 20.63 | 1.40×10^-72^ | 19.09 | 1.54×10^-50^ | 27.00 | 2.84×10^-22^ | 20.15 | 9.55×10^-49^ | 10.60 | 2.63×10^-06^ | other organic, including symptomatic, mental disorders | Mental health |
| Other mental disorders | 12.02 | 1.58×10^-16^ | 11.59 | 1.55×10^-13^ | 15.12 | 0.054 | 11.95 | 3.63×10^-11^ | 22.08 | 1.87×10^-13^ | other mental and behavioural disorders | Mental health |
| Psychological development disorders | 23.73 | 6.93×10^-27^ | 26.47 | 8.30×10^-29^ | - | - | 18.87 | 3.82×10^-12^ | 13.24 | 0.082 | Disorders of psychological development | Mental health |
| X130854 | 5.80 | 1.11×10^-33^ | 6.52 | 2.28×10^-34^ | 2.97 | 1.000 | 4.20 | 6.88×10^-11^ | 4.51 | 9.00×10^-05^ | Mental and behavioural disorders due to use of alcohol | Mental health |
| X130856 | 15.93 | 2.78×10^-07^ | 15.77 | 3.07×10^-07^ | - | - | 9.89 | 0.355 | 25.84 | 0.002 | Mental and behavioural disorders due to use of opioids | Mental health |
| X130858 | 20.05 | 6.80×10^-07^ | 21.00 | 3.33×10^-07^ | - | - | 15.51 | 0.029 | 19.72 | 0.795 | Mental and behavioural disorders due to use of cannabinoids | Mental health |
| X130860 | 2.13 | 1.000 | - | - | 6.77 | 1.000 | 2.94 | 1.000 | - | - | Mental and behavioural disorders due to use of sedatives or hypnotics | Mental health |
| X130868 | 1.69 | 5.87×10^-04^ | 1.74 | 0.002 | 1.53 | 1.000 | 1.75 | 0.004 | 1.78 | 0.782 | Mental and behavioural disorders due to use of tobacco | Mental health |
| X130872 | 15.62 | 2.36×10^-10^ | 18.38 | 1.15×10^-11^ | - | - | 6.47 | 1.000 | 15.53 | 0.033 | Mental and behavioural disorders due to multiple drug use and use of other psychoactive substances | Mental health |
| X130890 | 33.14 | 2.50×10^-50^ | 35.97 | 4.20×10^-45^ | 24.27 | 2.34×10^-05^ | 25.88 | 5.98×10^-24^ | 21.90 | 3.37×10^-07^ | Manic episode | Mental health |
| X130892 | 32.91 | <2×10^-16^ | 33.70 | <2×10^-16^ | 31.48 | 1.64×10^-48^ | 32.42 | <2×10^-16^ | 23.77 | 4.32×10^-50^ | Bipolar affective disorder | Mental health |
| X130894 | 4.95 | <2×10^-16^ | 5.27 | <2×10^-16^ | 4.22 | 5.15×10^-23^ | 4.38 | 2.67×10^-84^ | 3.98 | 2.67×10^-31^ | Depressive episode | Mental health |
| X130896 | 8.85 | 3.19×10^-54^ | 9.75 | 3.20×10^-51^ | 5.71 | 0.002 | 7.49 | 5.98×10^-28^ | 4.91 | 1.90×10^-04^ | Recurrent depressive disorder | Mental health |
| X130898 | 5.32 | 1.91×10^-04^ | 5.89 | 1.94×10^-04^ | 3.20 | 1.000 | 4.15 | 0.458 | 3.76 | 1.000 | Persistent mood [affective] disorders | Mental health |
| X130904 | 2.33 | 1.000 | 2.76 | 1.000 | 1.05 | 1.000 | 2.50 | 1.000 | 0.78 | 1.000 | Phobic anxiety disorders | Mental health |
| X130906 | 3.55 | 1.55×10^-45^ | 3.72 | 4.90×10^-39^ | 3.11 | 3.12×10^-06^ | 3.08 | 7.85×10^-23^ | 2.02 | 0.070 | Other anxiety disorders | Mental health |
| X130908 | 11.05 | 2.71×10^-20^ | 11.53 | 1.79×10^-18^ | 8.65 | 0.684 | 11.97 | 2.24×10^-16^ | 10.50 | 5.45×10^-05^ | Obsessiv×10-compulsive disorder | Mental health |
| X130910 | 1.78 | 0.037 | 1.86 | 0.042 | 1.55 | 1.000 | 1.67 | 1.000 | 0.83 | 1.000 | Reaction to severe stress, and adjustment disorders | Mental health |
| X130912 | 18.48 | 4.07×10^-12^ | 23.87 | 1.28×10^-14^ | - | - | 22.19 | 1.32×10^-11^ | 24.91 | 9.96×10^-06^ | Dissociative [conversion] disorders | Mental health |
| X130914 | 2.47 | 0.558 | 2.53 | 1.000 | 2.32 | 1.000 | 1.98 | 1.000 | 1.85 | 1.000 | Somatoform disorders | Mental health |
| X130916 | 14.58 | 3.06×10^-52^ | 15.85 | 1.04×10^-42^ | 12.05 | 1.02×10^-09^ | 13.19 | 1.03×10^-31^ | 15.22 | 4.11×10^-18^ | Other neurotic disorders | Mental health |
| X130918 | 5.19 | 7.71×10^-05^ | 5.52 | 2.84×10^-05^ | - | - | 4.41 | 0.095 | - | - | Eating disorders | Mental health |
| X130920 | 2.03 | 1.000 | 1.43 | 1.000 | 3.58 | 1.000 | - | - | - | - | Nonorganic sleep disorders | Mental health |
| X130922 | 1.97 | 1.000 | 2.19 | 1.000 | 1.28 | 1.000 | 1.18 | 1.000 | 0.89 | 1.000 | Sexual dysfunction, not caused by organic disorder or disease | Mental health |
| X130924 | 4.26 | 2.49×10^-05^ | 4.26 | 2.14×10^-04^ | 4.20 | 1.000 | 4.67 | 1.37×10^-04^ | 4.78 | 0.158 | Mental and behavioural disorders associated with the puerperium, not elsewhere classified | Mental health |
| X130932 | 20.10 | 4.22×10^-32^ | 21.84 | 4.39×10^-32^ | 8.69 | 1.000 | 20.72 | 6.79×10^-23^ | 16.35 | 7.81×10^-06^ | Specific personality disorders | Mental health |
| X130944 | 1.08 | 1.000 | - | - | 2.77 | 1.000 | - | - | - | - | Psychological and behavioural disorders associated with sexual development and orientation | Mental health |
| X1920 | 1.86 | 4.65×10^-25^ | 2.00 | 3.61×10^-22^ | 1.52 | 0.044 | 1.73 | 7.27×10^-14^ | 1.54 | 0.004 | Mood swings | Mental health |
| X1930 | 1.53 | 1.70×10^-11^ | 1.62 | 1.32×10^-10^ | 1.32 | 1.000 | 1.47 | 2.89×10^-06^ | 1.30 | 1.000 | Miserableness | Mental health |
| X1940 | 1.21 | 0.448 | 1.27 | 0.115 | 1.01 | 1.000 | 1.20 | 1.000 | 1.11 | 1.000 | Irritability | Mental health |
| X1950 | 1.39 | 7.48×10^-06^ | 1.41 | 1.48×10^-04^ | 1.34 | 1.000 | 1.41 | 2.32×10^-04^ | 1.30 | 1.000 | Sensitivity / hurt feelings | Mental health |
| X1960 | 1.61 | 4.53×10^-15^ | 1.67 | 1.53×10^-12^ | 1.48 | 0.138 | 1.53 | 3.17×10^-08^ | 1.33 | 1.000 | Fed-up feelings | Mental health |
| X1970 | 2.46 | 1.57×10^-53^ | 2.72 | 3.25×10^-50^ | 1.79 | 2.41×10^-04^ | 2.32 | 3.28×10^-33^ | 2.16 | 6.12×10^-12^ | Nervous feelings | Mental health |
| X1980 | 1.79 | 1.01×10^-18^ | 1.99 | 1.49×10^-18^ | 1.36 | 1.000 | 1.80 | 1.67×10^-13^ | 1.63 | 0.001 | Worrier / anxious feelings | Mental health |
| X1990 | 2.40 | 8.67×10^-47^ | 2.55 | 5.28×10^-41^ | 1.93 | 3.26×10^-05^ | 2.34 | 1.24×10^-31^ | 2.36 | 3.49×10^-14^ | Tense / 'highly strung | Mental health |
| X2000 | 1.32 | 2.40×10^-04^ | 1.41 | 6.70×10^-05^ | 1.13 | 1.000 | 1.31 | 0.014 | 1.29 | 1.000 | Worry too long after embarrassment | Mental health |
| X2010 | 2.69 | 4.51×10^-66^ | 2.86 | 2.42×10^-56^ | 2.24 | 2.40×10^-09^ | 2.44 | 9.10×10^-38^ | 2.35 | 5.39×10^-15^ | Suffer from 'nerves' | Mental health |
| X2020 | 2.53 | 9.57×10^-53^ | 2.65 | 8.51×10^-45^ | 2.17 | 3.28×10^-07^ | 2.34 | 6.14×10^-31^ | 2.01 | 2.37×10^-08^ | Loneliness, isolation | Mental health |
| X2030 | 1.54 | 5.91×10^-11^ | 1.56 | 1.02×10^-08^ | 1.48 | 0.285 | 1.45 | 2.28×10^-05^ | 1.17 | 1.000 | Guilty feelings | Mental health |
| X2110 | 1.42 | 1.04×10^-06^ | 1.45 | 1.70×10^-05^ | 1.32 | 1.000 | 1.42 | 1.95×10^-04^ | 1.50 | 0.029 | Able to confide | Mental health |
| Depression | 2.48 | 4.97×10^-55^ | 2.57 | 3.27×10^-45^ | 2.23 | 1.29×10^-08^ | 2.30 | 1.23×10^-32^ | 2.10 | 6.58×10^-11^ | frequency of depressed mood in last 2 weeks | Mental health |
| X6145 | 1.53 | 1.56×10^-11^ | 1.60 | 2.84×10^-10^ | 1.36 | 1.000 | 1.42 | 3.40×10^-05^ | 1.38 | 0.339 | illness injury bereavement stress in last 2 years | Mental health |
| X2090 | 3.46 | 5.20×10^-95^ | 3.84 | 1.76×10^-78^ | 2.79 | 4.20×10^-17^ | 3.26 | 7.71×10^-63^ | 2.49 | 7.01×10^-17^ | seen doctor gp for nerves anxiety tension or depression | Mental health |
| X2100 | 6.64 | <2×10^-16^ | - | - | 5.60 | 6.68×10^-49^ | 5.96 | <2×10^-16^ | 4.82 | 2.00×10^-51^ | seen a psychiatrist for nerves anxiety tension or depression | Mental health |
| X2070 | 2.27 | 2.09×10^-45^ | 2.32 | 4.71×10^-36^ | 2.14 | 1.62×10^-08^ | 2.08 | 9.64×10^-26^ | 1.98 | 2.86×10^-09^ | frequency of tenseness restlessness in last 2 week | Mental health |
| X2080 | 1.69 | 2.35×10^-16^ | 1.72 | 2.46×10^-12^ | 1.61 | 0.008 | 1.66 | 7.56×10^-11^ | 1.57 | 0.003 | frequency of tiredness lethargy in last 2 weeks | Mental health |
| X2060 | 2.44 | 2.47×10^-52^ | 2.55 | 4.69×10^-44^ | 2.12 | 4.71×10^-07^ | 2.24 | 8.88×10^-30^ | 2.22 | 2.33×10^-12^ | frequency of unenthusiasm disinterest in last 2 weeks | Mental health |
| Arm_fat_percentage | 1.30 | 0.261 | 1.41 | 0.047 | 1.05 | 1.000 | 1.15 | 1.000 | 0.97 | 1.000 | Arm fat percentage | Physical measures |
| Arm_fat_percentage | 1.80 | 3.71×10^-06^ | 1.94 | 6.92×10^-06^ | 1.46 | 1.000 | 1.70 | 0.003 | 1.62 | 1.000 | Arm fat percentage | Physical measures |
| X4079 | 0.83 | 1.000 | 0.85 | 1.000 | 0.80 | 1.000 | 0.83 | 1.000 | 0.95 | 1.000 | Diastolic blood pressure | Physical measures |
| X4079 | 0.97 | 1.000 | 1.05 | 1.000 | 0.81 | 1.000 | 0.89 | 1.000 | 0.88 | 1.000 | Diastolic blood pressure | Physical measures |
| Hand_grip_strength | 0.62 | 1.30×10^-07^ | 0.58 | 1.17×10^-07^ | 0.79 | 1.000 | 0.67 | 0.006 | 0.79 | 1.000 | Hand grip strength | Physical measures |
| Hand_grip_strength | 0.35 | 3.53×10^-20^ | 0.30 | 1.74×10^-21^ | 0.61 | 1.000 | 0.42 | 2.30×10^-09^ | 0.51 | 0.100 | Hand grip strength | Physical measures |
| Leg_fat_percentage | 1.23 | 1.000 | 1.33 | 1.000 | 1.02 | 1.000 | 1.19 | 1.000 | 0.92 | 1.000 | Leg fat percentage | Physical measures |
| Leg_fat_percentage | 1.66 | 0.010 | 1.83 | 0.004 | 1.30 | 1.000 | 1.72 | 0.059 | 1.40 | 1.000 | Leg fat percentage | Physical measures |
| Lung_function | 0.61 | 3.52×10^-11^ | 0.62 | 3.31×10^-07^ | 0.58 | 0.036 | 0.60 | 4.69×10^-08^ | 0.71 | 1.000 | Lung_function | Physical measures |
| Lung_function | 0.40 | 5.05×10^-26^ | 0.39 | 1.14×10^-21^ | 0.46 | 0.004 | 0.40 | 3.63×10^-18^ | 0.42 | 4.51×10^-06^ | Lung_function | Physical measures |
| X23099 | 1.22 | 1.000 | 1.34 | 0.294 | 0.95 | 1.000 | 1.11 | 1.000 | 0.90 | 1.000 | Body fat percentage | Physical measures |
| X23099 | 1.55 | 0.002 | 1.72 | 6.55×10^-04^ | 1.16 | 1.000 | 1.53 | 0.067 | 1.26 | 1.000 | Body fat percentage | Physical measures |
| X23127 | 1.14 | 1.000 | 1.25 | 1.000 | 0.86 | 1.000 | 1.05 | 1.000 | 1.00 | 1.000 | Trunk fat percentage | Physical measures |
| X23127 | 1.43 | 0.001 | 1.52 | 0.001 | 1.20 | 1.000 | 1.41 | 0.052 | 1.31 | 1.000 | Trunk fat percentage | Physical measures |
| X23105 | 1.12 | 1.000 | 1.10 | 1.000 | 1.20 | 1.000 | 1.25 | 1.000 | 1.18 | 1.000 | Basal metabolic rate | Physical measures |
| X23105 | 1.08 | 1.000 | 1.10 | 1.000 | 1.07 | 1.000 | 1.16 | 1.000 | 1.04 | 1.000 | Basal metabolic rate | Physical measures |
| X4080 | 0.84 | 1.000 | 0.86 | 1.000 | 0.76 | 1.000 | 0.84 | 1.000 | 1.04 | 1.000 | Systolic blood pressure | Physical measures |
| X4080 | 0.89 | 1.000 | 0.99 | 1.000 | 0.66 | 0.969 | 0.90 | 1.000 | 0.95 | 1.000 | Systolic blood pressure | Physical measures |
| X6138 | 1.50 | 1.01×10^-09^ | 1.55 | 1.56×10^-08^ | 1.31 | 1.000 | 1.43 | 6.28×10^-05^ | 1.61 | 0.001 | Lower education | SES |
| X6142 | 2.07 | 2.04×10^-07^ | 2.31 | 2.33×10^-08^ | 1.38 | 1.000 | 1.96 | 7.92×10^-04^ | 1.84 | 1.000 | Unemployed | SES |
| X738 | 0.44 | 3.41×10^-29^ | 0.38 | 2.64×10^-28^ | 0.64 | 0.131 | 0.46 | 2.86×10^-18^ | 0.51 | 1.16×10^-05^ | Average total household income before tax | SES |
| X738 | 0.28 | 5.09×10^-48^ | 0.24 | 2.07×10^-48^ | 0.51 | 0.050 | 0.34 | 6.61×10^-27^ | 0.40 | 3.74×10^-08^ | Average total household income before tax | SES |
| X738 | 0.19 | 1.62×10^-48^ | 0.18 | 1.01×10^-48^ | 0.27 | 0.093 | 0.22 | 3.08×10^-29^ | 0.26 | 1.26×10^-10^ | Average total household income before tax | SES |
| X738 | 0.14 | 9.80×10^-16^ | 0.12 | 3.97×10^-16^ | 0.33 | 1.000 | 0.18 | 1.76×10^-09^ | 0.05 | 0.010 | Average total household income before tax | SES |
| X22189 | 1.28 | 0.890 | 1.21 | 1.000 | 1.43 | 1.000 | 1.16 | 1.000 | 1.23 | 1.000 | Townsend deprivation index at recruitment | SES |
| X22189 | 2.65 | 5.38×10^-39^ | 2.75 | 1.90×10^-30^ | 2.28 | 1.65×10^-06^ | 2.36 | 1.70×10^-22^ | 2.29 | 4.90×10^-09^ | Townsend deprivation index at recruitment | SES |
|  | | | | | | | | | | | | |

| **Supplementary Table 9. Associations between 232 risk factors and late onset schizophrenia in EWAS (by sex and genetic risk).** Blue cells indicate P.adjust<0.05; P.adjust, after Bonferroni correction; Genetic risk of schizophrenia was measured by polygenic risk score (PRS), which was computed using a Bayesian methodological framework, utilizing meta-analyzed data from three genome-wide association studies (GWAS) with a combined sample size of 97,456 cases and 334,331 controls from Thompson DJ (2024). | | | | | | | | | | | | | | | |
| --- | --- | --- | --- | --- | --- | --- | --- | --- | --- | --- | --- | --- | --- | --- | --- |
|  | **Whole sample** | | **Sex** | | | | **Genetic risk** | | | | | | **Exposures** | **Category** | |
|  |  |  | **Male** | | **Female** | | **Low genetic risk** | | **Medium genetic risk** | | **High genetic risk** | |  |  | |
| **FieldID** | **HR** | **P.adjust** | **HR** | **P.adjust** | **HR** | **P.adjust** | **HR** | **P.adjust** | **HR** | **P.adjust** | **HR** | **P.adjust** |  |  | |
| Alcohol_level | 1.53 | 1.49×10^-10^ | 1.68 | 0.002 | 1.40 | 1.000 | 1.40 | 1.000 | 1.68 | 0.002 | 1.51 | 1.61×10^-04^ | Improper consumption of alcohol | Lifestyles | |
| Ever_smoke | 0.94 | 1.000 | 0.95 | 1.000 | 1.05 | 1.000 | 1.05 | 1.000 | 0.95 | 1.000 | 0.89 | 1.000 | Ever_smok | Lifestyles | |
| Fish | 1.22 | 0.584 | 1.27 | 1.000 | 1.04 | 1.000 | 1.04 | 1.000 | 1.27 | 1.000 | 1.28 | 1.000 | Fish eaten above recommendation | Lifestyles | |
| Fruit | 1.04 | 1.000 | 0.95 | 1.000 | 0.98 | 1.000 | 0.98 | 1.000 | 0.95 | 1.000 | 1.12 | 1.000 | Fruit eaten above recommendation | Lifestyles | |
| Wholegrain | 1.17 | 1.000 | 1.32 | 1.000 | 1.07 | 1.000 | 1.07 | 1.000 | 1.32 | 1.000 | 1.14 | 1.000 | Wholegrain eaten above ecommendation | Lifestyles | |
| X1120 | 0.94 | 1.000 | 1.12 | 1.000 | 0.85 | 1.000 | 0.85 | 1.000 | 1.12 | 1.000 | 0.89 | 1.000 | Weekly usage of mobile phone in last 4 months | Lifestyles | |
| X1120 | 0.96 | 1.000 | 1.12 | 1.000 | 1.08 | 1.000 | 1.08 | 1.000 | 1.12 | 1.000 | 0.85 | 1.000 | Weekly usage of mobile phone in last 4 months | Lifestyles | |
| X1120 | 1.00 | 1.000 | 0.90 | 1.000 | 1.45 | 1.000 | 1.45 | 1.000 | 0.90 | 1.000 | 0.88 | 1.000 | Weekly usage of mobile phone in last 4 months | Lifestyles | |
| X1120 | 0.94 | 1.000 | 1.07 | 1.000 | 1.34 | 1.000 | 1.34 | 1.000 | 1.07 | 1.000 | 0.75 | 1.000 | Weekly usage of mobile phone in last 4 months | Lifestyles | |
| X1120 | 0.85 | 1.000 | 0.88 | 1.000 | 0.87 | 1.000 | 0.87 | 1.000 | 0.88 | 1.000 | 0.82 | 1.000 | Weekly usage of mobile phone in last 4 months | Lifestyles | |
| X1130 | 0.75 | 1.000 | 0.67 | 1.000 | 0.80 | 1.000 | 0.80 | 1.000 | 0.67 | 1.000 | 0.77 | 1.000 | Hands -free device/speakerphone use with mobile phone in last 3 month | Lifestyles | |
| X1130 | 0.46 | 0.156 | 0.56 | 1.000 | 0.49 | 1.000 | 0.49 | 1.000 | 0.56 | 1.000 | 0.39 | 1.000 | Hands -free device/speakerphone use with mobile phone in last 3 month | Lifestyles | |
| X1130 | 0.79 | 1.000 | 0.57 | 1.000 | 0.89 | 1.000 | 0.89 | 1.000 | 0.57 | 1.000 | 0.86 | 1.000 | Hands -free device/speakerphone use with mobile phone in last 3 month | Lifestyles | |
| X1130 | 0.96 | 1.000 | 1.14 | 1.000 | 0.53 | 1.000 | 0.53 | 1.000 | 1.14 | 1.000 | 1.04 | 1.000 | Hands -free device/speakerphone use with mobile phone in last 3 month | Lifestyles | |
| Sleep_level | 1.77 | 9.66×10^-21^ | 1.65 | 0.002 | 1.57 | 0.095 | 1.57 | 0.095 | 1.65 | 0.002 | 1.92 | 5.96×10^-14^ | Sleep duration | Lifestyles | |
| X1190 | 1.55 | 1.53×10^-10^ | 1.68 | 0.003 | 1.37 | 1.000 | 1.37 | 1.000 | 1.68 | 0.003 | 1.57 | 3.00×10^-05^ | Nap during day | Lifestyles | |
| X1190 | 2.43 | 5.58×10^-16^ | 3.24 | 1.56×10^-08^ | 1.46 | 1.000 | 1.46 | 1.000 | 3.24 | 1.56×10^-08^ | 2.47 | 3.01×10^-08^ | Nap during day | Lifestyles | |
| X1200 | 1.12 | 1.000 | 1.24 | 1.000 | 0.92 | 1.000 | 0.92 | 1.000 | 1.24 | 1.000 | 1.16 | 1.000 | Sleeplessness / insomnia | Lifestyles | |
| X1200 | 1.43 | 0.003 | 1.56 | 1.000 | 1.12 | 1.000 | 1.12 | 1.000 | 1.56 | 1.000 | 1.52 | 0.070 | Sleeplessness / insomnia | Lifestyles | |
| X1210 | 1.23 | 0.245 | 1.20 | 1.000 | 1.26 | 1.000 | 1.26 | 1.000 | 1.20 | 1.000 | 1.23 | 1.000 | Snoring | Lifestyles | |
| X1220 | 1.49 | 2.70×10^-07^ | - | - | - | - | - | - | - | - | 1.57 | 1.37×10^-04^ | Daytime dozing / sleeping (narcolepsy) | Lifestyles | |
| X1220 | 2.50 | 3.22×10^-12^ | - | - | - | - | - | - | - | - | 2.87 | 1.61×10^-09^ | Daytime dozing / sleeping (narcolepsy) | Lifestyles | |
| X1220 | 11.96 | 1.000 | - | - | - | - | - | - | - | - | 23.91 | 0.358 | Daytime dozing / sleeping (narcolepsy) | Lifestyles | |
| X1269 | 1.83 | 1.23×10^-08^ | 1.77 | 0.457 | 2.13 | 0.027 | 2.13 | 0.026 | 1.77 | 0.455 | 1.75 | 0.003 | Exposure to tobacco smoke at home | Lifestyles | |
| X1279 | 1.52 | 6.68×10^-09^ | 1.51 | 0.211 | 1.67 | 0.036 | 1.67 | 0.035 | 1.51 | 0.210 | 1.47 | 0.003 | Exposure to tobacco smoke outside home | Lifestyles | |
| X2159 | 1.60 | 0.053 | 1.17 | 1.000 | 1.66 | 1.000 | 1.66 | 1.000 | 1.17 | 1.000 | 1.77 | 0.101 | Ever had sam×10-sex intercourse | Lifestyles | |
| X22036 | 1.29 | 0.033 | 1.40 | 1.000 | 1.35 | 1.000 | 1.35 | 1.000 | 1.40 | 1.000 | 1.21 | 1.000 | Below moderate/vigorous/walking recommendation | Lifestyles | |
| X6155_0 | 0.77 | 0.003 | 0.73 | 1.000 | 0.71 | 1.000 | 0.71 | 1.000 | 0.73 | 1.000 | 0.82 | 1.000 | No vitamin and mineral supplements | Lifestyles | |
| X6155_1 | 1.86 | 0.006 | 1.46 | 1.000 | 1.85 | 1.000 | 1.85 | 1.000 | 1.46 | 1.000 | 2.06 | 0.032 | Vitamin A supplements | Lifestyles | |
| X6155_2 | 1.81 | 8.64×10^-06^ | 1.75 | 1.000 | 1.79 | 1.000 | 1.79 | 1.000 | 1.75 | 1.000 | 1.85 | 0.005 | Vitamin B supplements | Lifestyles | |
| X6155_3 | 1.25 | 1.000 | 1.07 | 1.000 | 1.11 | 1.000 | 1.11 | 1.000 | 1.07 | 1.000 | 1.39 | 1.000 | Vitamin C supplements | Lifestyles | |
| X6155_4 | 1.50 | 0.202 | 1.10 | 1.000 | 1.16 | 1.000 | 1.16 | 1.000 | 1.10 | 1.000 | 1.82 | 0.018 | Vitamin D supplements | Lifestyles | |
| X6155_5 | 1.38 | 1.000 | 1.24 | 1.000 | 0.80 | 1.000 | 0.80 | 1.000 | 1.24 | 1.000 | 1.65 | 1.000 | Vitamin E supplements | Lifestyles | |
| X6155_6 | 1.65 | 0.236 | 1.69 | 1.000 | 1.65 | 1.000 | 1.65 | 1.000 | 1.69 | 1.000 | 1.63 | 1.000 | Folic acid or Folate (Vit B9) supplements | Lifestyles | |
| X6155_7 | 1.15 | 1.000 | 1.34 | 1.000 | 1.18 | 1.000 | 1.18 | 1.000 | 1.34 | 1.000 | 1.05 | 1.000 | Multivitamins +/- minerals supplements | Lifestyles | |
| X6179_0 | 0.98 | 1.000 | 0.97 | 1.000 | 0.91 | 1.000 | 0.91 | 1.000 | 0.97 | 1.000 | 1.01 | 1.000 | No mineral or other dietary supplements | Lifestyles | |
| X6179_1 | 0.96 | 1.000 | 0.94 | 1.000 | 0.98 | 1.000 | 0.98 | 1.000 | 0.94 | 1.000 | 0.95 | 1.000 | Fish oil (including cod liver oil) supplements | Lifestyles | |
| X6179_2 | 0.65 | 4.01×10^-05^ | 0.70 | 1.000 | 0.60 | 1.000 | 0.60 | 1.000 | 0.70 | 1.000 | 0.64 | 0.051 | Glucosamine supplements | Lifestyles | |
| X6179_3 | 1.48 | 0.015 | 1.52 | 1.000 | 1.20 | 1.000 | 1.20 | 1.000 | 1.52 | 1.000 | 1.58 | 0.139 | Calcium supplements | Lifestyles | |
| X6179_4 | 1.23 | 1.000 | 1.14 | 1.000 | 1.63 | 1.000 | 1.63 | 1.000 | 1.14 | 1.000 | 1.15 | 1.000 | Zinc supplements | Lifestyles | |
| X6179_5 | 2.06 | 1.19×10^-07^ | 1.87 | 1.000 | 2.31 | 0.265 | 2.31 | 0.264 | 1.87 | 1.000 | 2.06 | 0.001 | Iron supplements | Lifestyles | |
| X6179_6 | 1.34 | 1.000 | 1.57 | 1.000 | 1.30 | 1.000 | 1.30 | 1.000 | 1.57 | 1.000 | 1.24 | 1.000 | Selenium supplements | Lifestyles | |
| Procesdd_meat | 1.21 | 0.477 | 1.11 | 1.000 | 1.06 | 1.000 | 1.06 | 1.000 | 1.11 | 1.000 | 1.34 | 0.183 | Processed meat intake reduced | Lifestyles | |
| Healthy_diet_level | 0.79 | 0.157 | 0.84 | 1.000 | 0.84 | 1.000 | 0.84 | 1.000 | 0.84 | 1.000 | 0.76 | 0.769 | Healthy diet score | Lifestyles | |
| Healthy_diet_level | 0.75 | 0.732 | 0.68 | 1.000 | 1.09 | 1.000 | 1.09 | 1.000 | 0.68 | 1.000 | 0.66 | 0.756 | Healthy diet score | Lifestyles | |
| X1050 | 0.96 | 1.000 | 1.12 | 1.000 | 0.90 | 1.000 | 0.90 | 1.000 | 1.12 | 1.000 | 0.91 | 1.000 | Time spend outdoors in summer | Lifestyles | |
| X1050 | 1.08 | 1.000 | 0.97 | 1.000 | 1.14 | 1.000 | 1.14 | 1.000 | 0.97 | 1.000 | 1.10 | 1.000 | Time spend outdoors in summer | Lifestyles | |
| X1060 | 1.12 | 1.000 | 1.06 | 1.000 | 1.34 | 1.000 | 1.34 | 1.000 | 1.06 | 1.000 | 1.07 | 1.000 | Time spent outdoors in winter | Lifestyles | |
| X1060 | 1.45 | 9.08×10^-06^ | 1.25 | 1.000 | 1.70 | 0.067 | 1.70 | 0.067 | 1.25 | 1.000 | 1.47 | 0.010 | Time spent outdoors in winter | Lifestyles | |
| X1070 | 1.00 | 1.000 | 1.06 | 1.000 | 0.95 | 1.000 | 0.95 | 1.000 | 1.06 | 1.000 | 1.00 | 1.000 | Time spent watching television (TV) | Lifestyles | |
| X1070 | 1.62 | 7.18×10^-11^ | 1.73 | 0.003 | 1.40 | 1.000 | 1.40 | 1.000 | 1.73 | 0.003 | 1.66 | 9.07×10^-06^ | Time spent watching television (TV) | Lifestyles | |
| X1080 | 0.61 | 2.40×10^-09^ | 0.85 | 1.000 | 0.49 | 0.004 | 0.49 | 0.004 | 0.85 | 1.000 | 0.56 | 2.26×10^-06^ | Time spent using computer | Lifestyles | |
| X1080 | 0.70 | 4.52×10^-04^ | 0.89 | 1.000 | 0.71 | 1.000 | 0.71 | 1.000 | 0.89 | 1.000 | 0.60 | 0.001 | Time spent using computer | Lifestyles | |
| X1488 | 0.90 | 1.000 | 0.85 | 1.000 | 1.07 | 1.000 | 1.07 | 1.000 | 0.85 | 1.000 | 0.86 | 1.000 | Tea intake | Lifestyles | |
| X1488 | 1.05 | 1.000 | 1.09 | 1.000 | 1.08 | 1.000 | 1.08 | 1.000 | 1.09 | 1.000 | 1.02 | 1.000 | Tea intake | Lifestyles | |
| X1528 | 1.06 | 1.000 | 1.00 | 1.000 | 0.97 | 1.000 | 0.97 | 1.000 | 1.00 | 1.000 | 1.14 | 1.000 | Water intake | Lifestyles | |
| X1528 | 1.23 | 0.427 | 1.31 | 1.000 | 1.33 | 1.000 | 1.33 | 1.000 | 1.31 | 1.000 | 1.15 | 1.000 | Water intake | Lifestyles | |
| X2139 | 0.82 | 1.000 | 0.80 | 1.000 | 0.69 | 1.000 | 0.69 | 1.000 | 0.80 | 1.000 | 0.90 | 1.000 | Age first had sexual intercourse | Lifestyles | |
| X2139 | 0.93 | 1.000 | 1.03 | 1.000 | 0.79 | 1.000 | 0.79 | 1.000 | 1.03 | 1.000 | 0.93 | 1.000 | Age first had sexual intercourse | Lifestyles | |
| X2149 | 1.13 | 1.000 | 1.33 | 1.000 | 1.00 | 1.000 | 1.00 | 1.000 | 1.33 | 1.000 | 1.10 | 1.000 | Lifetime number of sexual partners | Lifestyles | |
| X2149 | 1.24 | 0.566 | 1.37 | 1.000 | 1.16 | 1.000 | 1.16 | 1.000 | 1.37 | 1.000 | 1.21 | 1.000 | Lifetime number of sexual partners | Lifestyles | |
| X22037 | 0.90 | 1.000 | 0.80 | 1.000 | 0.99 | 1.000 | 0.99 | 1.000 | 0.80 | 1.000 | 0.92 | 1.000 | MET minutes per week for walking | Lifestyles | |
| X22037 | 0.90 | 1.000 | 0.74 | 1.000 | 1.08 | 1.000 | 1.08 | 1.000 | 0.74 | 1.000 | 0.93 | 1.000 | MET minutes per week for walking | Lifestyles | |
| X22038 | 0.94 | 1.000 | 1.01 | 1.000 | 0.89 | 1.000 | 0.89 | 1.000 | 1.01 | 1.000 | 0.93 | 1.000 | MET minutes per week for moderate activity | Lifestyles | |
| X22038 | 0.98 | 1.000 | 0.95 | 1.000 | 1.10 | 1.000 | 1.10 | 1.000 | 0.95 | 1.000 | 0.94 | 1.000 | MET minutes per week for moderate activity | Lifestyles | |
| X22039 | 0.72 | 6.23×10^-04^ | 0.57 | 0.019 | 0.93 | 1.000 | 0.93 | 1.000 | 0.57 | 0.019 | 0.72 | 0.207 | MET minutes per week for vigorous activity | Lifestyles | |
| X22039 | 0.72 | 2.24×10^-04^ | 0.67 | 0.413 | 0.72 | 1.000 | 0.72 | 1.000 | 0.67 | 0.412 | 0.74 | 0.450 | MET minutes per week for vigorous activity | Lifestyles | |
| X1498 | 0.96 | 1.000 | 0.92 | 1.000 | 0.81 | 1.000 | 0.81 | 1.000 | 0.92 | 1.000 | 1.06 | 1.000 | Coffee intake | Lifestyles | |
| X1498 | 0.94 | 1.000 | 0.90 | 1.000 | 0.81 | 1.000 | 0.81 | 1.000 | 0.90 | 1.000 | 1.02 | 1.000 | Coffee intake | Lifestyles | |
| X22040 | 0.82 | 1.000 | 0.85 | 1.000 | 0.92 | 1.000 | 0.92 | 1.000 | 0.85 | 1.000 | 0.77 | 1.000 | Summed MET minutes per week for all activity | Lifestyles | |
| X22040 | 0.86 | 1.000 | 0.79 | 1.000 | 0.96 | 1.000 | 0.96 | 1.000 | 0.79 | 1.000 | 0.86 | 1.000 | Summed MET minutes per week for all activity | Lifestyles | |
| X6160_0 | 1.17 | 1.000 | 1.11 | 1.000 | 1.20 | 1.000 | 1.20 | 1.000 | 1.11 | 1.000 | 1.18 | 1.000 | Leisure/social activities | Lifestyles | |
| X6160_1 | 0.59 | 1.50×10^-11^ | 0.63 | 0.108 | 0.53 | 0.015 | 0.53 | 0.015 | 0.63 | 0.107 | 0.60 | 3.60×10^-05^ | Leisure/social activities | Lifestyles | |
| X6160_2 | 0.97 | 1.000 | 1.00 | 1.000 | 1.10 | 1.000 | 1.10 | 1.000 | 1.00 | 1.000 | 0.91 | 1.000 | Leisure/social activities | Lifestyles | |
| X6160_3 | 1.17 | 1.000 | 1.25 | 1.000 | 0.93 | 1.000 | 0.93 | 1.000 | 1.25 | 1.000 | 1.23 | 1.000 | Leisure/social activities | Lifestyles | |
| X6160_4 | 1.45 | 0.019 | 1.42 | 1.000 | 1.27 | 1.000 | 1.27 | 1.000 | 1.42 | 1.000 | 1.54 | 0.194 | Leisure/social activities | Lifestyles | |
| X6160_5 | 0.96 | 1.000 | 1.27 | 1.000 | 0.95 | 1.000 | 0.95 | 1.000 | 1.27 | 1.000 | 0.82 | 1.000 | Leisure/social activities | Lifestyles | |
| X1031 | 0.23 | 2.09×10^-39^ | 0.25 | 1.19×10^-07^ | 0.17 | 2.17×10^-12^ | 0.17 | 2.17×10^-12^ | 0.25 | 1.19×10^-07^ | 0.24 | 1.03×10^-18^ | frequency of friendfamily visits | Lifestyles | |
| Vegetable | 0.75 | 0.010 | 0.78 | 1.000 | 0.75 | 1.000 | 0.75 | 1.000 | 0.78 | 1.000 | 0.74 | 0.574 | vegetable | Lifestyles | |
| Vegetable | 0.86 | 1.000 | 0.73 | 1.000 | 0.89 | 1.000 | 0.89 | 1.000 | 0.73 | 1.000 | 0.91 | 1.000 | vegetable | Lifestyles | |
| Red_meat | 1.09 | 1.000 | 1.25 | 1.000 | 1.11 | 1.000 | 1.11 | 1.000 | 1.25 | 1.000 | 1.00 | 1.000 | Red meat intake reudced | Lifestyles | |
| White_bread_refined_cereals | 1.24 | 1.000 | 1.20 | 1.000 | 1.17 | 1.000 | 1.17 | 1.000 | 1.20 | 1.000 | 1.30 | 1.000 | White bread/refined cereals intake reduced | Lifestyles | |
| X24006 | 1.23 | 1.000 | 0.98 | 1.000 | 1.46 | 1.000 | 1.46 | 1.000 | 0.98 | 1.000 | 1.30 | 1.000 | Particulate matter air pollution (pm2.5) | Local environment | |
| X24006 | 1.78 | 6.14×10^-14^ | 1.34 | 1.000 | 1.95 | 0.005 | 1.95 | 0.005 | 1.34 | 1.000 | 2.01 | 9.18×10^-10^ | Particulate matter air pollution (pm2.5) | Local environment | |
| X24008 | 1.27 | 0.146 | 1.17 | 1.000 | 1.40 | 1.000 | 1.40 | 1.000 | 1.17 | 1.000 | 1.28 | 1.000 | Particulate matter air pollution 2.5-10um | Local environment | |
| X24008 | 1.22 | 1.000 | 1.11 | 1.000 | 1.34 | 1.000 | 1.34 | 1.000 | 1.11 | 1.000 | 1.23 | 1.000 | Particulate matter air pollution 2.5-10um | Local environment | |
| X24020 | 1.06 | 1.000 | 0.97 | 1.000 | 1.10 | 1.000 | 1.10 | 1.000 | 0.97 | 1.000 | 1.10 | 1.000 | Average daytime sound level of noise pollution | Local environment | |
| X24020 | 1.14 | 1.000 | 0.94 | 1.000 | 0.98 | 1.000 | 0.98 | 1.000 | 0.94 | 1.000 | 1.33 | 0.851 | Average daytime sound level of noise pollution | Local environment | |
| X24021 | 1.07 | 1.000 | 0.98 | 1.000 | 1.12 | 1.000 | 1.12 | 1.000 | 0.98 | 1.000 | 1.10 | 1.000 | Average evening sound level of noise pollution | Local environment | |
| X24021 | 1.11 | 1.000 | 0.92 | 1.000 | 0.99 | 1.000 | 0.99 | 1.000 | 0.92 | 1.000 | 1.28 | 1.000 | Average evening sound level of noise pollution | Local environment | |
| X24022 | 1.07 | 1.000 | 0.99 | 1.000 | 1.12 | 1.000 | 1.12 | 1.000 | 0.99 | 1.000 | 1.09 | 1.000 | Average night-time sound level of noise pollution | Local environment | |
| X24022 | 1.11 | 1.000 | 0.92 | 1.000 | 0.99 | 1.000 | 0.99 | 1.000 | 0.92 | 1.000 | 1.28 | 1.000 | Average night-time sound level of noise pollution | Local environment | |
| X24500 | 0.85 | 1.000 | 0.98 | 1.000 | 0.84 | 1.000 | 0.84 | 1.000 | 0.98 | 1.000 | 0.80 | 1.000 | Greenspace percentage, buffer 1000m | Local environment | |
| X24500 | 0.56 | 4.94×10^-13^ | 0.65 | 0.439 | 0.51 | 0.006 | 0.51 | 0.006 | 0.65 | 0.438 | 0.54 | 6.71×10^-07^ | Greenspace percentage, buffer 1000m | Local environment | |
| X24501 | 1.03 | 1.000 | 1.28 | 1.000 | 0.98 | 1.000 | 0.98 | 1.000 | 1.28 | 1.000 | 0.93 | 1.000 | Domestic garden percentage, buffer 1000m | Local environment | |
| X24501 | 0.97 | 1.000 | 1.09 | 1.000 | 1.09 | 1.000 | 1.09 | 1.000 | 1.09 | 1.000 | 0.87 | 1.000 | Domestic garden percentage, buffer 1000m | Local environment | |
| X24502 | 0.88 | 1.000 | 0.84 | 1.000 | 0.74 | 1.000 | 0.74 | 1.000 | 0.84 | 1.000 | 0.98 | 1.000 | Water percentage, buffer 1000m | Local environment | |
| X24502 | 1.06 | 1.000 | 1.11 | 1.000 | 0.85 | 1.000 | 0.85 | 1.000 | 1.11 | 1.000 | 1.14 | 1.000 | Water percentage, buffer 1000m | Local environment | |
| X24503 | 1.02 | 1.000 | 1.11 | 1.000 | 1.10 | 1.000 | 1.10 | 1.000 | 1.11 | 1.000 | 0.95 | 1.000 | Greenspace percentage, buffer 300m | Local environment | |
| X24503 | 0.69 | 7.59×10^-05^ | 0.84 | 1.000 | 0.64 | 1.000 | 0.64 | 1.000 | 0.84 | 1.000 | 0.64 | 0.003 | Greenspace percentage, buffer 300m | Local environment | |
| X24504 | 0.92 | 1.000 | 1.11 | 1.000 | 0.91 | 1.000 | 0.91 | 1.000 | 1.11 | 1.000 | 0.84 | 1.000 | Domestic garden percentage, buffer 300m | Local environment | |
| X24504 | 0.82 | 1.000 | 0.80 | 1.000 | 0.86 | 1.000 | 0.86 | 1.000 | 0.80 | 1.000 | 0.81 | 1.000 | Domestic garden percentage, buffer 300m | Local environment | |
| X24505 | 0.82 | 1.000 | 0.72 | 1.000 | 0.80 | 1.000 | 0.80 | 1.000 | 0.72 | 1.000 | 0.88 | 1.000 | Water percentage, buffer 300m | Local environment | |
| X24505 | 0.96 | 1.000 | 0.96 | 1.000 | 0.88 | 1.000 | 0.88 | 1.000 | 0.96 | 1.000 | 1.01 | 1.000 | Water percentage, buffer 300m | Local environment | |
| X24506 | 0.80 | 0.162 | 0.80 | 1.000 | 1.04 | 1.000 | 1.04 | 1.000 | 0.80 | 1.000 | 0.72 | 0.075 | Natural environment percentage, buffer 1000m | Local environment | |
| X24506 | 0.53 | 2.23×10^-16^ | 0.60 | 0.048 | 0.53 | 0.020 | 0.53 | 0.020 | 0.60 | 0.048 | 0.49 | 1.16×10^-09^ | Natural environment percentage, buffer 1000m | Local environment | |
| X24507 | 0.94 | 1.000 | 0.91 | 1.000 | 1.11 | 1.000 | 1.11 | 1.000 | 0.91 | 1.000 | 0.90 | 1.000 | Natural environment percentage, buffer 300m | Local environment | |
| X24507 | 0.68 | 2.07×10^-05^ | 0.82 | 1.000 | 0.80 | 1.000 | 0.80 | 1.000 | 0.82 | 1.000 | 0.57 | 1.77×10^-05^ | Natural environment percentage, buffer 300m | Local environment | |
| NOx | 1.44 | 4.56×10^-04^ | 1.19 | 1.000 | 1.48 | 1.000 | 1.48 | 1.000 | 1.19 | 1.000 | 1.59 | 0.007 | Nitrogen oxides | Local environment | |
| NOx | 1.96 | 2.20×10^-18^ | 1.76 | 0.005 | 1.92 | 0.006 | 1.92 | 0.006 | 1.76 | 0.005 | 2.11 | 1.87×10^-10^ | Nitrogen oxides | Local environment | |
| X24023 | 1.06 | 1.000 | 0.99 | 1.000 | 1.11 | 1.000 | 1.11 | 1.000 | 0.99 | 1.000 | 1.09 | 1.000 | Average 16-hour sound level of noise pollution | Local environment | |
| X24023 | 1.11 | 1.000 | 0.92 | 1.000 | 0.99 | 1.000 | 0.99 | 1.000 | 0.92 | 1.000 | 1.28 | 1.000 | Average 16-hour sound level of noise pollution | Local environment | |
| X24024 | 1.06 | 1.000 | 0.99 | 1.000 | 1.11 | 1.000 | 1.11 | 1.000 | 0.99 | 1.000 | 1.09 | 1.000 | Average 24-hour sound level of noise pollution | Local environment | |
| X24024 | 1.11 | 1.000 | 0.92 | 1.000 | 0.99 | 1.000 | 0.99 | 1.000 | 0.92 | 1.000 | 1.28 | 1.000 | Average 24-hour sound level of noise pollution | Local environment | |
| PM10 | 1.38 | 0.005 | 1.27 | 1.000 | 1.52 | 1.000 | 1.52 | 1.000 | 1.27 | 1.000 | 1.38 | 0.822 | Particulate matter air pollution (pm10) | Local environment | |
| PM10 | 1.77 | 2.77×10^-13^ | 1.55 | 0.294 | 1.62 | 0.549 | 1.62 | 0.547 | 1.55 | 0.293 | 1.96 | 6.18×10^-09^ | Particulate matter air pollution (pm10) | Local environment | |
| Arthritis | 1.29 | 0.345 | 1.33 | 1.000 | 1.44 | 1.000 | 1.44 | 1.000 | 1.33 | 1.000 | 1.20 | 1.000 | Arthritis | Medical history | |
| CHD | 1.59 | 0.003 | 1.60 | 1.000 | 1.61 | 1.000 | 1.61 | 1.000 | 1.60 | 1.000 | 1.57 | 0.769 | CHD | Medical history | |
| COPD | 2.26 | 2.57×10^-06^ | 2.61 | 0.064 | 2.85 | 0.074 | 2.85 | 0.074 | 2.61 | 0.064 | 1.89 | 0.737 | COPD | Medical history | |
| Diabetes | 1.90 | 2.77×10^-08^ | 1.84 | 0.418 | 2.06 | 0.098 | 2.06 | 0.098 | 1.84 | 0.416 | 1.85 | 0.004 | Diabetes | Medical history | |
| Hear_loss | 0.88 | 1.000 | 0.98 | 1.000 | 0.85 | 1.000 | 0.85 | 1.000 | 0.98 | 1.000 | 0.84 | 1.000 | Hearing_loss | Medical history | |
| Hepertension | 0.98 | 1.000 | 1.13 | 1.000 | 0.84 | 1.000 | 0.84 | 1.000 | 1.13 | 1.000 | 0.97 | 1.000 | Hypertension | Medical history | |
| X6146 | 6.08 | <2×10^-16^ | 6.73 | 7.51×10^-51^ | 5.47 | 4.14×10^-27^ | 5.47 | 4.12×10^-27^ | 6.73 | 7.49×10^-51^ | 6.00 | 7.52×10^-81^ | Disability | Medical history | |
| X6149_0 | 0.60 | 1.37×10^-17^ | 0.66 | 0.038 | 0.55 | 1.72×10^-04^ | 0.55 | 1.72×10^-04^ | 0.66 | 0.038 | 0.59 | 3.49×10^-09^ | Mouth/teeth dental problems | Medical history | |
| X6149_1 | 1.11 | 1.000 | 1.19 | 1.000 | 1.09 | 1.000 | 1.09 | 1.000 | 1.19 | 1.000 | 1.07 | 1.000 | Mouth/teeth dental problems | Medical history | |
| X6149_2 | 2.34 | 7.30×10^-12^ | 2.93 | 1.62×10^-05^ | 1.94 | 1.000 | 1.94 | 1.000 | 2.93 | 1.62×10^-05^ | 2.20 | 1.04×10^-04^ | Mouth/teeth dental problems | Medical history | |
| X6149_3 | 1.24 | 1.000 | 1.45 | 1.000 | 1.14 | 1.000 | 1.14 | 1.000 | 1.45 | 1.000 | 1.18 | 1.000 | Mouth/teeth dental problems | Medical history | |
| X6149_4 | 1.78 | 1.37×10^-05^ | 1.23 | 1.000 | 1.51 | 1.000 | 1.51 | 1.000 | 1.23 | 1.000 | 2.16 | 2.03×10^-06^ | Mouth/teeth dental problems | Medical history | |
| X6149_5 | 2.10 | 1.24×10^-11^ | 2.24 | 0.005 | 1.50 | 1.000 | 1.50 | 1.000 | 2.24 | 0.004 | 2.26 | 1.22×10^-07^ | Mouth/teeth dental problems | Medical history | |
| X6149_6 | 1.70 | 4.91×10^-12^ | 1.29 | 1.000 | 2.02 | 2.22×10^-04^ | 2.02 | 2.21×10^-04^ | 1.29 | 1.000 | 1.80 | 2.86×10^-07^ | Mouth/teeth dental problems | Medical history | |
| X130176 | 0.31 | 3.01×10^-05^ | 0.32 | 1.000 | 0.27 | 1.000 | 0.27 | 1.000 | 0.32 | 1.000 | 0.32 | 0.059 | varicella [chickenpox] | Medical history | |
| X130178 | 0.49 | 1.000 | 0.43 | 1.000 | 0.88 | 1.000 | 0.88 | 1.000 | 0.43 | 1.000 | 0.33 | 1.000 | zoster [herpes zoster] | Medical history | |
| X130188 | 0.79 | 1.000 | 0.84 | 1.000 | 1.07 | 1.000 | 1.07 | 1.000 | 0.84 | 1.000 | 0.65 | 1.000 | viral warts | Medical history | |
| X130226 | 1.01 | 1.000 | 0.94 | 1.000 | 1.08 | 1.000 | 1.08 | 1.000 | 0.94 | 1.000 | 1.02 | 1.000 | dermatophytosis | Medical history | |
| X130622 | 1.33 | 1.000 | 1.61 | 1.000 | 1.50 | 1.000 | 1.50 | 1.000 | 1.61 | 1.000 | 1.12 | 1.000 | iron deficiency anaemia | Medical history | |
| X130648 | 1.81 | 0.065 | 1.33 | 1.000 | 3.06 | 0.008 | 3.06 | 0.008 | 1.33 | 1.000 | 1.52 | 1.000 | other anaemias | Medical history | |
| X130696 | 1.49 | 0.067 | 1.56 | 1.000 | 1.54 | 1.000 | 1.54 | 1.000 | 1.56 | 1.000 | 1.43 | 1.000 | other hypothyroidism | Medical history | |
| X130792 | 1.25 | 1.000 | 0.96 | 1.000 | 1.25 | 1.000 | 1.25 | 1.000 | 0.96 | 1.000 | 1.40 | 1.000 | obesity | Medical history | |
| X130814 | 1.34 | 0.030 | 1.38 | 1.000 | 1.26 | 1.000 | 1.26 | 1.000 | 1.38 | 1.000 | 1.35 | 1.000 | disorders of lipoprotein metabolism and other lipidaemias | Medical history | |
| X131052 | 0.91 | 1.000 | 1.05 | 1.000 | 1.01 | 1.000 | 1.01 | 1.000 | 1.05 | 1.000 | 0.79 | 1.000 | migraine | Medical history | |
| X131060 | 1.64 | 0.629 | 2.01 | 1.000 | 0.77 | 1.000 | 0.77 | 1.000 | 2.01 | 1.000 | 1.80 | 1.000 | sleep disorders | Medical history | |
| X131074 | 0.92 | 1.000 | 1.09 | 1.000 | 0.97 | 1.000 | 0.97 | 1.000 | 1.09 | 1.000 | 0.80 | 1.000 | mononeuropathies of upper limb | Medical history | |
| X131128 | 0.84 | 1.000 | 0.55 | 1.000 | 1.43 | 1.000 | 1.43 | 1.000 | 0.55 | 1.000 | 0.76 | 1.000 | hordeolum and chalazion | Medical history | |
| X131142 | 1.11 | 1.000 | 0.88 | 1.000 | 1.64 | 1.000 | 1.64 | 1.000 | 0.88 | 1.000 | 1.00 | 1.000 | conjunctivitis | Medical history | |
| X131166 | 1.50 | 1.000 | 1.32 | 1.000 | 1.35 | 1.000 | 1.35 | 1.000 | 1.32 | 1.000 | 1.67 | 1.000 | other cataract | Medical history | |
| X131222 | 0.92 | 1.000 | 1.48 | 1.000 | 0.97 | 1.000 | 0.97 | 1.000 | 1.48 | 1.000 | 0.62 | 1.000 | otitis externa | Medical history | |
| X131224 | 0.78 | 1.000 | 0.96 | 1.000 | 0.42 | 1.000 | 0.42 | 1.000 | 0.96 | 1.000 | 0.83 | 1.000 | other disorders of external ear | Medical history | |
| X131262 | 1.01 | 1.000 | 1.45 | 1.000 | 1.45 | 1.000 | 1.45 | 1.000 | 1.45 | 1.000 | 0.60 | 1.000 | otalgia and effusion of ear | Medical history | |
| X131264 | 1.11 | 1.000 | 1.58 | 1.000 | 1.02 | 1.000 | 1.02 | 1.000 | 1.58 | 1.000 | 0.89 | 1.000 | other disorders of ear, not elsewhere classified | Medical history | |
| X131350 | 1.27 | 1.000 | 0.95 | 1.000 | 1.72 | 1.000 | 1.72 | 1.000 | 0.95 | 1.000 | 1.26 | 1.000 | atrial fibrillation and flutter | Medical history | |
| X131396 | 1.68 | 0.101 | 1.29 | 1.000 | 1.66 | 1.000 | 1.66 | 1.000 | 1.29 | 1.000 | 1.91 | 0.256 | phlebitis and thrombophlebitis | Medical history | |
| X131402 | 0.91 | 1.000 | 1.08 | 1.000 | 0.71 | 1.000 | 0.71 | 1.000 | 1.08 | 1.000 | 0.91 | 1.000 | varicose veins of lower extremities | Medical history | |
| X131404 | 1.36 | 1.000 | 1.20 | 1.000 | 1.74 | 1.000 | 1.74 | 1.000 | 1.20 | 1.000 | 1.30 | 1.000 | haemorrhoids | Medical history | |
| X131426 | 0.86 | 1.000 | 0.86 | 1.000 | 0.98 | 1.000 | 0.98 | 1.000 | 0.86 | 1.000 | 0.81 | 1.000 | acute sinusitis | Medical history | |
| X131428 | 0.79 | 1.000 | 1.18 | 1.000 | 0.59 | 1.000 | 0.59 | 1.000 | 1.18 | 1.000 | 0.69 | 1.000 | acute pharyngitis | Medical history | |
| X131430 | 0.57 | 1.000 | 0.54 | 1.000 | 0.19 | 1.000 | 0.19 | 1.000 | 0.54 | 1.000 | 0.74 | 1.000 | acute tonsillitis | Medical history | |
| X131436 | 1.10 | 1.000 | 1.06 | 1.000 | 1.34 | 1.000 | 1.34 | 1.000 | 1.06 | 1.000 | 1.03 | 1.000 | acute upper respiratory infections of multiple and unspecified sites | Medical history | |
| X131442 | 0.68 | 1.000 | 1.14 | 1.000 | 0.45 | 1.000 | 0.45 | 1.000 | 1.14 | 1.000 | 0.55 | 1.000 | influenza, virus not identified | Medical history | |
| X131456 | 1.41 | 1.000 | 1.25 | 1.000 | 1.56 | 1.000 | 1.56 | 1.000 | 1.25 | 1.000 | 1.43 | 1.000 | pneumonia, organism unspecified | Medical history | |
| X131462 | 1.29 | 1.000 | 1.27 | 1.000 | 1.36 | 1.000 | 1.36 | 1.000 | 1.27 | 1.000 | 1.27 | 1.000 | unspecified acute lower respiratory infection | Medical history | |
| X131464 | 0.90 | 1.000 | 1.09 | 1.000 | 1.14 | 1.000 | 1.14 | 1.000 | 1.09 | 1.000 | 0.71 | 1.000 | vasomotor and allergic rhinitis | Medical history | |
| X131468 | 0.75 | 1.000 | 1.26 | 1.000 | 1.24 | 1.000 | 1.24 | 1.000 | 1.26 | 1.000 | 0.31 | 1.000 | chronic sinusitis | Medical history | |
| X131472 | 1.15 | 1.000 | 1.18 | 1.000 | 0.77 | 1.000 | 0.77 | 1.000 | 1.18 | 1.000 | 1.28 | 1.000 | other disorders of nose and nasal sinuses | Medical history | |
| X131494 | 1.51 | 1.55×10^-05^ | 1.64 | 0.128 | 1.65 | 0.444 | 1.65 | 0.443 | 1.64 | 0.128 | 1.38 | 0.763 | asthma | Medical history | |
| X131584 | 1.32 | 1.000 | 1.81 | 0.058 | 1.14 | 1.000 | 1.14 | 1.000 | 1.81 | 0.057 | 1.15 | 1.000 | gastro -oesophageal reflux Medical history | Medical history | |
| X131598 | 1.81 | 3.05×10^-06^ | 1.59 | 1.000 | 2.07 | 0.184 | 2.07 | 0.184 | 1.59 | 1.000 | 1.82 | 0.010 | gastritis and duodenitis | Medical history | |
| X131600 | 1.61 | 0.722 | 1.67 | 1.000 | 1.98 | 1.000 | 1.98 | 1.000 | 1.67 | 1.000 | 1.42 | 1.000 | dyspepsia | Medical history | |
| X131612 | 0.85 | 1.000 | 0.71 | 1.000 | 0.61 | 1.000 | 0.61 | 1.000 | 0.71 | 1.000 | 1.04 | 1.000 | inguinal hernia | Medical history | |
| X131620 | 1.59 | 0.007 | 1.63 | 1.000 | 1.83 | 1.000 | 1.83 | 1.000 | 1.63 | 1.000 | 1.47 | 1.000 | diaphragmatic hernia | Medical history | |
| X131630 | 2.28 | 3.91×10^-10^ | 2.26 | 0.086 | 3.10 | 1.27×10^-04^ | 3.10 | 1.26×10^-04^ | 2.26 | 0.086 | 1.98 | 0.021 | other non -infective gastro -enteritis and colitis | Medical history | |
| X131636 | 1.33 | 1.000 | 1.47 | 1.000 | 1.59 | 1.000 | 1.59 | 1.000 | 1.47 | 1.000 | 1.14 | 1.000 | diverticular Medical history of intestine | Medical history | |
| X131638 | 1.39 | 0.934 | 1.11 | 1.000 | 1.40 | 1.000 | 1.40 | 1.000 | 1.11 | 1.000 | 1.52 | 1.000 | irritable bowel syndrome | Medical history | |
| X131640 | 2.41 | 4.53×10^-07^ | 2.58 | 0.155 | 2.60 | 0.528 | 2.60 | 0.526 | 2.58 | 0.155 | 2.25 | 0.029 | other functional intestinal disorders | Medical history | |
| X131646 | 1.39 | 1.000 | 1.81 | 1.000 | 1.61 | 1.000 | 1.61 | 1.000 | 1.81 | 1.000 | 1.10 | 1.000 | other medical historys of anus and rectum | Medical history | |
| X131650 | 0.79 | 1.000 | 0.85 | 1.000 | 0.82 | 1.000 | 0.82 | 1.000 | 0.85 | 1.000 | 0.74 | 1.000 | haemorrhoids and perianal venous thrombosis | Medical history | |
| X131674 | 0.94 | 1.000 | 0.89 | 1.000 | 1.37 | 1.000 | 1.37 | 1.000 | 0.89 | 1.000 | 0.76 | 1.000 | cholelithiasis | Medical history | |
| X131702 | 1.51 | 1.000 | 1.17 | 1.000 | 2.19 | 0.523 | 2.19 | 0.521 | 1.17 | 1.000 | 1.39 | 1.000 | cellulitis | Medical history | |
| X131708 | 1.24 | 1.000 | 1.49 | 1.000 | 0.93 | 1.000 | 0.93 | 1.000 | 1.49 | 1.000 | 1.23 | 1.000 | other local infections of skin and subcutaneous tissue | Medical history | |
| X131720 | 0.78 | 1.000 | 0.57 | 1.000 | 0.85 | 1.000 | 0.85 | 1.000 | 0.57 | 1.000 | 0.86 | 1.000 | atopic dermatitis | Medical history | |
| X131740 | 0.88 | 1.000 | 0.92 | 1.000 | 1.30 | 1.000 | 1.30 | 1.000 | 0.92 | 1.000 | 0.69 | 1.000 | other dermatitis | Medical history | |
| X131742 | 0.87 | 1.000 | 1.32 | 1.000 | 0.67 | 1.000 | 0.67 | 1.000 | 1.32 | 1.000 | 0.72 | 1.000 | psoriasis | Medical history | |
| X131768 | 0.77 | 1.000 | 0.35 | 1.000 | 0.86 | 1.000 | 0.86 | 1.000 | 0.35 | 1.000 | 0.97 | 1.000 | skin changes due to chronic exposure to nonionising radiation | Medical history | |
| X131794 | 0.96 | 1.000 | 1.73 | 1.000 | 0.76 | 1.000 | 0.76 | 1.000 | 1.73 | 1.000 | 0.65 | 1.000 | follicular cysts of skin and subcutaneous tissue | Medical history | |
| X131806 | 0.70 | 1.000 | 0.96 | 1.000 | 1.07 | 1.000 | 1.07 | 1.000 | 0.96 | 1.000 | 0.38 | 1.000 | seborrhoeic keratosis | Medical history | |
| X131836 | 0.78 | 1.000 | 0.41 | 1.000 | 1.27 | 1.000 | 1.27 | 1.000 | 0.41 | 1.000 | 0.78 | 1.000 | other disorders of skin and subcutaneous tissue, not elsewhere classified | Medical history | |
| X131858 | 0.92 | 1.000 | 1.46 | 1.000 | 0.79 | 1.000 | 0.79 | 1.000 | 1.46 | 1.000 | 0.66 | 1.000 | gout | Medical history | |
| X131864 | 1.18 | 1.000 | 1.34 | 1.000 | 1.40 | 1.000 | 1.40 | 1.000 | 1.34 | 1.000 | 1.00 | 1.000 | other arthritis | Medical history | |
| X131872 | 1.52 | 0.686 | 1.76 | 1.000 | 2.35 | 0.077 | 2.35 | 0.077 | 1.76 | 1.000 | 0.98 | 1.000 | gonarthrosis [arthrosis of knee] | Medical history | |
| X131876 | 1.20 | 1.000 | 1.16 | 1.000 | 1.27 | 1.000 | 1.27 | 1.000 | 1.16 | 1.000 | 1.19 | 1.000 | other arthrosis | Medical history | |
| X131878 | 1.20 | 1.000 | 1.29 | 1.000 | 1.44 | 1.000 | 1.44 | 1.000 | 1.29 | 1.000 | 1.05 | 1.000 | acquired deformities of fingers and toes | Medical history | |
| X131884 | 0.86 | 1.000 | 0.50 | 1.000 | 1.40 | 1.000 | 1.40 | 1.000 | 0.50 | 1.000 | 0.82 | 1.000 | internal derangement of knee | Medical history | |
| X131888 | 0.98 | 1.000 | 1.14 | 1.000 | 0.93 | 1.000 | 0.93 | 1.000 | 1.14 | 1.000 | 0.91 | 1.000 | other joint disorders, not elsewhere classified | Medical history | |
| X131916 | 1.43 | 1.000 | 1.16 | 1.000 | 1.66 | 1.000 | 1.66 | 1.000 | 1.16 | 1.000 | 1.49 | 1.000 | spondylosis | Medical history | |
| X131924 | 0.84 | 1.000 | 1.13 | 1.000 | 0.77 | 1.000 | 0.77 | 1.000 | 1.13 | 1.000 | 0.71 | 1.000 | other intervertebral disk disorders | Medical history | |
| X131928 | 1.19 | 1.000 | 1.20 | 1.000 | 1.35 | 1.000 | 1.35 | 1.000 | 1.20 | 1.000 | 1.11 | 1.000 | dorsalgia | Medical history | |
| X131938 | 0.93 | 1.000 | 0.72 | 1.000 | 1.15 | 1.000 | 1.15 | 1.000 | 0.72 | 1.000 | 0.93 | 1.000 | synovitis and tenosynovitis | Medical history | |
| X131946 | 0.74 | 1.000 | 1.30 | 1.000 | 0.59 | 1.000 | 0.59 | 1.000 | 1.30 | 1.000 | 0.48 | 1.000 | soft tissue disorders related to use, overuse and pressure | Medical history | |
| X131950 | 0.77 | 1.000 | 0.48 | 1.000 | 0.71 | 1.000 | 0.71 | 1.000 | 0.48 | 1.000 | 0.97 | 1.000 | fibroblastic disorders | Medical history | |
| X131954 | 0.82 | 1.000 | 0.41 | 1.000 | 1.72 | 1.000 | 1.72 | 1.000 | 0.41 | 1.000 | 0.62 | 1.000 | shoulder lesions | Medical history | |
| X131958 | 0.77 | 1.000 | 0.88 | 1.000 | 0.66 | 1.000 | 0.66 | 1.000 | 0.88 | 1.000 | 0.77 | 1.000 | other enthesopathies | Medical history | |
| X131960 | 1.14 | 1.000 | 1.00 | 1.000 | 1.15 | 1.000 | 1.15 | 1.000 | 1.00 | 1.000 | 1.21 | 1.000 | other soft tissue disorders, not elsewhere classified | Medical history | |
| X131964 | 1.73 | 0.156 | 2.30 | 0.632 | 1.87 | 1.000 | 1.87 | 1.000 | 2.30 | 0.630 | 1.39 | 1.000 | osteoporosis without pathological fracture | Medical history | |
| X132054 | 1.29 | 1.000 | 1.18 | 1.000 | 1.46 | 1.000 | 1.46 | 1.000 | 1.18 | 1.000 | 1.27 | 1.000 | cystitis | Medical history | |
| X132070 | 1.38 | 0.877 | 1.59 | 1.000 | 1.41 | 1.000 | 1.41 | 1.000 | 1.59 | 1.000 | 1.26 | 1.000 | other disorders of urinary system | Medical history | |
| X132072 | 0.97 | 1.000 | 0.96 | 1.000 | 0.70 | 1.000 | 0.70 | 1.000 | 0.96 | 1.000 | 1.09 | 1.000 | hyperplasia of prostate | Medical history | |
| X132096 | 0.71 | 1.000 | 0.80 | 1.000 | 0.83 | 1.000 | 0.83 | 1.000 | 0.80 | 1.000 | 0.61 | 1.000 | benign mammary dysplasia | Medical history | |
| X132102 | 0.93 | 1.000 | 1.17 | 1.000 | 0.85 | 1.000 | 0.85 | 1.000 | 1.17 | 1.000 | 0.84 | 1.000 | unspecified lump in breast | Medical history | |
| X132104 | 0.64 | 1.000 | 0.83 | 1.000 | 0.59 | 1.000 | 0.59 | 1.000 | 0.83 | 1.000 | 0.57 | 1.000 | other disorders of breast | Medical history | |
| X132124 | 1.27 | 1.000 | 1.61 | 1.000 | 1.44 | 1.000 | 1.44 | 1.000 | 1.61 | 1.000 | 1.02 | 1.000 | female genital prolapse | Medical history | |
| X132128 | 1.40 | 1.000 | 1.46 | 1.000 | 1.63 | 1.000 | 1.63 | 1.000 | 1.46 | 1.000 | 1.28 | 1.000 | noninflammatory disorders of ovary, fallopian tube and broad ligament | Medical history | |
| X132130 | 0.82 | 1.000 | 0.61 | 1.000 | 0.59 | 1.000 | 0.59 | 1.000 | 0.61 | 1.000 | 1.03 | 1.000 | polyp of female genital tract | Medical history | |
| X132140 | 0.97 | 1.000 | 0.88 | 1.000 | 0.65 | 1.000 | 0.65 | 1.000 | 0.88 | 1.000 | 1.15 | 1.000 | other noninflammatory disorders of vagina | Medical history | |
| X132146 | 0.83 | 1.000 | 0.76 | 1.000 | 0.87 | 1.000 | 0.87 | 1.000 | 0.76 | 1.000 | 0.85 | 1.000 | excessive, frequent and irregular menstruation | Medical history | |
| X132148 | 0.77 | 1.000 | 0.74 | 1.000 | 0.68 | 1.000 | 0.68 | 1.000 | 0.74 | 1.000 | 0.83 | 1.000 | other abnormal uterine and vaginal bleeding | Medical history | |
| X132150 | 1.20 | 1.000 | 1.54 | 1.000 | 1.15 | 1.000 | 1.15 | 1.000 | 1.54 | 1.000 | 1.05 | 1.000 | pain and other conditions associated with female genital organs and menstrual cycle | Medical history | |
| X132152 | 1.08 | 1.000 | 0.96 | 1.000 | 0.92 | 1.000 | 0.92 | 1.000 | 0.96 | 1.000 | 1.23 | 1.000 | menopausal and other perimenopausal disorders | Medical history | |
| X132276 | 0.86 | 1.000 | 0.95 | 1.000 | 0.92 | 1.000 | 0.92 | 1.000 | 0.95 | 1.000 | 0.80 | 1.000 | single spontaneous delivery | Medical history | |
| Stroke | 2.33 | 6.47×10^-06^ | 2.37 | 0.633 | 3.35 | 0.004 | 3.35 | 0.004 | 2.37 | 0.631 | 1.89 | 1.000 | stroke | Medical history | |
| TBI | 1.05 | 1.000 | 0.94 | 1.000 | 1.28 | 1.000 | 1.28 | 1.000 | 0.94 | 1.000 | 1.03 | 1.000 | Traumatic brain injury | Medical history | |
| Vision_impairment | 0.89 | 1.000 | 0.92 | 1.000 | 0.98 | 1.000 | 0.98 | 1.000 | 0.92 | 1.000 | 0.83 | 1.000 | Vision_impairment | Medical history | |
| Dementia | 7.52 | 0.147 | - | - | 11.82 | 1.000 | 11.82 | 1.000 | - | - | 10.19 | 0.312 | dementia | Mental health | |
| Intellectual disability | 35.15 | 2.96×10^-07^ | 36.49 | 0.091 | - | - | - | - | 36.49 | 0.090 | 47.62 | 4.00×10^-05^ | Intellectual disability | Mental health | |
| Others personality disorders | 3.76 | 1.000 | - | - | 11.27 | 1.000 | 11.27 | 1.000 | - | - | 3.50 | 1.000 | others disorders of adult personality and behaviour | Mental health | |
| Others behavioural syndromes | 22.29 | 0.671 | - | - | 100.84 | 0.001 | 100.84 | 9.25×10^-04^ | - | - | - | - | others behavioural syndromes associated with physiological disturbances and physical factors | Mental health | |
| Others substance abuse | 5.98 | 1.000 | - | - | - | - | - | - | - | - | 8.59 | 1.000 | others mental and behavioural disorders due to psychoactive substance use | Mental health | |
| Others mood disorders | 5.09 | 0.001 | 9.95 | 0.002 | - | - | - | - | 9.95 | 0.002 | 4.75 | 0.548 | others mood [affective] disorders | Mental health | |
| Other organic mental disorders | 20.63 | 1.40×10^-72^ | 27.03 | 0.000 | 11.14 | 0.010 | 11.14 | 0.010 | 27.03 | 7.94×10^-29^ | 20.03 | 1.42×10^-40^ | other organic, including symptomatic, mental disorders | Mental health | |
| Other mental disorders | 12.02 | 1.58×10^-16^ | 21.32 | 3.89×10^-09^ | - | - | - | - | 71.29 | 9.77×10^-19^ | 28.67 | 1.20×10^-18^ | Other mental and behavioural disorders | Mental health | |
| Psychological development disorders | 23.73 | 6.93×10^-27^ | 31.90 | 8.89×10^-12^ | 27.69 | 3.50×10^-06^ | 27.69 | 3.49×10^-06^ | 31.90 | 8.86×10^-12^ | 17.73 | 5.64×10^-08^ | Disorders of psychological development | Mental health | |
| X130854 | 5.80 | 1.11×10^-33^ | 5.12 | 9.11×10^-06^ | 9.51 | 7.01×10^-14^ | 9.51 | 6.99×10^-14^ | 5.12 | 9.08×10^-06^ | 5.08 | 1.79×10^-14^ | Mental and behavioural disorders due to use of alcohol | Mental health | |
| X130856 | 15.93 | 2.78×10^-07^ | - | - | 64.88 | 5.57×10^-10^ | 64.88 | 5.55×10^-10^ | - | - | 10.36 | 0.293 | Mental and behavioural disorders due to use of opioids | Mental health | |
| X130858 | 20.05 | 6.80×10^-07^ | - | - | 32.33 | 0.179 | 32.33 | 0.179 | - | - | 26.08 | 5.52×10^-06^ | Mental and behavioural disorders due to use of cannabinoids | Mental health | |
| X130860 | 2.13 | 1.000 | - | - | 11.73 | 1.000 | 11.73 | 1.000 | - | - | - | - | Mental and behavioural disorders due to use of sedatives or hypnotics | Mental health | |
| X130868 | 1.69 | 5.87×10^-04^ | 1.52 | 1.000 | 1.54 | 1.000 | 1.54 | 1.000 | 1.52 | 1.000 | 1.83 | 0.013 | Mental and behavioural disorders due to use of tobacco | Mental health | |
| X130872 | 15.62 | 2.36×10^-10^ | 19.15 | 0.010 | 36.56 | 3.51×10^-07^ | 36.56 | 3.50×10^-07^ | 19.15 | 0.010 | 7.57 | 1.000 | Mental and behavioural disorders due to multiple drug use and use of other psychoactive substances | Mental health | |
| X130890 | 33.14 | 2.50×10^-50^ | 56.06 | 8.39×10^-20^ | 30.74 | 4.75×10^-04^ | 30.74 | 4.73×10^-04^ | 56.06 | 8.36×10^-20^ | 27.59 | 4.80×10^-27^ | Manic episode | Mental health | |
| X130892 | 32.91 | <2×10^-16^ | 40.50 | 4.51×10^-81^ | 36.74 | 1.12×10^-41^ | 36.74 | 1.12×10^-41^ | 40.50 | 4.50×10^-81^ | 29.41 | <2×10^-16^ | Bipolar affective disorder | Mental health | |
| X130894 | 4.95 | <2×10^-16^ | 5.29 | 2.14×10^-42^ | 5.41 | 1.54×10^-34^ | 5.41 | 1.54×10^-34^ | 5.29 | 2.13×10^-42^ | 4.61 | 1.19×10^-67^ | Depressive episode | Mental health | |
| X130896 | 8.85 | 3.19×10^-54^ | 9.45 | 7.13×10^-15^ | 9.92 | 1.77×10^-11^ | 9.92 | 1.77×10^-11^ | 9.45 | 7.10×10^-15^ | 8.25 | 1.13×10^-26^ | Recurrent depressive disorder | Mental health | |
| X130898 | 5.32 | 1.91×10^-04^ | 7.28 | 0.172 | 7.08 | 1.000 | 7.08 | 1.000 | 7.28 | 0.171 | 4.01 | 1.000 | Persistent mood [affective] disorders | Mental health | |
| X130904 | 2.33 | 1.000 | 2.85 | 1.000 | 2.35 | 1.000 | 2.35 | 1.000 | 2.85 | 1.000 | 2.03 | 1.000 | Phobic anxiety disorders | Mental health | |
| X130906 | 3.55 | 1.55×10^-45^ | 4.18 | 1.65×10^-16^ | 2.95 | 1.07×10^-04^ | 2.95 | 1.07×10^-04^ | 4.18 | 1.64×10^-16^ | 3.47 | 3.76×10^-23^ | Other anxiety disorders | Mental health | |
| X130908 | 11.05 | 2.71×10^-20^ | 12.60 | 4.82×10^-06^ | 16.23 | 9.68×10^-06^ | 16.23 | 9.64×10^-06^ | 12.60 | 4.81×10^-06^ | 8.86 | 2.62×10^-07^ | Obsessiv×10-compulsive disorder | Mental health | |
| X130910 | 1.78 | 0.037 | 1.67 | 1.000 | 1.12 | 1.000 | 1.12 | 1.000 | 1.67 | 1.000 | 2.08 | 0.034 | Reaction to severe stress, and adjustment disorders | Mental health | |
| X130912 | 18.48 | 4.07×10^-12^ | 48.05 | 2.67×10^-15^ | - | - | - | - | 48.05 | 2.66×10^-15^ | 10.36 | 0.261 | Dissociative [conversion] disorders | Mental health | |
| X130914 | 2.47 | 0.558 | 3.63 | 1.000 | 3.17 | 1.000 | 3.17 | 1.000 | 3.63 | 1.000 | 1.57 | 1.000 | Somatoform disorders | Mental health | |
| X130916 | 14.58 | 3.06×10^-52^ | 18.70 | 2.44×10^-19^ | 13.92 | 6.82×10^-08^ | 13.92 | 6.80×10^-08^ | 18.70 | 2.44×10^-19^ | 13.08 | 2.36×10^-24^ | Other neurotic disorders | Mental health | |
| X130918 | 5.19 | 7.71×10^-05^ | 6.45 | 0.421 | 7.65 | 0.138 | 7.65 | 0.137 | 6.45 | 0.419 | 3.70 | 1.000 | Eating disorders | Mental health | |
| X130920 | 2.03 | 1.000 | - | - | - | - | - | - | - | - | 3.77 | 1.000 | Nonorganic sleep disorders | Mental health | |
| X130922 | 1.97 | 1.000 | 1.04 | 1.000 | 1.31 | 1.000 | 1.31 | 1.000 | 1.04 | 1.000 | 2.72 | 1.000 | Sexual dysfunction, not caused by organic disorder or disease | Mental health | |
| X130924 | 4.26 | 2.49×10^-05^ | 7.54 | 3.42×10^-04^ | 3.54 | 1.000 | 3.54 | 1.000 | 7.54 | 3.41×10^-04^ | 3.10 | 1.000 | Mental and behavioural disorders associated with the puerperium, not elsewhere classified | Mental health | |
| X130932 | 20.10 | 4.22×10^-32^ | 18.80 | 1.79×10^-06^ | 23.63 | 1.80×10^-05^ | 23.63 | 1.79×10^-05^ | 18.80 | 1.79×10^-06^ | 19.67 | 2.48×10^-18^ | Specific personality disorders | Mental health | |
| X130944 | 1.08 | 1.000 | - | - | - | - | - | - | - | - | 2.38 | 1.000 | Psychological and behavioural disorders associated with sexual development and orientation | Mental health | |
| X1920 | 1.86 | 4.65×10^-25^ | 1.97 | 1.78×10^-07^ | 1.61 | 0.018 | 1.61 | 0.018 | 1.97 | 1.78×10^-07^ | 1.92 | 2.46×10^-13^ | Mood swings | Mental health | |
| X1930 | 1.53 | 1.70×10^-11^ | 1.51 | 0.040 | 1.35 | 1.000 | 1.35 | 1.000 | 1.51 | 0.040 | 1.63 | 3.54×10^-07^ | Miserableness | Mental health | |
| X1940 | 1.21 | 0.448 | 1.08 | 1.000 | 1.37 | 1.000 | 1.37 | 1.000 | 1.08 | 1.000 | 1.22 | 1.000 | Irritability | Mental health | |
| X1950 | 1.39 | 7.48×10^-06^ | 1.54 | 0.038 | 1.07 | 1.000 | 1.07 | 1.000 | 1.54 | 0.038 | 1.47 | 0.001 | Sensitivity / hurt feelings | Mental health | |
| X1960 | 1.61 | 4.53×10^-15^ | 1.64 | 0.001 | 1.53 | 0.098 | 1.53 | 0.098 | 1.64 | 0.001 | 1.64 | 1.28×10^-07^ | Fed-up feelings | Mental health | |
| X1970 | 2.46 | 1.57×10^-53^ | 2.69 | 2.32×10^-17^ | 2.09 | 1.45×10^-06^ | 2.09 | 1.44×10^-06^ | 2.69 | 2.32×10^-17^ | 2.50 | 2.98×10^-28^ | Nervous feelings | Mental health | |
| X1980 | 1.79 | 1.01×10^-18^ | 1.94 | 6.21×10^-06^ | 1.65 | 0.033 | 1.65 | 0.033 | 1.94 | 6.18×10^-06^ | 1.79 | 1.05×10^-08^ | Worrier / anxious feelings | Mental health | |
| X1990 | 2.40 | 8.67×10^-47^ | 2.65 | 1.59×10^-15^ | 2.21 | 7.61×10^-07^ | 2.21 | 7.58×10^-07^ | 2.65 | 1.58×10^-15^ | 2.35 | 5.81×10^-23^ | Tense / 'highly strung | Mental health | |
| X2000 | 1.32 | 2.40×10^-04^ | 1.23 | 1.000 | 1.17 | 1.000 | 1.17 | 1.000 | 1.23 | 1.000 | 1.45 | 0.001 | Worry too long after embarrassment | Mental health | |
| X2010 | 2.69 | 4.51×10^-66^ | 2.78 | 2.03×10^-18^ | 2.21 | 6.68×10^-08^ | 2.21 | 6.66×10^-08^ | 2.78 | 2.02×10^-18^ | 2.86 | 2.57×10^-38^ | Suffer from 'nerves' | Mental health | |
| X2020 | 2.53 | 9.57×10^-53^ | 3.12 | 5.12×10^-22^ | 2.31 | 3.16×10^-08^ | 2.31 | 3.15×10^-08^ | 3.12 | 5.10×10^-22^ | 2.34 | 3.69×10^-22^ | Loneliness, isolation | Mental health | |
| X2030 | 1.54 | 5.91×10^-11^ | 1.74 | 2.13×10^-04^ | 1.26 | 1.000 | 1.26 | 1.000 | 1.74 | 2.13×10^-04^ | 1.56 | 1.48×10^-05^ | Guilty feelings | Mental health | |
| X2110 | 1.42 | 1.04×10^-06^ | 1.21 | 1.000 | 1.53 | 0.251 | 1.53 | 0.250 | 1.21 | 1.000 | 1.50 | 2.46×10^-04^ | Able to confide | Mental health | |
| Depression | 2.48 | 4.97×10^-55^ | 2.68 | 3.53×10^-17^ | 2.50 | 1.41×10^-11^ | 2.50 | 1.41×10^-11^ | 2.68 | 3.52×10^-17^ | 2.37 | 4.24×10^-25^ | frequency of depressed mood in last 2 weeks | Mental health | |
| X6145 | 1.53 | 1.56×10^-11^ | 1.65 | 0.001 | 1.53 | 0.108 | 1.53 | 0.108 | 1.65 | 0.001 | 1.46 | 3.74×10^-04^ | illness injury bereavement stress in last 2 years | Mental health | |
| X2090 | 3.46 | 5.20×10^-95^ | 4.64 | 1.77×10^-36^ | 2.69 | 1.36×10^-13^ | - | - | - | - | - | - | seen doctor gp for nerves anxiety tension or depression | Mental health | |
| X2100 | 6.64 | <2×10^-16^ | 7.52 | 7.02×10^-76^ | 6.40 | 1.12×10^-49^ | 6.40 | 1.12×10^-49^ | 7.52 | 7.00×10^-76^ | 6.32 | <2×10^-16^ | seen a psychiatrist for nerves anxiety tension or depression | Mental health | |
| X2070 | 2.27 | 2.09×10^-45^ | 2.09 | 4.67×10^-09^ | 2.22 | 1.32×10^-08^ | 2.22 | 1.32×10^-08^ | 2.09 | 4.66×10^-09^ | 2.39 | 4.99×10^-26^ | frequency of tenseness restlessness in last 2 week | Mental health | |
| X2080 | 1.69 | 2.35×10^-16^ | 1.80 | 6.71×10^-05^ | 1.97 | 3.63×10^-05^ | 1.97 | 3.61×10^-05^ | 1.80 | 6.69×10^-05^ | 1.53 | 6.41×10^-05^ | frequency of tiredness lethargy in last 2 weeks | Mental health | |
| X2060 | 2.44 | 2.47×10^-52^ | 2.99 | 1.27×10^-21^ | 2.48 | 7.82×10^-11^ | 2.48 | 7.80×10^-11^ | 2.99 | 1.26×10^-21^ | 2.18 | 7.51×10^-20^ | frequency of unenthusiasm disinterest in last 2 weeks | | Mental health |
| Arm_fat_percentage | 1.30 | 0.261 | 1.18 | 1.000 | 1.11 | 1.000 | 1.11 | 1.000 | 1.18 | 1.000 | 1.47 | 0.184 | Arm fat percentage | Physical measures | |
| Arm_fat_percentage | 1.80 | 3.71×10^-06^ | 1.92 | 0.343 | 1.30 | 1.000 | 1.30 | 1.000 | 1.92 | 0.341 | 2.00 | 0.001 | Arm fat percentage | Physical measures | |
| X4079 | 0.83 | 1.000 | 0.86 | 1.000 | 0.65 | 1.000 | 0.65 | 1.000 | 0.86 | 1.000 | 0.90 | 1.000 | Diastolic blood pressure | Physical measures | |
| X4079 | 0.97 | 1.000 | 1.11 | 1.000 | 0.77 | 1.000 | 0.77 | 1.000 | 1.11 | 1.000 | 1.00 | 1.000 | Diastolic blood pressure | Physical measures | |
| Hand_grip_strength | 0.62 | 1.30×10^-07^ | 0.57 | 0.131 | 0.65 | 1.000 | 0.65 | 1.000 | 0.57 | 0.131 | 0.63 | 0.003 | Hand grip strength | Physical measures | |
| Hand_grip_strength | 0.35 | 3.53×10^-20^ | 0.35 | 1.83×10^-04^ | 0.37 | 0.005 | 0.37 | 0.005 | 0.35 | 1.83×10^-04^ | 0.35 | 1.20×10^-10^ | Hand grip strength | Physical measures | |
| Leg_fat_percentage | 1.23 | 1.000 | 1.40 | 1.000 | 0.86 | 1.000 | 0.86 | 1.000 | 1.40 | 1.000 | 1.32 | 1.000 | Leg fat percentage | Physical measures | |
| Leg_fat_percentage | 1.66 | 0.010 | 2.00 | 0.860 | 0.85 | 1.000 | 0.85 | 1.000 | 2.00 | 0.857 | 1.97 | 0.018 | Leg fat percentage | Physical measures | |
| Lung_function | 0.61 | 3.52×10^-11^ | 0.71 | 1.000 | 0.56 | 0.027 | 0.56 | 0.027 | 0.71 | 1.000 | 0.58 | 1.58×10^-06^ | Lung_function | Physical measures | |
| Lung_function | 0.40 | 5.05×10^-26^ | 0.43 | 2.59×10^-05^ | 0.45 | 0.001 | 0.45 | 0.001 | 0.43 | 2.58×10^-05^ | 0.37 | 4.15×10^-15^ | Lung_function | Physical measures | |
| X23099 | 1.22 | 1.000 | 1.15 | 1.000 | 1.02 | 1.000 | 1.02 | 1.000 | 1.15 | 1.000 | 1.37 | 1.000 | Body fat percentage | Physical measures | |
| X23099 | 1.55 | 0.002 | 1.63 | 1.000 | 1.04 | 1.000 | 1.04 | 1.000 | 1.63 | 1.000 | 1.79 | 0.008 | Body fat percentage | Physical measures | |
| X23127 | 1.14 | 1.000 | 0.97 | 1.000 | 0.97 | 1.000 | 0.97 | 1.000 | 0.97 | 1.000 | 1.31 | 1.000 | Trunk fat percentage | Physical measures | |
| X23127 | 1.43 | 0.001 | 1.51 | 1.000 | 1.08 | 1.000 | 1.08 | 1.000 | 1.51 | 1.000 | 1.58 | 0.016 | Trunk fat percentage | Physical measures | |
| X23105 | 1.12 | 1.000 | 1.20 | 1.000 | 1.17 | 1.000 | 1.17 | 1.000 | 1.20 | 1.000 | 1.06 | 1.000 | Basal metabolic rate | Physical measures | |
| X23105 | 1.08 | 1.000 | 1.38 | 1.000 | 0.89 | 1.000 | 0.89 | 1.000 | 1.38 | 1.000 | 1.03 | 1.000 | Basal metabolic rate | Physical measures | |
| X4080 | 0.84 | 1.000 | 0.85 | 1.000 | 0.74 | 1.000 | 0.74 | 1.000 | 0.85 | 1.000 | 0.88 | 1.000 | Systolic blood pressure | Physical measures | |
| X4080 | 0.89 | 1.000 | 0.96 | 1.000 | 0.57 | 0.159 | 0.57 | 0.158 | 0.96 | 1.000 | 1.02 | 1.000 | Systolic blood pressure | Physical measures | |
| X6138 | 1.50 | 1.01×10^-09^ | 1.29 | 1.000 | 1.53 | 0.259 | 1.53 | 0.258 | 1.29 | 1.000 | 1.62 | 1.20×10^-06^ | Lower education | SES | |
| X6142 | 2.07 | 2.04×10^-07^ | 3.14 | 1.90×10^-06^ | 2.13 | 0.922 | 2.13 | 0.919 | 3.14 | 1.89×10^-06^ | 1.56 | 1.000 | Unemployed | SES | |
| X738 | 0.44 | 3.41×10^-29^ | 0.46 | 6.11×10^-06^ | 0.45 | 1.62×10^-05^ | 0.45 | 1.61×10^-05^ | 0.46 | 6.09×10^-06^ | 0.42 | 8.01×10^-16^ | Average total household income before tax | SES | |
| X738 | 0.28 | 5.09×10^-48^ | 0.35 | 2.31×10^-09^ | 0.22 | 1.50×10^-11^ | 0.22 | 1.49×10^-11^ | 0.35 | 2.30×10^-09^ | 0.28 | 3.27×10^-25^ | Average total household income before tax | SES | |
| X738 | 0.19 | 1.62×10^-48^ | 0.24 | 1.85×10^-10^ | 0.16 | 1.67×10^-10^ | 0.16 | 1.66×10^-10^ | 0.24 | 1.84×10^-10^ | 0.18 | 1.60×10^-25^ | Average total household income before tax | SES | |
| X738 | 0.14 | 9.80×10^-16^ | 0.27 | 0.033 | 0.12 | 0.011 | 0.12 | 0.011 | 0.27 | 0.032 | 0.09 | 9.00×10^-08^ | Average total household income before tax | SES | |
| X22189 | 1.28 | 0.890 | 1.25 | 1.000 | 1.13 | 1.000 | 1.13 | 1.000 | 1.25 | 1.000 | 1.36 | 1.000 | Townsend deprivation index at recruitment | SES | |
| X22189 | 2.65 | 5.38×10^-39^ | 2.51 | 4.47×10^-09^ | 2.76 | 3.19×10^-09^ | 2.76 | 3.18×10^-09^ | 2.51 | 4.46×10^-09^ | 2.69 | 4.74×10^-19^ | Townsend deprivation index at recruitment | SES | |
|  | | | | | | | | | | | | | | | |

| **Supplementary Table 10. Risk of incident late onset schizophrenia according to profiles of 6 risk domains.** | | | | | | | | |
| --- | --- | --- | --- | --- | --- | --- | --- | --- |
| **Subgroup** | **Model 1** | | | | **Model 2** | | | |
|  | **HR** | **5% CI** | **95% CI** | ***P*-value** | **HR** | **5% CI** | **95% CI** | ***P*-value** |
| **Medical history** | | | | | | | | |
| Favorable | 1.00 |  |  | Reference | 1.00 |  |  | Reference |
| Intermediate | 1.15 | 0.96 | 1.37 | 0.126 | 0.97 | 0.81 | 1.16 | 0.725 |
| Unfavorable | 3.27 | 2.86 | 3.74 | 4.43×10^-67^ | 1.91 | 1.67 | 2.20 | 5.46×10^-20^ |
| *P* for trend |  |  |  | 3.56×10^-72^ |  |  |  | 1.24×10^-23^ |
| **Local environment** | | | | | | | | |
| Favorable | 1.00 |  |  | Reference | 1.00 |  |  | Reference |
| Intermediate | 1.49 | 1.28 | 1.73 | 1.93×10^-07^ | 1.23 | 1.06 | 1.43 | 0.007 |
| Unfavorable | 2.04 | 1.76 | 2.35 | 2.22×10^-22^ | 1.38 | 1.19 | 1.60 | 1.61×10^-05^ |
| *P* for trend |  |  |  | 6.37×10^-23^ |  |  |  | 2.80×10^-05^ |
| **Lifestyles** | | | | | | | | |
| Favorable | 1.00 |  |  | Reference | 1.00 |  |  | Reference |
| Intermediate | 1.51 | 1.26 | 1.80 | 4.88×10^-06^ | 1.13 | 0.95 | 1.35 | 0.169 |
| Unfavorable | 3.69 | 3.16 | 4.31 | 2.16×10^-61^ | 1.82 | 1.55 | 2.15 | 6.66×10^-13^ |
| *P* for trend |  |  |  | 1.27×10^-73^ |  |  |  | 2.12×10^-17^ |
| **Mental health** | | | | | | | | |
| Favorable | 1.00 |  |  | Reference | 1.00 |  |  | Reference |
| Intermediate | 1.31 | 1.07 | 1.61 | 0.009 | 1.21 | 0.99 | 1.48 | 0.069 |
| Unfavorable | 5.46 | 4.62 | 6.46 | 3.36×10^-88^ | 3.84 | 3.24 | 4.56 | 3.07×10^-54^ |
| *P* for trend |  |  |  | <2.00×10^-16^ |  |  |  | 3.43×10^-74^ |
| **Physical measures** | | | | | | | | |
| Favorable | 1.00 |  |  | Reference | 1.00 |  |  | Reference |
| Intermediate | 2.04 | 1.74 | 2.39 | 3.39×10^-18^ | 1.33 | 1.13 | 1.57 | 5.19×10^-04^ |
| Unfavorable | 3.96 | 3.24 | 4.85 | 1.09×10^-40^ | 1.77 | 1.44 | 2.17 | 6.28×10^-08^ |
| *P* for trend |  |  |  | 1.01×10^-40^ |  |  |  | 2.38×10^-08^ |
| **SES** | | | | | | | | |
| Favorable | 1.00 |  |  | Reference | 1.00 |  |  | Reference |
| Intermediate | 1.77 | 1.49 | 2.10 | 9.41×10^-11^ | 1.31 | 1.10 | 1.56 | 0.003 |
| Unfavorable | 4.36 | 3.73 | 5.09 | 2.37×10^-77^ | 2.09 | 1.77 | 2.46 | 3.65×10^-18^ |
| *P* for trend |  |  |  | 4.65×10^-88^ |  |  |  | 1.92×10^-22^ |
| Model 1 adjusted for age, sex, assessment center and genetic risk. Model 2 further added all six risk domains which were mutually adjusted. SES, socioeconomic status; HR, hazard ratio; CI, confidence interval. | | | | | | | | |

| **Supplementary Table 11. Risk of incident late onset schizophrenia according to profiles of 6 risk domains stratified by age** | | | | | | | | |
| --- | --- | --- | --- | --- | --- | --- | --- | --- |
| **Subgroup** | **Aged <65 years old** | | | | **Aged ≥65 years old** | | | |
|  | **HR** | **5% CI** | **95% CI** | ***P*-value** | **HR** | **5% CI** | **95% CI** | ***P*-value** |
| **Medical history** | | | | | | | | |
| Favorable | 1.00 |  |  | Reference | 1.00 |  |  | Reference |
| Intermediate | 0.98 | 0.80 | 1.19 | 0.822 | 0.91 | 0.61 | 1.34 | 0.625 |
| Unfavorable | 1.92 | 1.65 | 2.24 | 1.33×10^-16^ | 1.81 | 1.35 | 2.43 | 8.26×10^-05^ |
| *P* for trend |  |  |  | 5.02×10^-18^ |  |  |  | 8.22×10^-06^ |
| **Local environment** | | | | | | | | |
| Favorable | 1.00 |  |  | Reference | 1.00 |  |  | Reference |
| Intermediate | 1.17 | 0.98 | 1.40 | 0.075 | 1.39 | 1.04 | 1.86 | 0.026 |
| Unfavorable | 1.28 | 1.08 | 1.52 | 0.004 | 1.70 | 1.27 | 2.26 | 2.90×10^-04^ |
| *P* for trend |  |  |  | 0.007 |  |  |  | 2.74×10^-04^ |
| **Lifestyles** | | | | | | | | |
| Favorable | 1.00 |  |  | Reference | 1.00 |  |  | Reference |
| Intermediate | 1.12 | 0.91 | 1.37 | 0.274 | 1.16 | 0.81 | 1.67 | 0.425 |
| Unfavorable | 1.84 | 1.53 | 2.20 | 5.08×10^-11^ | 1.74 | 1.25 | 2.41 | 8.90×10^-04^ |
| *P* for trend |  |  |  | 2.40×10^-14^ |  |  |  | 1.11×10^-04^ |
| **Mental health** | | | | | | | | |
| Favorable | 1.00 |  |  | Reference | 1.00 |  |  | Reference |
| Intermediate | 1.07 | 0.84 | 1.38 | 0.571 | 1.54 | 1.08 | 2.20 | 0.017 |
| Unfavorable | 3.86 | 3.16 | 4.72 | 5.08×10^-40^ | 3.79 | 2.76 | 5.21 | 1.67×10^-16^ |
| *P* for trend |  |  |  | 3.91×10^-50^ |  |  |  | 2.37×10^-18^ |
| **Physical measures** | | | | | | | | |
| Favorable | 1.00 |  |  | Reference | 1.00 |  |  | Reference |
| Intermediate | 1.37 | 1.14 | 1.65 | 0.001 | 1.21 | 0.87 | 1.68 | 0.255 |
| Unfavorable | 1.73 | 1.37 | 2.20 | 6.21×10^-06^ | 1.90 | 1.21 | 2.98 | 0.005 |
| *P* for trend |  |  |  | 2.88×10^-06^ |  |  |  | 0.006 |
| **SES** | | | | | | | | |
| Favorable | 1.00 |  |  | Reference | 1.00 |  |  | Reference |
| Intermediate | 1.29 | 1.06 | 1.57 | 0.011 | 1.15 | 0.78 | 1.70 | 0.488 |
| Unfavorable | 2.27 | 1.89 | 2.72 | 1.31×10^-18^ | 1.36 | 0.95 | 1.96 | 0.094 |
| *P* for trend |  |  |  | 3.74×10^-22^ |  |  |  | 0.041 |
| Model adjusted for sex, assessment center, genetic risk and all six risk domains. SES, socioeconomic status; HR, hazard ratio; CI, confidence interval. | | | | | | | | |

| **Supplementary Table 12. Risk of incident late onset schizophrenia according to profiles of 6 risk domains stratified by sex** | | | | | | | | |
| --- | --- | --- | --- | --- | --- | --- | --- | --- |
| **Subgroup** | **Female** | | | | **Male** | | | |
|  | **HR** | **5% CI** | **95% CI** | ***P*-value** | **HR** | **5% CI** | **95% CI** | ***P*-value** |
| **Medical history** | | | | | | | | |
| Favorable | 1.00 |  |  | Reference | 1.00 |  |  | Reference |
| Intermediate | 0.79 | 0.62 | 1.01 | 0.065 | 1.22 | 0.94 | 1.57 | 0.131 |
| Unfavorable | 1.80 | 1.50 | 2.16 | 3.43×10^-10^ | 2.08 | 1.69 | 2.57 | 7.22×10^-12^ |
| *P* for trend |  |  |  | 2.53×10^-11^ |  |  |  | 5.52×10^-13^ |
| **Local environment** | | | | | | | | |
| Favorable | 1.00 |  |  | Reference | 1.00 |  |  | Reference |
| Intermediate | 1.17 | 0.95 | 1.44 | 0.138 | 1.30 | 1.04 | 1.63 | 0.019 |
| Unfavorable | 1.42 | 1.16 | 1.72 | 5.25×10^-04^ | 1.34 | 1.07 | 1.66 | 0.010 |
| *P* for trend |  |  |  | 4.61×10^-04^ |  |  |  | 0.017 |
| **Lifestyles** | | | | | | | | |
| Favorable | 1.00 |  |  | Reference | 1.00 |  |  | Reference |
| Intermediate | 1.04 | 0.81 | 1.32 | 0.770 | 1.25 | 0.96 | 1.61 | 0.096 |
| Unfavorable | 1.75 | 1.41 | 2.17 | 3.57×10^-07^ | 1.89 | 1.49 | 2.39 | 1.14×10^-07^ |
| *P* for trend |  |  |  | 1.14×10^-09^ |  |  |  | 2.63×10^-09^ |
| **Mental health** | | | | | | | | |
| Favorable | 1.00 |  |  | Reference | 1.00 |  |  | Reference |
| Intermediate | 1.27 | 0.96 | 1.68 | 0.088 | 1.15 | 0.85 | 1.55 | 0.364 |
| Unfavorable | 3.77 | 3.00 | 4.74 | 8.09×10^-30^ | 3.94 | 3.07 | 5.06 | 4.91×10^-27^ |
| *P* for trend |  |  |  | 4.98×10^-35^ |  |  |  | 6.19×10^-34^ |
| **Physical measures** | | | | | | | | |
| Favorable | 1.00 |  |  | Reference | 1.00 |  |  | Reference |
| Intermediate | 1.16 | 0.66 | 2.04 | 0.611 | 1.28 | 1.07 | 1.54 | 0.006 |
| Unfavorable | 1.49 | 0.85 | 2.62 | 0.167 | 1.96 | 1.47 | 2.61 | 4.72×10^-06^ |
| *P* for trend |  |  |  | 0.004 |  |  |  | 3.36×10^-06^ |
| **SES** | | | | | | | | |
| Favorable | 1.00 |  |  | Reference | 1.00 |  |  | Reference |
| Intermediate | 1.24 | 0.98 | 1.57 | 0.078 | 1.37 | 1.06 | 1.77 | 0.017 |
| Unfavorable | 1.82 | 1.46 | 2.28 | 1.57×10^-07^ | 2.41 | 1.90 | 3.07 | 6.88×10^-13^ |
| *P* for trend |  |  |  | 2.42×10^-09^ |  |  |  | 6.22×10^-15^ |
| Model adjusted for age, assessment center, genetic risk and all six risk domains. SES, socioeconomic status; HR, hazard ratio; CI, confidence interval. | | | | | | | | |

| **Supplementary Table 13. Risk of incident late onset schizophrenia according to profiles of 6 risk domains stratified by follow-up duration** | | | | | | | | |
| --- | --- | --- | --- | --- | --- | --- | --- | --- |
| **Subgroup** | **≥5 years** | | | | **≥10 years** | | | |
|  | **HR** | **5% CI** | **95% CI** | ***P*-value** | **HR** | **5% CI** | **95% CI** | ***P*-value** |
| **Medical history** | | | | | | | | |
| Favorable | 1.00 |  |  | Reference | 1.00 |  |  | Reference |
| Intermediate | 0.97 | 0.79 | 1.19 | 0.781 | 0.94 | 0.70 | 1.27 | 0.674 |
| Unfavorable | 1.81 | 1.54 | 2.12 | 8.42×10^-13^ | 1.69 | 1.33 | 2.14 | 1.48×10^-05^ |
| *P* for trend |  |  |  | 5.20×10^-15^ |  |  |  | 2.99×10^-06^ |
| **Local environment** | | | | | | | | |
| Favorable | 1.00 |  |  | Reference | 1.00 |  |  | Reference |
| Intermediate | 1.27 | 1.06 | 1.51 | 0.009 | 1.30 | 0.99 | 1.70 | 0.055 |
| Unfavorable | 1.45 | 1.22 | 1.72 | 2.59×10^-05^ | 1.68 | 1.30 | 2.17 | 8.42×10^-05^ |
| *P* for trend |  |  |  | 4.29×10^-05^ |  |  |  | 7.05×10^-05^ |
| **Lifestyles** | | | | | | | | |
| Favorable | 1.00 |  |  | Reference | 1.00 |  |  | Reference |
| Intermediate | 1.13 | 0.91 | 1.39 | 0.261 | 1.32 | 0.97 | 1.78 | 0.073 |
| Unfavorable | 1.90 | 1.57 | 2.31 | 3.86×10^-11^ | 1.86 | 1.40 | 2.48 | 1.85×10^-05^ |
| *P* for trend |  |  |  | 6.67×10^-15^ |  |  |  | 2.66×10^-06^ |
| **Mental health** | | | | | | | | |
| Favorable | 1.00 |  |  | Reference | 1.00 |  |  | Reference |
| Intermediate | 1.16 | 0.92 | 1.46 | 0.203 | 1.37 | 0.98 | 1.91 | 0.063 |
| Unfavorable | 3.42 | 2.82 | 4.14 | 3.47×10^-36^ | 3.55 | 2.67 | 4.73 | 4.89×10^-18^ |
| *P* for trend |  |  |  | 4.30×10^-48^ |  |  |  | 1.23×10^-22^ |
| **Physical measures** | | | | | | | | |
| Favorable | 1.00 |  |  | Reference | 1.00 |  |  | Reference |
| Intermediate | 1.36 | 1.12 | 1.65 | 0.002 | 1.43 | 1.07 | 1.91 | 0.014 |
| Unfavorable | 1.77 | 1.39 | 2.26 | 4.91×10^-06^ | 1.67 | 1.15 | 2.42 | 0.006 |
| *P* for trend |  |  |  | 2.55×10^-06^ |  |  |  | 0.007 |
| **SES** | | | | | | | | |
| Favorable | 1.00 |  |  | Reference | 1.00 |  |  | Reference |
| Intermediate | 1.16 | 0.95 | 1.42 | 0.140 | 1.16 | 0.87 | 1.55 | 0.315 |
| Unfavorable | 1.76 | 1.46 | 2.13 | 4.04×10^-09^ | 1.58 | 1.20 | 2.09 | 0.001 |
| *P* for trend |  |  |  | 1.81×10^-11^ |  |  |  | 2.56×10^-04^ |
| Model adjusted for age, assessment center, genetic risk and all six risk domains. SES, socioeconomic status; HR, hazard ratio; CI, confidence interval. | | | | | | | | |

| **Supplementary Table 14. Risk of incident late onset schizophrenia according to profiles of 6 risk domains stratified by genetic risk** | | | | | | | | | | | | |
| --- | --- | --- | --- | --- | --- | --- | --- | --- | --- | --- | --- | --- |
| **Subgroup** | **Low genetic risk** | | | | **Moderate genetic risk** | | | | **High genetic risk** | | | |
|  | **HR** | **5% CI** | **95% CI** | ***P*-value** | **HR** | **5% CI** | **95% CI** | ***P*-value** | **HR** | **5% CI** | **95% CI** | ***P*-value** |
| **Medical history** | | | | | | | | | | | | |
| Favorable | 1.00 |  |  | Reference | 1.00 |  |  | Reference | 1.00 |  |  | Reference |
| Intermediate | 0.99 | 0.67 | 1.47 | 0.966 | 0.78 | 0.56 | 1.10 | 0.158 | 1.07 | 0.84 | 1.37 | 0.577 |
| Unfavorable | 2.20 | 1.63 | 2.98 | 2.68×10^-07^ | 1.66 | 1.28 | 2.15 | 1.13×10^-04^ | 1.95 | 1.61 | 2.37 | 1.33×10^-11^ |
| *P* for trend |  |  |  | 8.15×10^-08^ |  |  |  | 3.70×10^-05^ |  |  |  | 7.06×10^-13^ |
| **Local environment** | | | | | | | | | | | | |
| Favorable | 1.00 |  |  | Reference | 1.00 |  |  | Reference | 1.00 |  |  | Reference |
| Intermediate | 1.48 | 1.08 | 2.03 | 0.015 | 1.06 | 0.80 | 1.39 | 0.702 | 1.24 | 0.99 | 1.54 | 0.058 |
| Unfavorable | 1.44 | 1.04 | 1.98 | 0.026 | 1.21 | 0.92 | 1.59 | 0.171 | 1.46 | 1.18 | 1.80 | 3.90×10^-04^ |
| *P* for trend |  |  |  | 0.031 |  |  |  | 0.159 |  |  |  | 4.92×10^-04^ |
| **Lifestyles** | | | | | | | | | | | | |
| Favorable | 1.00 |  |  | Reference | 1.00 |  |  | Reference | 1.00 |  |  | Reference |
| Intermediate | 0.99 | 0.69 | 1.43 | 0.978 | 1.13 | 0.81 | 1.58 | 0.465 | 1.21 | 0.94 | 1.57 | 0.138 |
| Unfavorable | 1.48 | 1.08 | 2.03 | 0.016 | 1.89 | 1.41 | 2.55 | 2.38×10^-05^ | 1.98 | 1.57 | 2.49 | 9.09×10^-09^ |
| *P* for trend |  |  |  | 0.004 |  |  |  | 1.27×10^-06^ |  |  |  | 3.25×10^-11^ |
| **Mental health** | | | | | | | | | | | | |
| Favorable | 1.00 |  |  | Reference | 1.00 |  |  | Reference | 1.00 |  |  | Reference |
| Intermediate | 1.31 | 0.89 | 1.93 | 0.176 | 1.29 | 0.84 | 1.96 | 0.245 | 1.12 | 0.84 | 1.50 | 0.438 |
| Unfavorable | 3.37 | 2.42 | 4.68 | 4.83×10^-13^ | 5.50 | 3.90 | 7.77 | 3.17×10^-22^ | 3.38 | 2.66 | 4.29 | 2.33×10^-23^ |
| *P* for trend |  |  |  | 1.71×10^-14^ |  |  |  | 7.13×10^-27^ |  |  |  | 2.49×10^-29^ |
| **Physical measures** | | | | | | | | | | | | |
| Favorable | 1.00 |  |  | Reference | 1.00 |  |  | Reference | 1.00 |  |  | Reference |
| Intermediate | 1.29 | 0.91 | 1.81 | 0.153 | 1.43 | 1.06 | 1.94 | 0.020 | 1.30 | 1.03 | 1.64 | 0.028 |
| Unfavorable | 1.46 | 0.93 | 2.27 | 0.097 | 1.96 | 1.31 | 2.93 | 0.001 | 1.81 | 1.35 | 2.44 | 8.45×10^-05^ |
| *P* for trend |  |  |  | 0.090 |  |  |  | 8.47×10^-04^ |  |  |  | 4.57×10^-05^ |
| **SES** | | | | | | | | | | | | |
| Favorable | 1.00 |  |  | Reference | 1.00 |  |  | Reference | 1.00 |  |  | Reference |
| Intermediate | 1.72 | 1.16 | 2.53 | 0.006 | 1.28 | 0.93 | 1.76 | 0.132 | 1.17 | 0.91 | 1.50 | 0.217 |
| Unfavorable | 2.85 | 1.96 | 4.13 | 3.44×10^-08^ | 1.68 | 1.23 | 2.29 | 9.67×10^-04^ | 2.05 | 1.63 | 2.57 | 7.49×10^-10^ |
| *P* for trend |  |  |  | 1.49×10^-09^ |  |  |  | 2.39×10^-04^ |  |  |  | 2.84×10^-12^ |
| Model adjusted for age, sex, assessment centre and all six risk domains. SES, socioeconomic status; HR, hazard ratio; CI, confidence interval. | | | | | | | | | | | | |

| **Supplementary Table 15. Risk of incident late onset schizophrenia according to profiles of 5 risk domains.** The analysis was performed based on model 2 (adjusted for age, sex, assessment center, genetic risk and all six risk domains). SES, socioeconomic status; HR, hazard ratio; CI, confidence interval. | | | | |
| --- | --- | --- | --- | --- |
| **Subgroup** | **HR** | **5% CI** | **95% CI** | ***P*-value** |
| **Medical history** | | | | |
| Favorable | 1.00 |  |  | Reference |
| Intermediate | 1.03 | 0.86 | 1.23 | 0.751 |
| Unfavorable | 2.20 | 1.92 | 2.53 | 4.16×10^-29^ |
| *P* for trend |  |  |  | 1.10×10^-32^ |
| **Local environment** | | | | |
| Favorable | 1.00 |  |  | Reference |
| Intermediate | 1.25 | 1.07 | 1.45 | 0.004 |
| Unfavorable | 1.42 | 1.23 | 1.64 | 2.94×10^-06^ |
| *P* for trend |  |  |  | 5.08×10^-06^ |
| **Lifestyles** | | | | |
| Favorable | 1.00 |  |  | Reference |
| Intermediate | 1.21 | 1.01 | 1.44 | 0.037 |
| Unfavorable | 2.16 | 1.83 | 2.54 | 1.94×10^-20^ |
| *P* for trend |  |  |  | 3.82×10^-26^ |
| **Physical measures** | | | | |
| Favorable | 1.00 |  |  | Reference |
| Intermediate | 1.40 | 1.19 | 1.64 | 5.34×10^-05^ |
| Unfavorable | 1.94 | 1.58 | 2.39 | 2.99×10^-10^ |
| *P* for trend |  |  |  | 1.30×10^-10^ |
| **SES** | | | | |
| Favorable | 1.00 |  |  | Reference |
| Intermediate | 1.39 | 1.16 | 1.65 | 2.69×10^-04^ |
| Unfavorable | 2.42 | 2.05 | 2.86 | 1.24×10^-25^ |
| *P* for trend |  |  |  | 1.15×10^-30^ |
|  | | | | |

| **Supplementary Table 16. Weighted and unweighted PAF and communality of 6 domains and PRS.** | | | | | | | |
| --- | --- | --- | --- | --- | --- | --- | --- |
|  | **Model 1** | | |  | **Model 2** | | |
| **Domains** | **Unweighted PAF** | **Communality** | **Weighted PAF** |  | **Unweighted PAF** | **Communality** | **Weighted PAF** |
| Medical history | 24.8 | 23 | 11.7 |  | 32.8 | 15.4 | 11.4 |
| Local environment | 7.8 | 31.1 | 3.7 |  | 21.8 | 11.1 | 7.6 |
| Lifestyles | 19.9 | 23.8 | 9.4 |  | 37.0 | 24.4 | 12.8 |
| Mental health | 45.8 | 16.7 | 21.6 |  | 55.8 | 38.7 | 19.3 |
| Physical measures | 11.4 | 22.9 | 5.4 |  | 30.1 | 28.6 | 10.4 |
| SES | 20.4 | 28.5 | 9.6 |  | 41.4 | 31.5 | 14.3 |
| PRS | 22.1 | 54 | 10.5 |  | 32.1 | 50.2 | 11.1 |
| Overall weighted PAF |  |  | 71.9 |  |  |  | 86.9 |
| In model 1, we shifted the unfavorable profile to intermediate and favorable ones. In model 2, we shifted all factors to the favorable profile. All model adjusted for age, sex, assessment center and genetic risk. Weighted PAF was calculated after considering overlap between risk factors. PAF, population attributable fraction; SES, socioeconomic status; PRS, polygenic risk score. | | | | | | | |

| **Supplementary Table 17. Weighted and unweighted PAF and communality of 6 domains stratified by age.** | | | | | | | | | | | | | | | |
| --- | --- | --- | --- | --- | --- | --- | --- | --- | --- | --- | --- | --- | --- | --- | --- |
|  | **Aged <65 years old** | | | | | | |  | **Aged ≥65 years old** | | | | | | |
|  | **Model 1** | | |  | **Model 2** | | |  | **Model 1** | | |  | **Model 2** | | |
| **Domains** | **Unweighted PAF** | **Communality** | **Weighted PAF** |  | **Unweighted PAF** | **Communality** | **Weighted PAF** |  | **Unweighted PAF** | **Communality** | **Weighted PAF** |  | **Unweighted PAF** | **Communality** | **Weighted PAF** |
| Medical history | 22.0 | 19.2 | 12.3 |  | 31.0 | 21.2 | 11.3 |  | 30.8 | 17.5 | 16.6 |  | 33.8 | 14.4 | 14.4 |
| Local environment | 7.1 | 5.1 | 3.9 |  | 19.4 | 10.8 | 7.1 |  | 10.9 | 5.8 | 5.9 |  | 28.5 | 10.9 | 12.2 |
| Lifestyles | 19.6 | 23.6 | 11.0 |  | 36.6 | 24.0 | 13.4 |  | 20.4 | 24.7 | 11.0 |  | 35.1 | 24.0 | 15.0 |
| Mental health | 49.4 | 13.0 | 27.6 |  | 56.5 | 63.3 | 20.7 |  | 37.2 | 11.7 | 20.1 |  | 53.4 | 4.6 | 22.8 |
| Physical measures | 7.4 | 11.5 | 4.1 |  | 29.2 | 48.3 | 10.7 |  | 22.5 | 12.0 | 12.1 |  | 26.4 | 17.0 | 11.3 |
| SES | 20.8 | 27.7 | 11.6 |  | 40.4 | 32.4 | 14.8 |  | 11.9 | 28.4 | 6.4 |  | 29.7 | 29.2 | 12.7 |
| Overall weighted PAF |  |  | 70.5 |  |  |  | 77.9 |  |  |  | 72.1 |  |  |  | 88.3 |
| In model 1, we shifted the unfavorable profile to intermediate and favorable ones. In model 2, we shifted all factors to the favorable profile. All model adjusted for sex, genetic risk and assessment center. Weighted PAF was calculated after considering overlap between risk factors. PAF, population attributable fraction; SES, socioeconomic status. | | | | | | | | | | | | | | | |

| **Supplementary Table 18. Weighted and unweighted PAF and communality of 6 domains stratified by sex.** | | | | | | | | | | | | | | | |
| --- | --- | --- | --- | --- | --- | --- | --- | --- | --- | --- | --- | --- | --- | --- | --- |
|  | **Female** | | | | | | |  | **Male** | | | | | | |
|  | **Model 1** | | |  | **Model 2** | | |  | **Model 1** | | |  | **Model 2** | | |
| **Domains** | **Unweighted PAF** | **Communality** | **Weighted PAF** |  | **Unweighted PAF** | **Communality** | **Weighted PAF** |  | **Unweighted PAF** | **Communality** | **Weighted PAF** |  | **Unweighted PAF** | **Communality** | **Weighted PAF** |
| Medical history | 24.5 | 21.5 | 13.5 |  | 27.9 | 27.2 | 11.3 |  | 24.9 | 17.8 | 14.2 |  | 38.1 | 15.3 | 15.9 |
| Local environment | 9.2 | 3.0 | 5.1 |  | 20.2 | 79.8 | 8.2 |  | 5.6 | 5.5 | 3.2 |  | 22.4 | 8.8 | 9.3 |
| Lifestyles | 20.2 | 21.6 | 11.1 |  | 34.1 | 22.3 | 13.8 |  | 18.8 | 22.7 | 10.7 |  | 38.8 | 22.3 | 16.2 |
| Mental health | 44.6 | 9.8 | 24.5 |  | 55.6 | 8.2 | 22.5 |  | 46.6 | 9.5 | 26.7 |  | 54.9 | 5.1 | 22.9 |
| Physical measures | 14.7 | 17.6 | 8.1 |  | 32.7 | 31.0 | 13.2 |  | 2.4 | 18.5 | 1.4 |  | 16.3 | 20.5 | 6.8 |
| SES | 16.7 | 26.5 | 9.2 |  | 38.0 | 31.6 | 15.3 |  | 23.9 | 26.0 | 13.7 |  | 43.0 | 28.0 | 17.9 |
| Overall weighted PAF |  |  | 71.5 |  |  |  | 84.2 |  |  |  | 69.9 |  |  |  | 89.2 |
| In model 1, we shifted the unfavorable profile to intermediate and favorable ones. In model 2, we shifted all factors to the favorable profile. All model adjusted for age, genetic risk and assessment center. Weighted PAF was calculated after considering overlap between risk factors. PAF, population attributable fraction; SES, socioeconomic status. | | | | | | | | | | | | | | | |

| **Supplementary Table 19. Weighted and unweighted PAF and communality of 6 domains stratified by follow-up duration** | | | | | | | | | | | | | | | |
| --- | --- | --- | --- | --- | --- | --- | --- | --- | --- | --- | --- | --- | --- | --- | --- |
|  | **≥5 years** | | | | | | |  | **≥10 years** | | | | | | |
|  | **Model 1** | | |  | **Model 2** | | |  | **Model 1** | | |  | **Model 2** | | |
| **Domains** | **Unweighted PAF** | **Communality** | **Weighted PAF** |  | **Unweighted PAF** | **Communality** | **Weighted PAF** |  | **Unweighted PAF** | **Communality** | **Weighted PAF** |  | **Unweighted PAF** | **Communality** | **Weighted PAF** |
| Medical history | 22.4 | 19.7 | 12.4 |  | 30.1 | 18.0 | 11.1 |  | 20.3 | 19.4 | 11.8 |  | 25.2 | 18.1 | 9.4 |
| Local environment | 9.4 | 4.3 | 5.2 |  | 24.2 | 9.6 | 9.0 |  | 14.2 | 4.3 | 8.3 |  | 28.2 | 9.7 | 10.5 |
| Lifestyles | 21.9 | 23.8 | 12.2 |  | 37.2 | 24.2 | 13.8 |  | 17.3 | 23.8 | 10.1 |  | 37.5 | 24.2 | 14.0 |
| Mental health | 43.2 | 11.0 | 24.1 |  | 52.0 | 70.5 | 19.2 |  | 41.2 | 11.0 | 24.0 |  | 53.6 | 70.5 | 19.9 |
| Physical measures | 11.0 | 13.0 | 6.1 |  | 30.6 | 45.6 | 11.3 |  | 7.2 | 13.3 | 4.2 |  | 30.9 | 45.2 | 11.5 |
| SES | 17.3 | 28.3 | 9.6 |  | 34.3 | 32.1 | 12.7 |  | 13.8 | 28.3 | 8.0 |  | 29.5 | 32.3 | 11.0 |
| Overall weighted PAF |  |  | 69.7 |  |  |  | 77.1 |  |  |  | 66.4 |  |  |  | 76.3 |
| In model 1, we shifted the unfavorable profile to intermediate and favorable ones. In model 2, we shifted all factors to the favorable profile. All model adjusted for age, genetic risk and assessment center. Weighted PAF was calculated after considering overlap between risk factors. PAF, population attributable fraction; SES, socioeconomic status. | | | | | | | | | | | | | | | |

| **Supplementary Table 20_1 Weighted and unweighted PAF and communality of 6 domains stratified by genetic risk.** | | | | | | | | | | | | | | | |
| --- | --- | --- | --- | --- | --- | --- | --- | --- | --- | --- | --- | --- | --- | --- | --- |
|  | **Low genetic risk** | | | | | | |  | **Medium genetic risk** | | | | | | |
|  | **Model 1** | | |  | **Model 2** | | |  | **Model 1** | | |  | **Model 2** | | |
| **Domains** | **Unweighted PAF** | **Communality** | **Weighted PAF** |  | **Unweighted PAF** | **Communality** | **Weighted PAF** |  | **Unweighted PAF** | **Communality** | **Weighted PAF** |  | **Unweighted PAF** | **Communality** | **Weighted PAF** |
| Medical history | 29.6 | 20.2 | 16.8 |  | 32.8 | 15.6 | 13.3 |  | 22.6 | 19.7 | 12.7 |  | 24.6 | 15.6 | 10.6 |
| Local environment | 5.3 | 3.5 | 3.0 |  | 22.3 | 9.1 | 9.1 |  | 6.3 | 4.3 | 3.5 |  | 12.4 | 8.9 | 5.3 |
| Lifestyles | 15.1 | 23.8 | 8.5 |  | 37.3 | 24.2 | 15.2 |  | 21.1 | 23.9 | 11.8 |  | 36.8 | 24.2 | 15.8 |
| Mental health | 37.5 | 10.3 | 21.2 |  | 56.3 | 6.2 | 22.9 |  | 56.0 | 10.9 | 31.5 |  | 64.6 | 6.0 | 27.8 |
| Physical measures | 5.6 | 13.5 | 3.2 |  | 30.4 | 14.3 | 12.4 |  | 12.6 | 13.0 | 7.1 |  | 33.8 | 14.6 | 14.6 |
| SES | 24.8 | 28.7 | 14.0 |  | 41.7 | 30.6 | 16.9 |  | 13.0 | 28.3 | 7.3 |  | 35.3 | 30.7 | 15.2 |
| Overall weighted PAF |  |  | 66.6 |  |  |  | 89.8 |  |  |  | 73.9 |  |  |  | 89.3 |
| In model 1, we shifted the unfavorable profile to intermediate and favorable ones. In model 2, we shifted all factors to the favorable profile. All model adjusted for age, sex, and assessment center. Weighted PAF was calculated after considering overlap between risk factors. PAF, population attributable fraction; SES, socioeconomic status. | | | | | | | | | | | | | | | |

| **Supplementary Table 20_2 Weighted and unweighted PAF and communality of 6 domains stratified by genetic risk.** | | | | | | | |
| --- | --- | --- | --- | --- | --- | --- | --- |
|  | **High genetic risk** | | | | | | |
|  | **Model 1** | | |  | **Model 2** | | |
| **Domains** | **Unweighted PAF** | **Communality** | **Weighted PAF** |  | **Unweighted PAF** | **Communality** | **Weighted PAF** |
| Medical history | 24.2 | 19.8 | 12.8 |  | 35.5 | 15.4 | 14.3 |
| Local environment | 9.8 | 4.8 | 5.2 |  | 24.2 | 9.6 | 9.7 |
| Lifestyles | 21.9 | 23.9 | 11.6 |  | 41.7 | 24.3 | 16.7 |
| Mental health | 44.8 | 11.8 | 23.8 |  | 53.0 | 7.3 | 21.3 |
| Physical measures | 13.4 | 11.7 | 7.1 |  | 29.1 | 13.2 | 11.7 |
| SES | 23.1 | 27.9 | 12.3 |  | 40.0 | 30.3 | 16.1 |
| Overall weighted PAF |  |  | 72.9 |  |  |  | 89.7 |
| In model 1, we shifted the unfavorable profile to intermediate and favorable ones. In model 2, we shifted all factors to the favorable profile. All model adjusted for age, sex, and assessment center. Weighted PAF was calculated after considering overlap between risk factors. PAF, population attributable fraction; SES, socioeconomic status. | | | | | | | |

| **Supplementary Table 21. Weighted and unweighted PAF and communality of 5 domains.** | | | | | | | |
| --- | --- | --- | --- | --- | --- | --- | --- |
|  | **Model 1** | | |  | **Model 2** | | |
| **Domains** | **Unweighted PAF** | **Communality** | **Weighted PAF** |  | **Unweighted PAF** | **Communality** | **Weighted PAF** |
| Medical history | 29.4 | 22.0 | 16.7 |  | 34.9 | 15.8 | 16.2 |
| Local environment | 8.7 | 4.8 | 4.9 |  | 22.2 | 9.8 | 10.3 |
| Lifestyles | 25.1 | 25.9 | 14.3 |  | 39.5 | 25 | 18.3 |
| Physical measures | 13.5 | 15.4 | 7.7 |  | 31.2 | 16.7 | 14.4 |
| SES | 24.9 | 31.9 | 14.1 |  | 43.3 | 32.7 | 20.0 |
| Overall weighted PAF |  |  | 57.6 |  |  |  | 79.1 |
| In model 1, we shifted the unfavorable profile to intermediate and favorable ones. In model 2, we shifted all factors to the favorable profile. All model adjusted for age, sex, and assessment center. Weighted PAF was calculated after considering overlap between risk factors. PAF, population attributable fraction; SES, socioeconomic status. | | | | | | | |
